# Factors associated with attrition in a longitudinal online study: results from the HaBIDS panel

**DOI:** 10.1186/s12874-017-0408-3

**Published:** 2017-08-31

**Authors:** Nicole Rübsamen, Manas K. Akmatov, Stefanie Castell, André Karch, Rafael T. Mikolajczyk

**Affiliations:** 1 Department of Epidemiology, Helmholtz Centre for Infection Research (HZI), Inhoffenstraße 7, 38124 Braunschweig, Germany; 2 PhD Programme “Epidemiology”, Braunschweig-Hannover, Germany; 3 AG “Biomarkers for Infectious Diseases”, TWINCORE, Centre for Experimental and Clinical Infection Research, Feodor-Lynen-Str. 7, 30625 Hannover, Germany; 4 Centre for Individualized Infection Medicine, Hannover, Germany; 5 Hannover Medical School, Hannover, Germany; 6 Institute for Medical Epidemiology, Biometry, and Informatics (IMEBI), Medical Faculty of the Martin Luther University Halle-Wittenberg, Magdeburger Straße 8, 06110 Halle (Saale), Germany

**Keywords:** Attrition, Health survey, Internet, Longitudinal study, Mixed-mode, Online, Panel, Participation, Response, Withdrawal

## Abstract

**Background:**

Knowing about predictors of attrition in a panel is important to initiate early measures against loss of participants. We investigated attrition in both early and late phase of an online panel with special focus on preferences regarding mode of participation.

**Methods:**

We used data from the HaBIDS panel that was designed to investigate knowledge, attitudes, and practice regarding infections in the German general population. HaBIDS was divided into two phases: an initial phase when some participants could choose their preferred mode of participation (paper-and-pencil or online) and an extended phase when participants were asked to become members of an online panel that was not limited regarding its duration (i.e. participants initially preferring paper questionnaires switched to online participation). Using competing risks regression, we investigated two types of attrition (formal withdrawal and discontinuation without withdrawal) among online participants, separately for both phases. As potential predictors of attrition, we considered sociodemographic characteristics, physical and mental health as well as auxiliary information describing the survey process, and, in the extended phase, initial mode preference.

**Results:**

In the initial phase, higher age and less frequent Internet usage predicted withdrawal, while younger age, higher stress levels, delay in returning the consent form, and need for receiving reminder emails predicted discontinuation. In the extended phase, only need for receiving reminder emails predicted discontinuation. Numbers of withdrawal in the extended phase were too small for analysis. Initial mode preference did not predict attrition in the extended phase. Besides age, there was no evidence of differential attrition by sociodemographic factors in any phase.

**Conclusions:**

Predictors of attrition were similar in both phases of the panel, but they differed by type of attrition (withdrawal vs. discontinuation). Sociodemographic characteristics only played a minor role for both types of attrition. Need for receiving a reminder was the strongest predictor of discontinuation in any phase, but no predictor of withdrawal. We found predictors of attrition, which can be identified already in the early phase of a panel so that countermeasures (e.g. special incentives) can be taken.

**Electronic supplementary material:**

The online version of this article (10.1186/s12874-017-0408-3) contains supplementary material, which is available to authorized users.

## Background

In 2005, Eysenbach argued that a “science of attrition” is needed [1]. He distinguished two types of attrition in the context of interventional eHealth applications: users who formally withdraw and users who do not formally withdraw, but who are no longer using the application (discontinuation). Eysenbach states that these types of attrition can either be closely related or not, i.e. that it is possible to have low rates of withdrawal, but still many participants not using the application (he cites [2, 3] as examples). He hypothesized that a high proportion of withdrawal is a result of discontinuation. Following Eysenbach, several authors have investigated the types of attrition in eHealth interventions [4–7]. However, since eHealth interventions are mostly tailored to the individual participant, their findings about attrition might not be transferable to online panels (which do not include individualized interventions).

In online panels, the problem of attrition was studied in the context of social and political sciences [8–10], showing that sociodemographic factors as well as other factors possibly changing over time, e.g. commitment to the survey or panel fatigue, were associated with attrition [8]. Data on attrition in health-related online panels is restricted to specific research fields, e.g. syndromic surveillance [11] or changes in health among adolescents [12]. It is not clear if predictors of attrition in health-related online panels are comparable to those in panels in the context of social sciences. Knowing who is at risk of attrition in a panel is important to make efforts to keep those participants at risk engaged in the panel and to be able to predict bias as panel attrition can limit generalizability and make analyses of repeated (follow-up) measurements more complicated and less valid [7].

With increasing use of the internet, studies using online data collection, especially with mobile applications, will likely become more common in future; still it is not clear if those who would – given the choice – prefer other modes of participation display distinct patterns of attrition when participating in an online panel.

We took advantage of data from a longitudinal panel on health-related issues and investigated two questions: 1) which factors are associated with survey attrition, and 2) if attrition differs between those who initially participated in the panel by paper-and-pencil and agreed to switch to online participation later and those who participated online from the beginning. We differentiated between withdrawal and discontinuation because we hypothesized that these two entities represent different groups of participants that have different reasons to stop participating in the study.

## Methods

### Recruitment

This analysis was based on the population-based, longitudinal Hygiene and Behaviour Infectious Diseases Study (HaBIDS), conducted in four regions of Lower Saxony, Germany and designed to assess knowledge, attitudes, and practice related to infectious diseases and to investigate effects of survey design [13, 14]. Potential participants between 15 and 69 years of age were drawn by means of proportional stratified random sampling from the regional population registries. We sampled individuals proportionally from 22 age-sex strata (11 age groups multiplied by two sexes). We did not explicitly aim to include or exclude immigrants, but only included the fraction of immigrants that lived in the study regions at the time of sampling. Participants in the study were asked if they were born in Germany. We did not include a question about first or second degree immigration.

In a first wave (January 2014), we used a mixed-mode approach, i.e. we offered a choice between paper-and-pencil and online participation to 16,895 potential participants living in the regions Braunschweig and Vechta. We refer to individuals who chose paper-and-pencil participation as “mixed-mode: paper” group and to individuals who chose online participation as “mixed-mode: online” group (Table 1 gives an overview of the groups defined throughout this article). In a second wave (April 2014), we offered only online participation to 10,000 newly invited potential participants living in Salzgitter and Wolfenbüttel. We refer to participants from this second wave as “online-only” group. All potential participants received one invitation letter via land mail and no further reminder letters. To stimulate continuing participation, we offered bimonthly feedbacks about results of the study.

**Table 1:** Definition of expressions used throughout the article

Expression	Definition
Phase 1: Initial phase	Study phase between initial invitation to the HaBIDS panel and invitation to the timely unlimited panel (includes questionnaires A to K)
“Mixed-mode: paper” group	group of individuals who chose to participate via paper-and-pencil questionnaires in the mixed-mode survey
“Mixed-mode: online” group	group of individuals who chose to participate via online questionnaires in the mixed-mode survey
“Online-only” group	group of individuals who participate in the online-only survey (participation only possible via online questionnaires)
Initial online participants	group of individuals who participate via online questionnaires in both surveys, i.e. union of the two groups “mixed-mode: online” and “online-only”
Phase 2: Extended phase (online panel)	Study phase between invitation to the timely unlimited panel and latest questionnaire (includes questionnaires L to P)
Former paper participants	“mixed-mode: paper” who continued participating in the extended phase, i.e. who switched from participation via paper-and-pencil to online questionnaires
All-time online participants	online participants who continued participating in the extended phase
Both phases	
Withdrawers	participants who formally withdraw from HaBIDS
Discontinuers	participants who do not formally withdraw, but who are no longer filling in questionnaires (this condition is fulfilled if at least two consecutive questionnaires are missing and the participant does not return to the study; see for Additional file 4 that illustrates discontinuation)
Regular users	participants who neither withdraw nor discontinue

### Study phases

We stated in the invitation letter to the HaBIDS study that the study would consist of nine online or two paper-and-pencil questionnaires, i.e. that the study would be limited regarding its duration. Between January 2014 and July 30th, 2015 (“initial phase”), online participants, i.e. participants in “mixed-mode: online” and “online-only”, received questionnaires A to K (Additional file 1 illustrates the timing, length, topics, and response rates of all questionnaires; Additional file 2 provides the English translation of all questionnaires). The “online-only” participants received questionnaires A and B at the same time to compensate for their delayed recruitment into the panel. The “mixed-mode: paper” participants received two paper questionnaires: the first one included the topics A, B, C, D and the second one included E, G, H, I, K. Questionnaires F and J were sent to online participants only because they were added in the course of the study to survey participants about recent topics (Ebola, Influenza).

In an extended phase starting on July 30th, 2015, we invited all participants of the initial phase (who had not formally withdrawn so far) to continue with the study and become part of the HaBIDS online panel that was not limited regarding its duration. Invitation to this extended phase stated that new questionnaires would be sent to panel members every two to three months. For “mixed-mode: paper” participants, becoming part of this online panel meant to switch the mode of participation. If a participant did not want to become part of the HaBIDS online panel, we asked about the reasons for this decision in a multiple choice question. All panel members received five questionnaires (questionnaire L to P, Additional file 1) between August 2015 and May 2016.

For each online questionnaire (A to P), we sent a single reminder (email) to participants who had not filled in the questionnaire within two weeks after the initial invitation. In each email that the participants received (invitations to questionnaires as well as reminder emails), participants were told explicitly that if they wish, they can withdraw from the study and further reminders by replying to the email.

A flow diagram that summarizes recruitment process and study phases is presented in Additional file 3.

### Research questions

**Fig. 1:**
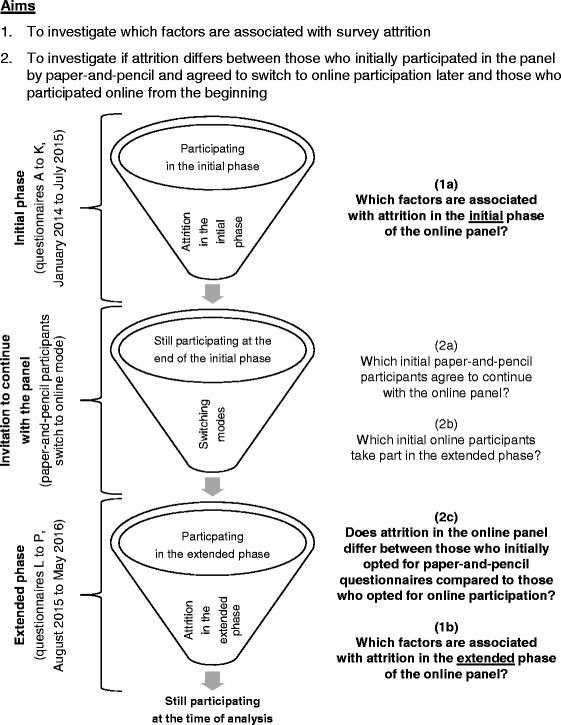
Overview of the research questions

Figure 1 presents an overview of our research questions, on which we elaborate below.

Our first aim was to investigate which factors are associated with survey attrition. We were especially interested in investigating if the same variables were associated with attrition in both the early and the late phase of an online panel. If they would differ, then it would be advisable to tailor countermeasures, e.g. special incentives, to the different phases of an online panel. We investigated (1a) which factors are associated with attrition in the initial phase of the online panel and (1b) which factors are associated with attrition in the extended phase of the online panel.

Our second aim was to investigate if attrition differs between those who initially participated in the study by paper-and-pencil and agreed to switch to online participation later, and those who participated online from the beginning. To assess if there is selection bias in the extended phase, we investigated if participants with certain sociodemographic characteristics were less likely to be transferred from the initial study to the online panel. We investigated (2a) which initial paper-and-pencil participants agree to continue with the online panel, (2b) which initial online participants take part in the extended phase and (2c) if attrition in the online panel differs between those who initially opted for paper-and-pencil questionnaires compared to those who opted for online participation.

### Definition of attrition

We divided the online participants in the initial phase as well as the online panel members in the extended phase into three mutually exclusive outcome groups: withdrawers (those who formally revoked their participation), discontinuers (those who did not fill in at least two consecutive questionnaires and remained non-responder; see for Additional file 4 that illustrates discontinuation), and regular users (participants who neither withdrew nor discontinued participation). According to the method proposed by Eysenbach [1], we restricted the investigated groups to those participants who had filled in the first questionnaire of the corresponding phase, i.e. questionnaire A or AB in the initial phase and questionnaire L in the extended phase, respectively.

The date of withdrawal was defined as the date of invitation to the questionnaire in relation to which withdrawal was declared. The date of discontinuation was defined as the date of invitation to the first questionnaire that was not filled in. In few cases, email delivery failed at some point during the study so that participants did no longer receive invitation emails. In these cases, the participant was excluded completely from the analyses of the respective phase, i.e. initial or extended phase.

### Predictors of attrition

As potential predictors of attrition, we considered sociodemographic characteristics, self-reported physical and mental health as well as metadata (auxiliary information describing the survey process). Sociodemographic characteristics comprised age, sex, marital status (married, unmarried, divorced, widowed), highest completed educational level (lower secondary education or apprenticeship, still at upper secondary school, university entrance qualification [through upper secondary education or vocational school], and university degree), and frequency of Internet usage (daily and less than daily).

Data about physical and mental health included self-rated health status (excellent, very good, good, fair, poor) [15], WHO-5 well-being index (sum of five items, score ranges from 0 [poor well-being] to 100 [excellent well-being]) [16], perceived stress scale (PSS, mean of four items, score ranges from 0 [very low stress level] to 16 [very high stress level]) [17], and mean score based on infections and infection-associated symptoms in the last 12 months (“ID Screen”, score ranges from 0 [no infections in the last 12 months] to 46 [more than 42 infections in the last 12 months]) [18].

Metadata included initially preferred mode of participation (online versus paper-and-pencil), time between invitation and return of the signed informed consent form (in days), and whether the participant responded only after receiving a reminder email for the first online questionnaire (i.e. questionnaire A in the initial phase and questionnaire L in the extended phase). We only looked at the need for reminders for the first questionnaire to investigate if already at this time point later attrition can be predicted.

In the analysis of potential self-selection into the extended phase (questions 2a and 2b), we also considered number of questionnaires filled in during the initial phase and total number of reminders received during the initial phase.

### Statistical analysis

We compared the study population’s composition with the target population’s (inhabitants of Lower Saxony between 15 and 69 years of age [19]) composition by dividing each population in strata of age, sex, and education and calculating the fraction of each stratum in the respective population to assess the possibility of generating generalizable estimates via post-stratification.

To answer question 1a, we analysed data from the initial phase in three steps. To investigate how predictors were associated with each type of attrition, we build separate Cox proportional hazards regression models to obtain unadjusted hazard ratios (separately for discontinuation and withdrawal) [20]. To make conclusions on cumulative risks, we then applied competing risks regression [21] in combination with the least absolute shrinkage and selection operator method (LASSO) to simultaneously select predictor variables and estimate regression parameters [22]. We built two models (R package “crrp”) to investigate if predictors differed by type of attrition: model A included discontinuation as event of interest and withdrawal as competing event while model B included withdrawal as event of interest and discontinuation as competing event. As tuning parameter, we used the λ that minimized BIC (as described in [22]). We compared models A and B regarding the selected variables. Finally, if a predictor variable was selected in both model A and B, but the associations had opposite directions for the two types of attrition, we used a nonparametric test for trend across ordered groups [23] to investigate the overall association between this predictor variable and the composite endpoint (attrition because of withdrawal or discontinuation).

To answer question 2a and 2b, we applied the LASSO in logistic regression (R package “glmnet”, dependent variable = participation in the extended phase) to the subset of paper-and-pencil or online participants, respectively (models C and D).

For answer questions 1b and 2c, we applied the LASSO to data from the extended phase. As only few participants withdrew during this phase, we used the LASSO in Cox regression (R package “glmnet”) with discontinuation as dependent variable (model E) and excluded participants who had withdrawn. To account for the effect that all-time online participants were already used to online questionnaires, we used the χ^2^ test to compare discontinuation among the former paper-and-pencil participants between questionnaires L and P and among the “online-only” participants (who were also “forced” to use the online mode) between questionnaires A and E.

We conducted complete-case analyses and considered *p* ≤ 0.05 as significant. To check the proportional hazards assumption, we plotted Schoenfeld residuals prior to Cox and competing risks regressions. To assess possible nonlinearity of continuous predictors, we estimated fractional polynomials prior to all regression analyses [24]. By including all possible predictors in the LASSO, we controlled for confounding because the LASSO retained all predictors that were somehow associated with the outcome. We did not consider interactions in the models. Statistical analyses where performed in Stata version 12 (StataCorp LP, College Station, TX, USA [25]) and R Foundation for Statistical Computing (version 3.3.3).

## Results

### Attrition among the online participants in the initial phase (question 1a)

Overall, 2379 (8.9%) of the invited individuals consented to participate in HaBIDS (935 participants in “mixed-mode: paper” group, 750 in “mixed-mode: online”, and 694 in “online-only”). Comparison of the study population’s composition with the target population’s (inhabitants of Lower Saxony between 15 and 69 years of age) composition showed that all strata defined by age, sex, and education were occupied in HaBIDS (Additional file 5). Among 2379 participants, only 109 (4.6%) had not been born in Germany.

**Fig. 2:**
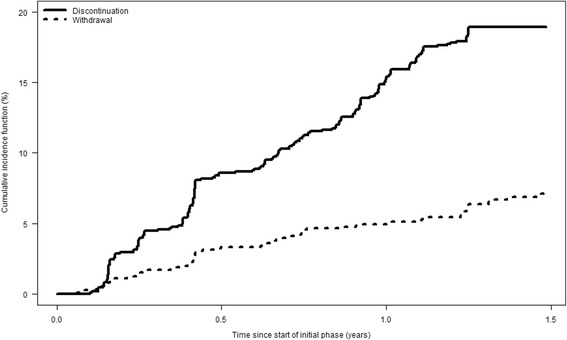
Cumulative incidence curves for discontinuation and withdrawal among the online participants in the initial phase

Data of 1127 online participants were used in the analysis of attrition in the initial phase (the remaining 317 online participants did not fill in the first online questionnaire). One fifth of the initial online participants (18.3%, *n* = 206) discontinued participation while 6.3% (*n* = 71) formally withdrew from the study, both during the initial phase of HaBIDS. For 0.9% (*n* = 10), email delivery failed at some point during the initial phase. The remaining 74.5% (*n* = 840) were classified as regular users. Figure 2 shows the cumulative incidence functions.

Among all predictors, only age showed statistically significant, univariable associations with both types of attrition (Table 2).

**Table 2:** Univariable hazard ratios for each type of attrition in the initial phase

	Discontinuation		Withdrawal	
	HR (95% CI)	*p*-value	HR (95% CI)	*p*-value
Age at baseline (per 10 years increase)	0.81 (0.73, 0.89)	<0.001	1.24 (1.04, 1.47)	0.02
Sex				
Male	Reference		Reference	
Female	0.91 (0.69, 1.21)	0.54	0.84 (0.52, 1.34)	0.46
Marital status				
Married	Reference		Reference	
Unmarried	1.32 (0.97, 1.79)	0.08	0.72 (0.41, 1.28)	0.26
Divorced/widowed	1.18 (0.73, 1.93)	0.50	0.88 (0.38, 2.06)	0.77
Highest completed educational level				
Lower secondary education or apprenticeship	1.41 (0.52, 3.84)	0.50	2.44 (0.58, 10.31)	0.22
Still at upper secondary school	0.89 (0.62, 1.29)	0.54	1.56 (0.89, 2.76)	0.12
University entrance qualification	1.13 (0.81, 1.56)	0.48	1.07 (0.59, 1.95)	0.82
University degree	Reference		Reference	
Frequency of Internet usage				
Daily	Reference		Reference	
Less than daily	1.23 (0.91, 1.67)	0.19	2.03 (1.26, 3.27)	0.004
Self-rated health status				
Fair/poor	0.93 (0.55, 1.59)	0.80	1.57 (0.67, 3.68)	0.30
Good	1.09 (0.81, 1.47)	0.56	1.73 (1.03, 2.92)	0.04
Excellent/very good	Reference		Reference	
WHO-5 well-being index (per 10 points increase)	0.96 (0.89, 1.03)	0.24	0.96 (0.85, 1.09)	0.57
PSS score (per 1 point increase)	1.07 (1.02, 1.12)	0.002	0.97 (0.89, 1.05)	0.46
ID-Screen (per 1 point increase)	1.03 (0.99, 1.07)	0.10	1.01 (0.95, 1.08)	0.73
Time between invitation and return of the filled informed consent form (per week)	1.1 (1.04, 1.17)	<0.001	0.98 (0.82, 1.16)	0.78
Had to be reminded to fill in the first online questionnaire (A)				
Yes	1.85 (1.27, 2.7)	0.001	0.82 (0.33, 2.05)	0.68
No	Reference		Reference	

The LASSO in competing risks regression (Table 3) selected higher age and less frequent Internet usage as predictors of withdrawal; and younger age, higher PSS, delay in returning the consent form, and receiving reminder emails as predictors of discontinuation.

**Table 3:** Factors associated with risk of attrition among the online participants in the initial phase

	Model A: discontinuation as event of interest, withdrawal as competing event		Model B: withdrawal as event of interest, discontinuation as competing event	
	Beta	Hazard ratio	Beta	Hazard ratio
Age at baseline (per 10 years increase)	−0.15	0.86	0.10	1.11
Frequency of Internet usage	not selected in this model			
Daily			Reference	Reference
Less than daily			0.28	1.32
PSS score (per 1 point increase)	0.04	1.04	not selected in this model	
Time between invitation and return of the filled informed consent form (per week)	0.05	1.05	not selected in this model	
Had to be reminded to fill in the first online questionnaire (A)			not selected in this model	
Yes	0.37	1.44		
No	Reference			

Variables were selected using the least absolute shrinkage and selection operator method in competing risks regression

PSS: perceived stress scale (score ranges from 0 [very low stress level] to 16 [very high stress level])

The associations between age and the outcome had opposite directions for the two types of attrition. We found that there was a trend across age groups, with 30.0% of the youngest participants (15 to 19 years) and 9.9% of the oldest participants (65 to 69 years) leaving the panel by withdrawal or discontinuation (Table 4, *p* < 0.001 for trend).

**Table 4:** Association between age and the composite endpoint (attrition because of withdrawal or discontinuation) in the initial phase

Age group (years)	Participants leaving the panel in the initial phase by withdrawal or discontinuation
15–19	12 (30.0%)
20–24	21 (28.4%)
25–29	21 (21.0%)
30–34	19 (22.6%)
35–39	27 (28.1%)
40–44	15 (12.6%)
45–49	30 (20.0%)
50–54	21 (15.7%)
55–59	19 (14.6%)
60–64	9 (10.3%)
65–69	10 (9.9%)

### Transfer of participants to the HaBIDS online panel (questions 2a and 2b)

Of 748 paper-and-pencil participants and 1319 initial online participants who had been invited to the initial HaBIDS online panel (the remaining 312 participants had formally withdrawn so far), 335 (44.8%) paper-and-pencil participants and 702 (53.2%) initial online participants consented to become timely unlimited panel members. The main reasons (in univariable analysis) for not participating in the extended phase were “no time” (12.9% of all non-participants; multiple choice was possible), “no access to the Internet” (8.2%), “too many questionnaires per year” (6.4%), and “no interest” (5.0%).

For paper-and-pencil participants, the LASSO in logistic regression selected lower PSS score, higher ID-Screen, and having returned both paper-and-pencil questionnaires during the initial phase instead of only one as predictors of agreeing to continue with online participation (Table 5). For initial online participants, the LASSO selected increasing age, being unmarried, lower PSS score, higher WHO-5 and ID-Screen scores, delay in returning the consent form, number of returned questionnaires, and increasing total number of reminder emails during the initial phase as predictors of continued participation (Table 5).

**Table 5:** Factors associated with participation in the extended phase

	Model C: paper-and-pencil participants (asked to switch to online mode)		Model D: online participants (asked to stay in the study)	
	Beta	Odds ratio	Beta	Odds ratio
Age at baseline (per 10 years increase)	not selected in this model		0.24	1.27
Marital status: unmarried vs. married	not selected in this model		0.27	1.31
WHO-5 well-being index (per 10 points increase)	not selected in this model		0.01	1.01
PSS score (per 1 point increase)	−0.02	0.98	−0.01	0.99
ID-Screen (per 1 point increase)	0.01	1.01	0.04	1.05
Time between invitation and return of the filled informed consent form (per week)	not selected in this model		0.05	1.05
Number of questionnaires returned during initial phase^a^ (per questionnaire)	0.19	1.21	0.43	1.54
Number of times a participant had to receive reminder emails during the initial phase (per reminder)	not applicable to paper-and-pencil participants		−0.27	0.76

Variables were selected using the least absolute shrinkage and selection operator method in logistic regression

ID Screen: mean score based on infections and infection-associated symptoms in the last 12 months (score ranges from 0 [no infections in the last 12 months] to 46 [more than 42 infections in the last 12 months])

PSS: perceived stress scale (score ranges from 0 [very low stress level] to 16 [very high stress level])

^a^Paper-and-pencil participants received a total of two questionnaires; online participants received a total of eleven questionnaires

### Attrition in the extended phase (questions 1b and 2c)

**Fig. 3:**
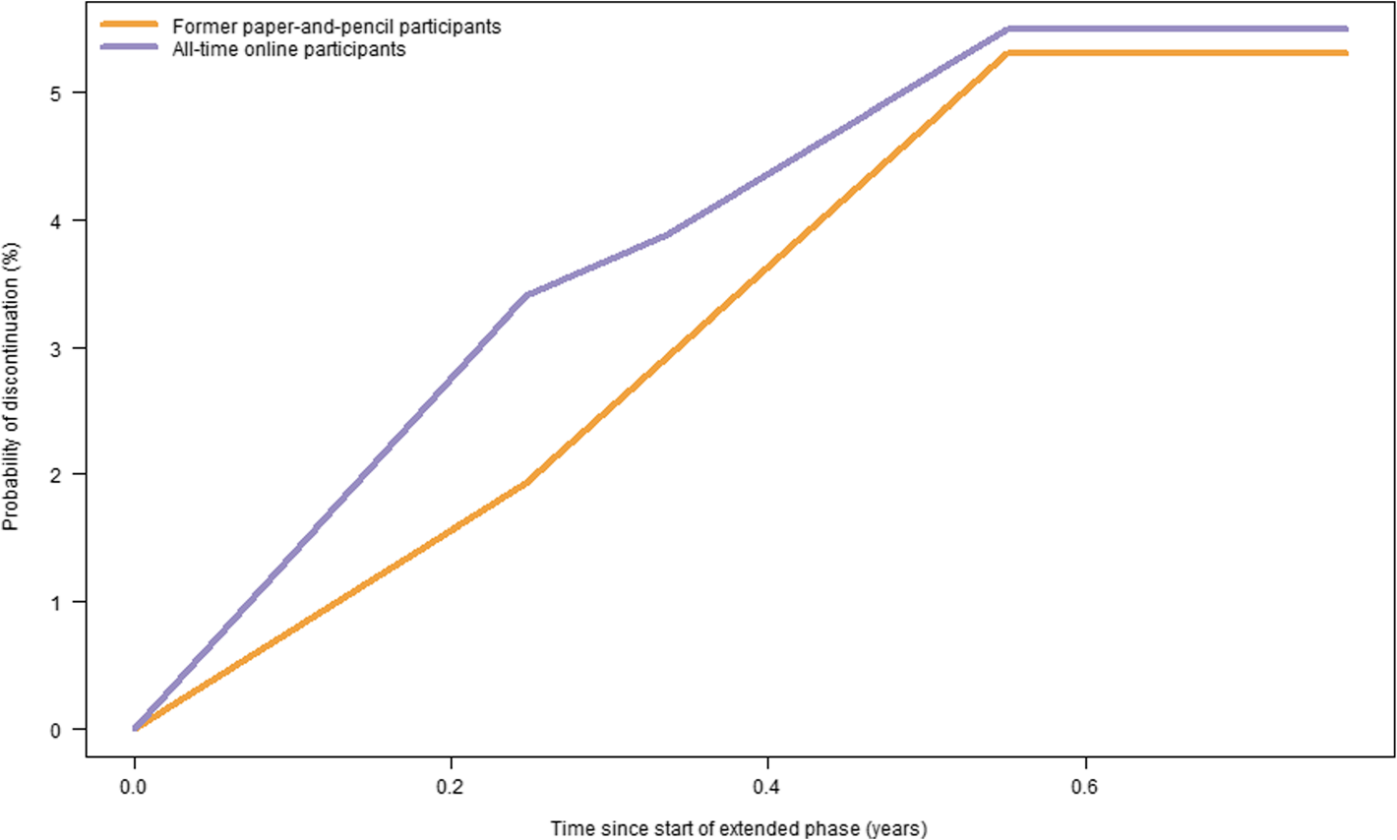
Kaplan-Meier curves for discontinuation (in the extended phase) by mode preference

More than one third (37.3%, *n* = 125) of the former paper-and-pencil participants did not fill in the first online questionnaire after their agreement to continue with the extended phase (i.e. questionnaire L) compared to 11.0% of the all-time online participants (*n* = 77, *p* < 0.001 for the comparison). Only three participants withdrew during the extended phase; these participants were excluded from the following analyses. Among all participants who filled in questionnaire L (*n* = 835), attrition in the extended phase was nearly equal between former paper-and-pencil participants and all-time online participants (5.7% vs. 5.8%, respectively, *p* = 0.91) (Fig. 3). Attrition was higher among the “online-only” participants after the first five online questionnaires of the initial phase (AB to E) compared to attrition among the former paper-and-pencil participants in the extended phase (12.4% vs. 5.7%, respectively, *p* = 0.004).

The LASSO in Cox regression selected younger age and receiving reminder emails as predictors of discontinuation (Table 6).

**Table 6:** Factors associated with risk of attrition among the online participants in the extended phase

	Model E:	
	Beta	Hazard ratio
Age at baseline (per 10 years increase)	−0.11	0.89
Had to be reminded to fill in the first online questionnaire (L)		
Yes	1.44	4.20
No	Reference	

Variables were selected using the least absolute shrinkage and selection operator method in Cox regression

## Discussion

We investigated attrition in a longitudinal panel about health and found similar predictors of attrition in different phases of the survey; however, the predictors differed by type of attrition (withdrawal vs. discontinuation). Need for sending a reminder was the strongest predictor of discontinuation, but no predictor of withdrawal. Sociodemographic characteristics only played a minor role for both types of attrition.

To our knowledge, the HaBIDS panel is the first study to investigate attrition in different phases of a panel. The need for sending a reminder was the strongest predictor for discontinuation in the initial as well as in the extended phase. Its effect size was even larger in the extended (HR 4.20) than in the initial phase (HR 1.44). This difference between phases could be caused by changes in general factors like commitment, habits, and panel fatigue (described by Lugtig [8]). Lugtig claims that commitment is especially important in the starting phase of a longitudinal study when some participants are not really convinced of the study. If participation itself, i.e. filling in the first questionnaires, does not change their commitment quickly, these participants are likely to discontinue early. Gill et al. [6] also observed the importance of commitment in their online monthly depression rescreening program of similar length as our study and with short questionnaires every month. Our analyses showed that the need of receiving a reminder for the first questionnaire predicts attrition at later questionnaires (Gill et al. did not send reminder emails, so we unfortunately cannot assess this observation in their study). The need of receiving a reminder might be influenced by commitment, but also by time constraints of the participant.

After several questionnaires, participation may have become a habit, and participants do no longer consciously think about the decision to respond, “but participate because they have done so all along” [8]. Lugtig claims that “once this habit is broken, the respondent is subsequently at a higher risk of […] attriting” [8]. Consent to continue with the online panel in the extended phase was associated with the number of prior questionnaires filled in. This could be a proxy of commitment as well as of habit (based on the participants response behaviour, we cannot distinguish these two factors) and highlights again the importance of these factors in a longitudinal panel.

Discontinuation in the timely unlimited HaBIDS online panel did not differ by previous mode of participation. However, the percentage of former paper-and-pencil participants who did not even fill in the first online questionnaire in the online panel was higher than among all-time online participants. Those participants who would generally agree to participate online, but are not used to online data collection or use the Internet very irregularly, probably drop out at the first attempt. Thus, familiarity with the Internet might be a more important factor for participation in an online panel than a true mode preference because unfamiliarity with the Internet likely leads to a choice of paper-and-pencil mode when this is offered.

Participants not using the Internet daily had a higher risk of withdrawal, but not a higher risk of discontinuation. One explanation could be that infrequent Internet users are more selective in their online activities and rather prefer to withdraw if they do not like a study. Older participants were more likely to withdraw than younger ones, but less likely to become discontinuous users. Overall, older participants left the study less often than younger ones. The former might be more conscientious so that they rather withdraw if they have no more time or interest than discontinue without any feedback to the researchers. Younger participants might be busier or get more spam emails than older ones, which might result in discontinuation instead of formal withdrawal. This is also reflected by the higher risk of discontinuation among participants with higher stress levels (higher PSS scores).

Participants at risk of discontinuation could be identified early in the study while participants at risk of withdrawal could not. We suggest that different measures could be applied to the two groups. For example, an app including questionnaires, push reminders to fill them in, and elements of gamification (applying game mechanics to non-game contexts in order to keep participants engaged [26]) might prevent passive discontinuation, but not active withdrawal. Too intrusive reminders could even stimulate withdrawal. Depending on the reason for withdrawal, which we did not assess, reassurance of data protection as well as the study’s importance for science and society and regular feedback with study results might support continuing participation. The same is true for the discontinuers. Knowing in advance that those who need more support in the beginning maintain this attitude creates the opportunity to add incentives. However, these hypotheses will have to be investigated in future studies. Further research about attrition in health-related panels is needed, for example randomized controlled trials (RCT) that examine the effect of measures against discontinuation among participants who had to be reminded to fill in the first panel questionnaire. The use of app-based mobile surveys for smartphones has already been investigated for cross-sectional studies [27], but the influence of offering app-based surveys on attrition still needs to be assessed.

### Strengths and limitations

The main strength of the HaBIDS panel is the population-based sampling. The overall response rate was below 10%, but response rates in epidemiologic studies in Germany are decreasing in general [28]. Monetary incentives can increase initial response rates, but it is unclear if they influence differential study participation [29]. In contrast, sending incentives with follow-up mailings and between study waves has been found to be effective in keeping participants engaged [30, 31]. Due to limited resources, we were not able to offer monetary incentives in our study. Consecutive waves of recruitment (sending reminders to people who do not respond in the first round of recruitment) can also increase initial response rates. However, Stang and Jöckel have shown that this approach can introduce bias itself [32].

All age-sex-education strata that exist in the target population were also occupied in HaBIDS (Additional file 5), which provides the possibility of generating generalizable estimates via post-stratification [33]. Pearl and Bareinboim [34] showed that conditional effects (association between X and Y given Z) are often transportable to other populations despite differences in their composition. However, this is only true if all relevant confounders have been accommodated. If self-selection into our study was associated with attributes that we did not assess, e.g. increased general interest in scientific studies, and if these attributes were also associated with attrition, then the associations that we found in our study might not be transportable. Unfortunately, we cannot investigate this issue based on our data. Another issue is that our study might not be generalizable to individuals older than 70 years or to special groups in the population, e.g. immigrants or multi-morbid individuals because the frequency of Internet usage and the familiarity with it might be different in these settings.

Some potential predictors of attrition were recorded only once at the beginning of the initial phase. Change over time in these predictors, e.g. increase in perceived stress, might also predict attrition. By definition, we cannot evaluate discontinuation in the last questionnaire (questionnaire K in the initial phase and P in the extended phase, respectively) so that we might underestimate the amount of discontinuation.

## Conclusions

Attrition at different phases of the online study was mainly associated with the need to receive a reminder in the early stage of a study phase. Participants who need a reminder early should be targeted with special interventions to keep them engaged in the study. Sociodemographic factors and mode preference were not associated with attrition so that bias by differential attrition with respect to those variables is unlikely.

## Additional files

Additional file 1: Timing and topics of all questionnaires in the HaBIDS study. (PDF 271 kb)

Additional file 2: English translation of the questionnaires. (PDF 1486 kb)

Additional file 3: Flow diagram of numbers of individuals at each stage of study. (PDF 137 kb)

Additional file 4: Definition of discontinuation for each online questionnaire. (PDF 237 kb)

Additional file 5: Comparison of the study population’s composition with the target population’s (inhabitants of Lower Saxony between 15 and 69 years of age) composition. Data about the target population are taken from the Census 2011 by the Federal Statistical Office of Germany [19]. (PDF 322 kb)

## Abbreviations

CI: Confidence interval
HaBIDS: Hygiene and Behaviour Infectious Diseases Study
HR: Hazard ratio
ID-Screen: Mean score based on infections and infection-associated symptoms in the last 12 months
IQR: Interquartile range
LASSO: Least absolute shrinkage and selection operator
OR: Odds ratio
PSS: Perceived stress scale
