# Supplementary material for: Factors associated with attrition in a longitudinal online study: results from the HaBIDS panel
Source: BMC Med Res Methodol. 2017 Aug 31;17:132. doi: 10.1186/s12874-017-0408-3 (PMC5580321; doi:10.1186/s12874-017-0408-3)
Supplement: Supplementary file 1 — Timing and topics of all questionnaires in the HaBIDS study. (PDF 271 kb) [file 12874_2017_408_MOESM1_ESM.pdf]

Article title: **Factors associated with attrition in a longitudinal online study: results from the HaBIDS panel**

Authors: Nicole Rübsamen; Manas K Akmatov; Stefanie Castell; André Karch; Rafael T. Mikolajczyk

Journal: BMC Medical Research Methodology

Additional file 1 – Timing and topics of all questionnaires in the HaBIDS study

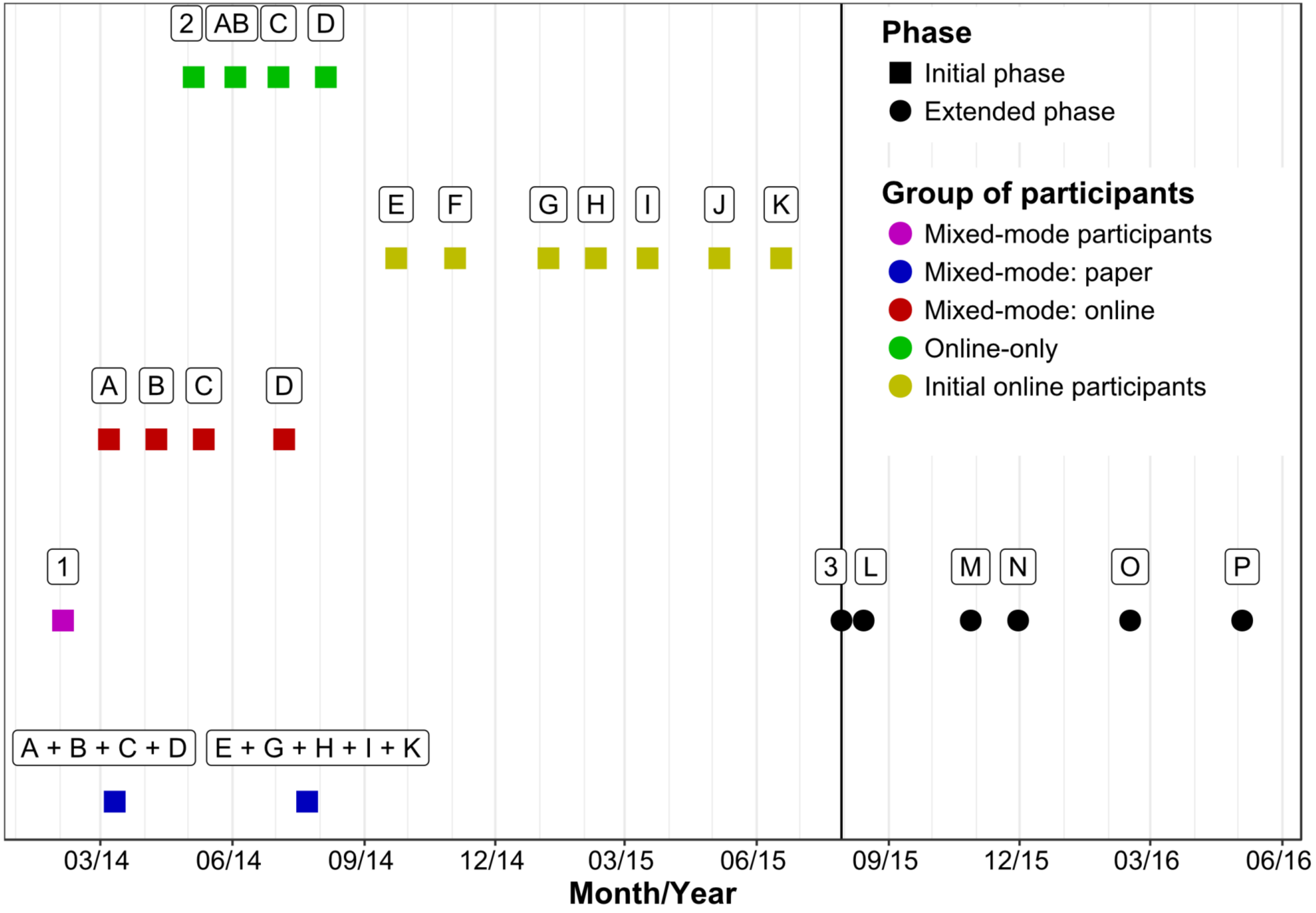

Questionnaire	Topic	Participation (% of individuals who received respective invitation email)[a]	% of participants who received reminder	Number of questions
1	First wave of initial invitation to HaBIDS panel (16,895 potential participants living in Braunschweig, Wendeburg, Bortfeld, and Vechta)			
2	Second wave of initial invitation to HaBIDS panel (10,000 potential participants living in Salzgitter and Wolfenbüttel)			
A	Frequency of infections and infection-associated symptoms in the last 12 months	100% (by definition)	2%	11
AB	Frequency of infections and infection-associated symptoms in the last 12 months; health & sociodemographic data	100% (by definition)	19%	11 + 18
B	Health & sociodemographic data	98%	14%	18
C (mixed-mode)	Prevention measures against respiratory infections	93%	18%	36
C (online-only)	Determinants for participation in community-based cohorts on infectious diseases	70%	no reminders sent	23
D (mixed-mode)	Social contact patterns (summer)	60%	57%	7 + up to 72 contacts[b]
D (online-only)	Prevention measures against respiratory infections	76%	no reminders sent	36
E	Adult vaccinations	78%	40%	24
F (online participants)	Ebola risk perception	81%	40%	27
G	Tick-borne infections	78%	36%	49
H	Antibiotics	80%	37%	23
I	Social contact patterns (winter)	68%	46%	7 + up to 72 contacts[b]
J (online participants)	Frequency of infections during the winter season & influenza vaccination (season 2014/15)	74%	42%	40
K	Sexual contact patterns & medical history	75%	37%	38

Questionnaire	Topic	Participation (% of individuals who received respective invitation email)[a]	% of participants who received reminder	Number of questions
3	Invitation to continue with the HaBIDS panel			
L	Ebola risk perception (follow-up)	100% (by definition)	10%	27
M	Tick-borne infections (follow-up)	80%	no reminders sent	23
N	Multi-drug resistant pathogens	93%	12%	27
O	Fair allocation of scarce medical resources	92%	21%	12
P	Frequency of infections during the winter season & influenza vaccination (season 2015/16)	87%	18%	40

[a]Numerator does not include participants who had withdrawn before the respective questionnaire or for whom email delivery failed.
[b]Participants were asked to fill in all contacts that they had made within 24 hours.
