# Supplementary material for: Factors associated with attrition in a longitudinal online study: results from the HaBIDS panel
Source: BMC Med Res Methodol. 2017 Aug 31;17:132. doi: 10.1186/s12874-017-0408-3 (PMC5580321; doi:10.1186/s12874-017-0408-3)
Supplement: Supplementary file 2 — English translation of the questionnaires. (PDF 1486 kb) [file 12874_2017_408_MOESM2_ESM.pdf]

Article title:
Factors associated with attrition in a longitudinal online study: results from the HaBIDS panel

Authors: Nicole Rübsamen; Manas K Akmatov; Stefanie Castell; André Karch; Rafael T. Mikolajczyk

Journal: BMC Medical Research Methodology

Additional file 2 – English translation of the questionnaires
(German questionnaires translated into English; English versions not validated for use in surveys)

A: Frequency of infections and infection-associated symptoms in the last 12 months ... 6
1: General information ... 6
2: Infections ... 7
3: Infections (continued) ... 8
B: Health & sociodemographic data ... 9
1: General health and well-being ... 9
2: General information ... 10
3: Education and profession ... 11
4: Internet ... 12
5: Pets ... 13
C (mixed-mode) / D (online-only): Prevention measures against respiratory infections ... 14
1: Preventing respiratory infections ... 14
2: Measures ... 15
3: Behavior during cold time ... 16
4: Other measures ... 17
C (online-only): Determinants for participation in community-based cohorts on infectious diseases .. 18
1: Report on disease signs ... 18
2: Report on disease signs (continued) ... 19
3: Record location ... 20
4: Biological samples ... 21
5: Data protection ... 22
6: Motivation ... 23
7: Percentage of participation ... 24
8: Acceptance of blood sampling ... 25
9: Study center ... 26
10: Household ... 27
11: Participation of children ... 28
12: Remarks ... 29
D (mixed-mode): Social contact patterns (summer) ... 30
1: Household ... 30
2: Regular contacts in work / education / school ... 31
3: Interpersonal contacts in leisure time ... 32
4: 1st contact person ... 33
5: 2nd contact person ... 33
… ... 33
75: 72nd contact person ... 33
76: Thank you for your participation ... 34

E: Adult vaccinations **35**
1: Vaccinations 35
2: Influenza 36
3: Vaccination boosts 37
4: Vaccination card 38
5: Official recommendations 39
6: Vaccinations for adults 40
7: Attitude 41
8: HPV 42
9: HPV children 43
F: Ebola risk perception **44**
1: Ebola virus disease 44
2: Probability of acquiring Ebola 45
3: Probability of acquiring Ebola (continued) 46
4: Probability of acquiring Ebola (continued) 47
5: Personal behavior and prevention measures 48
6: Personal behavior and prevention measures (continued) 49
7: Personal behavior and prevention measures (continued) 50
8: Personal behavior and prevention measures (continued) 51
9: Personal commitment 52
10: Vaccination 53
11: Activities about Ebola 54
G: Tick-borne infections **55**
1: Devices used 55
2 56
3 57
4 58
5 59
6 60
7 61
8 62
H: Antibiotics **63**
1: Questions about antibiotics 63
2: Knowledge 64
3 65
4 66
5: Remarks 67
I: Social contact patterns (winter) **68**
1: Regular contacts in work / education / school 68
2: How to register your contacts 69
3: Interpersonal contacts in leisure time 70
4: Contacts 1 - 10 71
5: Contacts 11 - 20 71

… 71
10: Contacts 61 - 70 72
11: Thank you for your participation 72
J: Frequency of infections during the winter season & influenza vaccination (season 2014/15) 73
1: Signs and symptoms 73
2: Seeking medical advice 74
3: Characteristics of the disease 75
4: Household 76
5: Household II 77
6: Influenza vaccination 78
7: Influenza vaccination II 79
8: Influenza vaccination III 80
9: Reason for influenza vaccination 81
10: Statements about influenza vaccination 82
11: Recommendation influenza vaccination 83
12: Outlook influenza vaccination 84
K: Sexual contact patterns & medical history 85
1: Information about yourself and general health 85
2: Known dieases 86
3: Cardiovascular diseases 87
4: Cancer 89
5: Metabolic diseases 96
6: Musculoskeletal diseases 98
7: Lung diseases 99
8: Allergies 100
9: Gastrointestinal or liver diseases 102
10: Skin diseases 103
11: Kidney diseases 104
12: Neurological diseases 105
13: Psychiatric diseases 106
14: Other diseases 107
15: Seeking medical advice 108
16: Smoking 109
17: Alcohol consumption 111
18: Sexual contacts 112
19: Contacts with persons of the opposite sex 113
20: Contacts with persons of the same sex 114
21: Age of contact persons 115
22: Contacts of the partner 116
23: Submit questionnaire 117
L: Ebola risk perception (follow-up) 118
1: Ebola virus disease 118
2: Ebola virus disease (continued) 119

3: Probability of acquiring Ebola ... 120
4: Probability of acquiring Ebola (continued) ... 121
5: Probability of acquiring Ebola (continued) ... 122
6: Personal behavior and prevention measures ... 123
7: Personal behavior and prevention measures (continued) ... 124
8: Personal behavior and prevention measures (continued) ... 125
9: Personal commitment ... 126
10: Personal commitment (continued) ... 127
11: Vaccination ... 128
12: Activities about Ebola ... 129
M: Tick-borne infections (follow-up) ... 130
1: Fear ... 130
2: Prevention – tick vaccination ... 131
3: Prevention – why tick vaccination? ... 132
4: Tick bites ... 133
5: Seeking medical advice ... 134
6: Last time doctor ... 135
7: Suspicion of borreliosis ... 136
8: Diagnosis of borreliosis ... 137
9: Suspicion of TBE ... 138
10: Other disease ... 139
N: Multi-drug resistant pathogens ... 140
1: Start ... 140
2: General ... 141
3: Opinion ... 142
4: Reasons ... 143
5: Animal breeding ... 144
6: Dissemination and responsibility ... 145
7: Case study 1 ... 146
8: Case study 2 ... 147
O: Fair allocation of scarce medical resources ... 148
1: Randomisation ... 148
2: Distribution of medical services ... 149
3: Distribution problem (alternative 1) ... 150
4: Distribution problem (alternative 1) ... 151
3: Distribution problem (alternative 2) ... 152
4: Distribution problem (alternative 2) ... 153
5: Question about distribution problem ... 154
P: Frequency of infections during the winter season & influenza vaccination (season 2015/16) ... 155
1: Signs and symptoms ... 155
2: Seeking medical advice ... 156
3: Characteristics of the disease ... 157
4: Household ... 158

5: Household II ... 159
6: Influenza vaccination ... 160
7: Influenza vaccination II ... 161
8: Influenza vaccination III ... 162
9: Reason for influenza vaccination ... 163
10: Statements about influenza vaccination ... 164
11: Recommendation influenza vaccination ... 165
12: Outlook influenza vaccination ... 166

▼ indicates that the question is displayed only if the respective condition is met.
[] Variable names are written in square brackets. They are not visible for the participants.

A: Frequency of infections and infection-associated symptoms in the last 12 months

1: General information

First of all, we need two details about you, so that we can adapt the following questions for you.

[age] How old are you?
enter your answer here

[sex] What is your gender?
☐ Male
☐ Female

2: Infections

In the following section we ask you to estimate how often you have had the following infections in the past 12 months.
If, for example, you have had three colds and one otitis media, then you have had a total of four infectious events of the upper respiratory tract (thus "3 to 4 times"). If you have not had an upper respiratory tract infection, please tick "Never".

[IN] In the past 12 months, how often have you had …

	Never	Once	Twice	3 to 4 times	5 to 6 times	More than 6 times	Don’t know
[IN1] … an infection of the upper respiratory tract (e.g., colds or infections of nasal sinuses, tonsils, middle ear, pharynx, larynx)?	☐	☐	☐	☐	☐	☐	☐
[IN2] … an infection of the lower respiratory tract (e.g., bronchitis or pneumonia)?	☐	☐	☐	☐	☐	☐	☐
[IN4] … an infection of the urinary bladder ("cystitis")?	☐	☐	☐	☐	☐	☐	☐

[IN1a] Have you been hospitalized for at least one of the upper respiratory tract infections (i.e., at least one night in the hospital)?
▼ IN_IN1 >= “Once”
☐ Yes
☐ No
☐ Don’t know

[IN2a] Have you been hospitalized for at least one of the lower respiratory tract infections (i.e., at least one night in the hospital)?
▼ IN_IN2 >= “Once”
☐ Yes
☐ No
☐ Don’t know

3: Infections (continued)

[IN4] In the past 12 months, how often have you had …
▼ sex == “Female”

	Never	Once	Twice	3 to 4 times	5 to 6 times	More than 6 times	Chronically (permanently)	Don’t know
[IN5] … an infection (inflammation) of the vagina?	☐	☐	☐	☐	☐	☐	☐	☐

[IN5] In the past 12 months, how often have you had …

	Never	Once	Twice	3 to 4 times	5 to 6 times	More than 6 times	Don’t know
[IN7] … cough that lasted longer than 4 weeks?	☐	☐	☐	☐	☐	☐	☐
[IN8] … fever (>38°C)?*	☐	☐	☐	☐	☐	☐	☐
[IN9] … diarrhea?**	☐	☐	☐	☐	☐	☐	☐
[IN10] … cold sores?	☐	☐	☐	☐	☐	☐	☐

* If the fever lasted longer than 1 day, please count as a one-time event; e.g. fever for 7 days = "once"
** Diarrhea is at least 3 times fluid / pulpy bowel movements within 24 hours.
If the diarrhea lasted longer than 1 day, please count as a one-time event; e.g. diarrhea for 4 days = "once"

[IN11] Have you ever had in your life …

	Never	Once	Twice	3 to 4 times	5 to 6 times	More than 6 times	Don’t know
[IN9] … herpes zoster, also called shingles?	☐	☐	☐	☐	☐	☐	☐

[IN11a] In which year or at which age did you have herpes zoster for the first time?
You only need to enter one number (year or age).
▼ IN11_IN9 >= “Once”
Year: *enter your answer here*
Age: *enter your answer here*

B: Health & sociodemographic data

1: General health and well-being

Stress and the mental state can affect the immune system. We would like to know something about your health and well-being.

[health] How would you describe your health condition in general?
☐ Excellent
☐ Very good
☐ Good
☐ Fair
☐ Poor

[fatigue] How much have you been suffering from unusual exhaustion and fatigue during the past 7 days? Please tick a number from 0 ("not at all") to 10 ("very strong").

Not at all									Very strong	
0	1	2	3	4	5	6	7	8	9	10
☐	☐	☐	☐	☐	☐	☐	☐	☐	☐	☐

[WHO5] The following statements relate to your well-being in the last two weeks. For each statement, please mark the section that you think best describes how you have felt in the past two weeks.

Over the last two weeks …

	All of the time	Most of the time	More than half of the time	Less than half of the time	Some of the time	At no time
[WHO_1] … I have felt cheerful and in good spirits.	☐	☐	☐	☐	☐	☐
[WHO_2] … I have felt calm and relaxed.	☐	☐	☐	☐	☐	☐
[WHO_3] … I have felt active and vigorous.	☐	☐	☐	☐	☐	☐
[WHO_4] … I woke up feeling fresh and rested.	☐	☐	☐	☐	☐	☐
[WHO_5] … my daily life has been filled with things that interest me.	☐	☐	☐	☐	☐	☐

[PSS] The following questions ask you about your feelings and thoughts during the last month. In each case, you will be asked to indicate by circling how often you felt or thought a certain way.

In the last month, how often …

	Never	Almost never	Some-times	Fairly often	Very often
[PSS1] … have you felt that you were unable to control the important things in your life?	☐	☐	☐	☐	☐
[PSS2] … have you felt confident about your ability to handle your personal problems?	☐	☐	☐	☐	☐
[PSS3] … have you felt that things were going your way?	☐	☐	☐	☐	☐
[PSS4] … have you found that you could not cope with all the things that you had to do?	☐	☐	☐	☐	☐

2: General information

The following information helps to evaluate the results of the HaBIDS surveys. We use this information to investigate whether differences between participants are caused by certain demographic factors, such as gender or age. We do not evaluate the data for your person, but for those groups to which you can, for example, be assigned according to your age group, your gender or your school leaving certificate.

[sex] What is your gender?
☐ Male
☐ Female

[geb_dat] In which year were you born?
enter your answer here

[geb_DE] Were you born in Germany?
☐ Yes
☐ No

[geb_Land] In which country were you born?
▼ geb_DE == “No”
enter your answer here

[Einreise] Since when do you live in Germany?
Please specify the year or age at the time of entry.
▼ geb_DE == “No”
Year: *enter your answer here*
Age: *enter your answer here*

[marital] What is your marital status?
"Married", "divorced" and "widowed" also refers to a registered life partnership.
☐ Single
☐ Married
☐ Divorced
☐ Widowed

3: Education and profession

[SES1] What is your highest general education?
- ☐ Pupil, attend a full-time school
- ☐ Left elementary school without school leaving certificate
- ☐ Elementary school (German “Hauptschule”)
- ☐ Secondary school / high school (German “Realschule”)
- ☐ Polytechnic high school of the GDR with completion of the 8th or 9th class
- ☐ Polytechnic high school of the GDR with completion of the 10th class
- ☐ “Fachhochschulreife”, completion of a subject-specific high school
- ☐ General or subject-specific university entrance qualification / “Abitur” (German “Gymnasium” / “EOS”)
- ☐ Another school leaving certificate, namely:

[SES1a] Which school leaving certificate do you strive for?
▼ SES1 == “Pupil”
- ☐ Elementary school (German “Hauptschule”)
- ☐ Secondary school / high school (German “Realschule”)
- ☐ “Fachhochschulreife”, completion of a subject-specific high school
- ☐ General or subject-specific university entrance qualification / “Abitur”
- ☐ Another school leaving certificate, namely:

[SES2] What is your highest level of qualification?
- ☐ Still in vocational training (vocational preparation year, trainee, intern, student)
- ☐ Pupil and attend a vocational-oriented or technical school or similar
- ☐ No vocational qualification and not in vocational training
- ☐ Completed apprenticeship
- ☐ Training at vocational school, commercial school, or middle service public administration
- ☐ Training at a GDR technical school
- ☐ Training at a technical school, vocational academy or technical academy
- ☐ Bachelor of a university (of applied sciences)
- ☐ Degree of a university of applied sciences (z. B. Diplom, Master)
- ☐ University degree (z. B. Diplom, Magister, Staatsexamen, Master)
- ☐ Doctorate
- ☐ Don’t know
- ☐ Another professional qualification, namely:

[SES3] What is the average monthly net income of your household as a whole?
This household includes all persons who live and work together here.
The average monthly net income of your household is the sum of wages, salaries, income from self-employed activities (average net operations, i.e. minus operating expenses and taxes), or pension. Please also include the income from public aid, income from renting and leasing, property, housing, child allowance and other income, and then deduct taxes and social insurance contributions.
- ☐ Less than 1250 Euros
- ☐ 1250 to less than 1750 Euros
- ☐ 1750 to less than 2250 Euros
- ☐ 2250 to less than 3000 Euros
- ☐ 3000 to less than 4000 Euros
- ☐ 4000 to less than 5000 Euros
- ☐ 5000 and more
- ☐ I don‘t want to answer
- ☐ Don’t know

4: Internet

[Internet] Do you have access to the Internet in your household?

☐ Yes
☐ No

[Gerät] What devices do you use to access the Internet?
(multiple choice possible)
▼ Internet == “Yes”

☐ Desktop-PC
☐ Laptop
☐ Tablet
☐ Smartphone
☐ None
☐ Others (please specify):

[Internetnutzung] How often do you personally use the Internet for private purposes?
▼ Internet == “Yes”

☐ Daily / almost daily
☐ About two or three times a week
☐ About once a week
☐ About two or three times a month
☐ About once a month
☐ Less than once a month
☐ I don’t use the Internet at home, but there is an access

5: Pets

[pets] Do you have pets?
- ☐ Yes
- ☐ No

[pet_art] What pets do you have?
(multiple choice possible)
▼ pets == “Yes”
- ☐ Dog(s)
- ☐ Cat(s)
- ☐ Rodent(s)
- ☐ Other (please specify):

C (mixed-mode) / D (online-only): Prevention measures against respiratory infections

The following questions are about how to protect yourself from respiratory infections.

1: Preventing respiratory infections

[VIa] Do you think that the following behaviors can prevent respiratory infections or minimize the risk of disease?

	Protects very well	Protects well	Partly partly	Doesn‘t protect well	Doesn‘t protect at all	Don’t know
[VI1] Thorough hand washing with soap	☐	☐	☐	☐	☐	☐
[VI2] Relaxation exercises	☐	☐	☐	☐	☐	☐
[VI3] Regular ventilation	☐	☐	☐	☐	☐	☐
[VI4] Ingestion of vitamin C (as a dietary supplement)	☐	☐	☐	☐	☐	☐
[VI5] Saunas	☐	☐	☐	☐	☐	☐
[VI6] Endurance sports	☐	☐	☐	☐	☐	☐
[VI7] Nasal irrigation with water	☐	☐	☐	☐	☐	☐
[VI8] Healthy diet, lots of fruits and vegetables	☐	☐	☐	☐	☐	☐
[VI9] Homeopathic remedies	☐	☐	☐	☐	☐	☐

[VI9_h] What homeopathic remedies can minimize the risk of disease?
▼ VIa_VI9 == “Protects (very) well”
enter your answer here

[VIb] Do you think that the following behaviors can prevent respiratory infections or minimize the risk of disease?

	Protects very well	Protects well	Partly partly	Doesn‘t protect well	Doesn‘t protect at all	Don’t know
[VI10] Movement in the fresh air	☐	☐	☐	☐	☐	☐
[VI11] Regular consumption of probiotic yoghurts	☐	☐	☐	☐	☐	☐
[VI12] Alternating showers or foot baths (alternately hot and cold)	☐	☐	☐	☐	☐	☐
[VI13] Don’t overheat housing	☐	☐	☐	☐	☐	☐
[VI14] Drink a lot (water, tea, ...)	☐	☐	☐	☐	☐	☐
[VI15] Sufficient sleep	☐	☐	☐	☐	☐	☐
[VI16] Avoid freezing and cold	☐	☐	☐	☐	☐	☐
[VI17] Avoid contact with patients (don’t shake hands, don’t drink from the same glass, etc.)	☐	☐	☐	☐	☐	☐

2: Measures

[VI_Ua] Which of the following are you implementing?

	Several times a day	Once daily	3 times or more per week, but not daily	1 to 2 times a week	Less than 1 time per week	Never
[VI_U1] Thorough hand washing with soap	☐	☐	☐	☐	☐	☐
[VI_U2] Relaxation exercises	☐	☐	☐	☐	☐	☐
[VI_U3] Regular ventilation	☐	☐	☐	☐	☐	☐
[VI_U4] Taking vitamin C (as a dietary supplement)	☐	☐	☐	☐	☐	☐
[VI_U5] Saunas	☐	☐	☐	☐	☐	☐
[VI_U6] Endurance sports	☐	☐	☐	☐	☐	☐
[VI_U7] Nasal irrigation with water	☐	☐	☐	☐	☐	☐
[VI_U8] Healthy diet, lots of fruits and vegetables	☐	☐	☐	☐	☐	☐
[VI_U9] Homeopathic remedies	☐	☐	☐	☐	☐	☐
[VI_U10] Movement in the fresh air	☐	☐	☐	☐	☐	☐
[VI_U11] Regular consumption of probiotic yoghurts	☐	☐	☐	☐	☐	☐
[VI_U12] Alternating showers or foot baths (alternately hot and cold)	☐	☐	☐	☐	☐	☐

[VI_Uc] Which of the following are you implementing?

	Always	Very often	Often	Sometimes	Never
[VI_U13] Don’t overheat housing	☐	☐	☐	☐	☐
[VI_U14] Drink a lot (water, tea, ...)	☐	☐	☐	☐	☐
[VI_U15] Sufficient sleep	☐	☐	☐	☐	☐
[VI_U16] Avoid freezing and cold	☐	☐	☐	☐	☐
[VI_U17] Avoid contact with patients (don’t shake hands, don’t drink from the same glass, etc.)	☐	☐	☐	☐	☐

3: Behavior during cold time

[VI_winter] Do you consciously behave differently in the cold / winter season to protect yourself from a cold?

☐ Yes

☐ No

4: Other measures

[VI_and] Do you take other measures that have not been mentioned so far to protect yourself from a disease?
Measure 1: *enter your answer here*
Measure 2: *enter your answer here*
Measure 3: *enter your answer here*

C (online-only): Determinants for participation in community-based cohorts on infectious diseases

We plan to conduct a scientific study in which the transmission and risks of gastrointestinal and respiratory diseases such as colds are to be investigated using new methods. The results are supposed to lead to the development of better protective measures. Thus, you will also benefit in the future.

To prepare for this study, we would like to know the circumstances under which you would be generally willing to participate in such a study. Your responses help us to design the study in a way that best suits the needs of future participants. Your opinion is important to us. If you complete this questionnaire, it does not mean that you must participate in the planned study or be invited to do so.

The following questions deal with the conditions under which you would participate in such a study.

1: Report on disease signs

[Nino1] If you should report daily on the onset of disease signs such as cough or diarrhea, would you be willing to do that for over 2 years?
☐ Yes, I would do it for 2 years
☐ Not at all
☐ I would do it for the following duration at maximum (please enter number of months):

[Nino2] If you should report weekly on the onset of disease signs such as cough or diarrhea, would you be willing to do that for over 2 years?
☐ Yes, I would do it for 2 years
☐ Not at all
☐ I would do it for the following duration at maximum (please enter number of months):

[Nino3] If you should report monthly on the onset of disease signs such as cough or diarrhea, would you be willing to do that for over 2 years?
☐ Yes, I would do it for 2 years
☐ Not at all
☐ I would do it for the following duration at maximum (please enter number of months):

2: Report on disease signs (continued)

[Nino4] How would you like to regularly report the symptoms like cough or diarrhea in the following way?

	Gladly	Partly, partly	Reluctantly
[1] Via a specific electronic mobile device intended only for this study and not linked to any devices or data outside the study	☐	☐	☐
[2] Via a study smartphone including flat rate, which I also use for telephone calls and may keep after the study	☐	☐	☐
[3] Via an app on my own smartphone	☐	☐	☐
[4] Via SMS on my own mobile phone	☐	☐	☐
[5] Via a web page of my own computer / laptop / tablet	☐	☐	☐
[6] Via emails from my own computer / laptop / tablet / smartphone	☐	☐	☐
[7] Via paper questionnaires, which I send by post free of charge	☐	☐	☐

3: Record location

[Nino5] Would you agree that such a study records your location?
☐ Yes
☐ No

[Nino5a] What kind of recording of your location would you agree with?
▼ Nino5 == “Yes”

	Yes, agreed	Partly, partly	No, not agreed
[1] If my identity is not identifiable, i.e. it remains completely anonymous	☐	☐	☐
[2] If the location data is not collected via my own mobile phone / smartphone and the identity of my location data is deleted after the link with the disease data	☐	☐	☐
[3] If the location data is captured by a separate device independent of my own or a study smartphone and the identity of my location data is deleted after linking to the disease data	☐	☐	☐
[4] If the linkage of the location data with my identity is deleted after the study has expired	☐	☐	☐

[Nino5a_other] If another condition, namely the following condition is satisfied:
▼ Nino5 == “Yes ”
enter your answer here

4: Biological samples

[Nino6] Would you be willing to take a smear from the nasal cavity in a respiratory disease with a cotton pad and send it to us?
The material is provided by us. Shipping is free of charge for you.

- ☐ Yes
- ☐ No
- ☐ Yes, but only on the condition that (please specify):

[Nino7] Would you be willing to send us a stool sample in the case of diarrhea, which you can take according to a manual?
The material is provided by us. Shipping is free of charge for you.

- ☐ Yes
- ☐ No
- ☐ Yes, but only on the condition that (please specify):

5: Data protection

[Nino8] Data protection and privacy are very important to us. What kind of data protection measures would you want to insist in?

	Yes	Neutral	No
[1] The entire data acquisition must have nothing to do with my mobile phone.	☐	☐	☐
[2] Symptoms such as cough or diarrhea can be transmitted via my mobile phone, but not my location.	☐	☐	☐
[3] I would like to be asked regularly to confirm whether I would like to continue the study.	☐	☐	☐
[4] In order not to have to reveal my identity (e.g. address), I am willing to provide material for stool samples anonymously at a pick-up point near me.	☐	☐	☐
[5] I want to disable the location function at any time.	☐	☐	☐
[6] The data from the study should not be used commercially.	☐	☐	☐
[7] I would participate in the study, independently of the specific data protection procedure, as long as this was supported by the responsible data protection officer.	☐	☐	☐
[8] I would not want to participate in any of the above circumstances.	☐	☐	☐

[Nino8_other] I would insist in another data protection measure not mentioned here, namely:
enter your answer here

6: Motivation

If the study were to be introduced in your neighborhood, how important would you consider the following aspects to motivate people to participate in such a scientific study?
Please estimate whether the measures below are more beneficial, unimportant, or more hindering to motivate people to participate.

[Nino9a] Support of the study by certain persons / institutions:

	More beneficial	Unimportant	More hindering
[1] Politicians and celebrities of the region	☐	☐	☐
[2] Physicians of the region	☐	☐	☐
[3] Churches and religious communities	☐	☐	☐
[4] Mayor / town council	☐	☐	☐
[5] Local societies and associations	☐	☐	☐
[6] Your employer	☐	☐	☐

[Nino9b] Offers for study participants:

	More beneficial	Unimportant	More hindering
[1] Regular feedback of own data compared with the average of the data of all other participants in the study. You then see whether you are, for example, more frequently ill than the average of the participants.	☐	☐	☐
[2] Regular feedback of the average data, without communication of own data	☐	☐	☐
[3] Providing individual health advice by e-mail e.g. to recommended vaccinations	☐	☐	☐
[4] The participants receive financial compensation.	☐	☐	☐
[5] The study smartphone may be used for private purposes and kept after to the study.	☐	☐	☐
[6] There are regular social events, where the participants can meet and inform about the study.	☐	☐	☐
[7] The participants themselves encourage family members / friends / work colleagues to take part in the study.	☐	☐	☐
[8] The knowledge that family members / friends / colleagues already participate in the study.	☐	☐	☐
[9] There is the possibility of submitting research questions themselves.	☐	☐	☐
[10] If the results of the study can contribute directly to reducing the incidence of infections in their own environment.	☐	☐	☐
[11] If the participants are recognizable as researchers of the project (for example, listed on a website on request, or, if desired, mentioned in scientific publications).	☐	☐	☐

7: Percentage of participation

[Nino10] We have mentioned above various ways to conduct the study. Please estimate, which percentage of the population in your community would participate in such a study, if…

	I estimate that ... percent of the population would participate
[1] ... the effort would be very limited (short duration (6 months), simple and rare inquiries (monthly), no sampling in case of respiratory disease or diarrhea):	Please specify an estimate between 0% and 100%.
[2] ... the effort would be relatively high (long duration (2 to 3 years), frequent inquiries (daily) with more than 3 samples per half-year):	Please specify an estimate between 0% and 100%.
[3] ... maximum anonymity would be ensured, i.e. no location recording:	Please specify an estimate between 0% and 100%.
[4] ... the research institute could assign my identity to the data but would not pass it on to other persons or institutions:	Please specify an estimate between 0% and 100%.

8: Acceptance of blood sampling

[Nino11] Some research questions can only be carried out by the examination of blood. Under which circumstances would you agree?

	Yes, agreed	Partly, partly	No, not agreed
[1] Only once at the beginning and at the end of the study	☐	☐	☐
[2] Only in the case of blood collection by a doctor of my trust	☐	☐	☐
[3] In the case of blood sampling in a study center by medical staff	☐	☐	☐
[4] By myself, by a special device, apply a bloodstripe from my fingertip to special paper and send it	☐	☐	☐
[5] In the case of blood sampling by medical staff who come to my home	☐	☐	☐

9: Study center

[Nino12] Some studies require detailed medical examinations and surveys in a study center. If the study center was nearby, would you be willing to participate in an investigation program lasting 6 hours?

☐ Yes
☐ Not at all
☐ I would do it for the following duration at maximum (please enter duration in hours):

[Nino12a] What day of the week and what time would you prefer to visit the study center?
You can specify several days. Please enter "0" if the weekday is not possible for you.
▼ Nino12 != “Not at all”

[1] Mondays	between ... o‘ clock	and ... o‘ clock
[2] Tuesdays	between ... o‘ clock	and ... o‘ clock
[3] Wednesdays	between ... o‘ clock	and ... o‘ clock
[4] Thursdays	between ... o‘ clock	and ... o‘ clock
[5] Fridays	between ... o‘ clock	and ... o‘ clock
[6] Saturdays	between ... o‘ clock	and ... o‘ clock

10: Household

[hh] How many people, including yourself, are living in your household?
A household includes all persons who live and work together here. Please also think of all children living in the household.
If you live in several households (e.g., weekend commuters), please refer to the household where you spend most of your time.

☐ I live alone in the household
☐ Number of people in my household (me included):

[hhkinder] Do minor children live in your household?
▼ hh != “I live alone in the household”

☐ Yes
☐ No

[hhage_pt] How old are the members of your household (including yourself)? Please start with the youngest member. For children under one year, please enter "0".
▼ hh != “I live alone in the household”

[1] Age (in years)
[2] Age (in years)
[3] Age (in years)
[4] Age (in years)
[5] Age (in years)
[6] Age (in years)
[7] Age (in years)
[8] Age (in years)
[9] Age (in years)
[10] Age (in years)

11: Participation of children

[Nino13a] Would you generally agree if the children in your household would participate in the study?
▼ hhkinder == “Yes”

☐ Yes
☐ No
☐ Don’t know

[Nino13b] Would you agree in particular if your child / children...
▼ Nino13a == “Yes” | Nino13a == “Don’t know”

	Yes	No
[1] ... would participate in the survey or you would report on the symptoms of your child / children?	☐	☐
[2] ... would send us a stool sample in case of diarrhea?	☐	☐
[3] ... would take a smear from the nasal cavity in a respiratory disease with a cotton pad and send it to us?	☐	☐
[4] ... would transfer their location to us?	☐	☐

12: Remarks

[Nino14a] What research questions do you find particularly interesting in the context of transmission paths?
enter your answer here

[Nino14b] What characteristics of such a study could be especially conducive to participation?
enter your answer here

[Nino14c] What characteristics of such a study would deter people from participating?
enter your answer here

[Nino14d] Would you like to tell us something else?
enter your answer here

D (mixed-mode): Social contact patterns (summer)

This questionnaire is about how many people you have contact with in one day.

Contact means that you have spoken with one person more than 10 words and were not more than 2 meters away from that person. Shaking hands or kissing is also counted as contact (i.e. whenever you touch a person's skin).

You can start the survey only once. You can now enter the contacts for yesterday, or wait until tomorrow and fill in the contacts retrospectively for today.

Before you complete the questionnaire, please refer to the pdf guide to see how you can enter the contacts. In order to make sure you have not forgotten a contact, we suggest that you try to go through the whole day in your mind. Your appointment book may be useful. Since it can be difficult not to mess with the contacts, we suggest that you first write down all contact persons on paper and then pass the information to the online questionnaire. A table for your notes is included in the pdf guide. You can also take notes during the day and enter your contacts in the evening.

1: Household

[hh_single] Do you live alone in your household?
If you live in several households (e.g. weekend commuters), please refer to the household where you spend most of your time.
☐ Yes
☐ No

[hh] How many people, including yourself, are living in your household?
A household includes all persons who live and work together here. Please also think of all children living in the household.
▼ hh_single == “No”
enter your answer here

[hhage_pt1] How old are the members of your household (including yourself)?
Please start with the youngest members. For children under one year, please enter "0".
▼hh_single == “No”
[1] Age (in years)
[2] Age (in years)
[3] Age (in years)
[4] Age (in years)
[5] Age (in years)
[6] Age (in years)
[7] Age (in years)
[8] Age (in years)
[9] Age (in years)
[10] Age (in years)

2: Regular contacts in work / education / school

"Contact" means, for example:
You spoke to a person and the distance was no more than two meters. Please don’t enter any contacts that have been made by telephone only.
or
You have touched the skin of another person (for example, shaking hands, kissing, contact sports ...).

[Kontakt] Do you regularly have a lot of contact with other people, in other words, more than 10 contacts with customers, pupils, students, colleagues, etc.?
☐ Yes
☐ No

[Kontakt_zu] I regularly have lots of contact with…
(multiple choice possible)
▼ Kontakt == “Yes”
☐ Customers
☐ Pupils
☐ Students
☐ Patients
☐ Colleagues
☐ Other (please specify):

[Kontakt_anz] With how many people (customers, students, patients, etc.) per day do you regularly have lots of contact?
Please include the persons who you only meet during work / training or at school. Please don’t include these persons in the following questions. There, please enter only the persons you have met (also) private.
▼ Kontakt == “Yes”
enter your answer here

3: Interpersonal contacts in leisure time

We would like to ask you to enter all the contacts which have taken place within one day in the following questionnaire. Look for a day, e.g. yesterday. Please refer to a total of 24 hours.

Start with the first person you were in contact with on the day you completed the questionnaire. If you had contact with other persons on the day, answer the question "Did you have contact with further persons on this day?" with "Yes" and click "Next" to add another person. If you have not contacted another person, please answer "No" and click "Next". You will be directed to the end of the survey.

[Datum] Please enter the date of the day for which you complete the questionnaire for your personal contacts.
enter your answer here

4: 1st contact person

"Contact" means, for example:
You spoke to a person and the distance was no more than two meters. Please don't enter any contacts that have been made by telephone only.
or
You have touched the skin of another person (for example, handing, kissing, contact sports ...).

[Alter1] Age
If you don't know the exact age, give a very narrow estimation of the age range (e.g. 40-45).
enter your answer here

[Sex_1] Sex
☐ Female
☐ Male

[Ort1] Place of contact
(multiple choice possible)
☐ At home
☐ At work
☐ Kindergarten / school / university
☐ Means of transport (train, car, bus…)
☐ Leisure time
☐ Other (please specify):

[Haut_1] Have you touched the skin of this contact person?
☐ Yes
☐ No

[Kontakt_1] How often do you usually have contact with this person?
☐ (Almost) every day
☐ A few times a week
☐ A few times a month
☐ A few times a year or less often
☐ The first time

[Zeit_1] Total time spent with the person
☐ Less than 5 minutes
☐ 5 to 15 minutes
☐ 15 minutes to 1 hour
☐ 1 to 4 hours
☐ 4 hours or more

[weiterePerson1] Did you have contact with further persons on this day?
☐ Yes
☐ No

5: 2nd contact person

…

[Participants could fill in up to 72 contacts]

75: 72nd contact person

76: Thank you for your participation

Thank you for completing the questionnaire. We now have a few questions to you about the survey itself and how you have coped with completing it. The answers help us to improve the survey and make it more comprehensible.

[verstaendlich] How do you assess the following statements?

	Strongly agree	Agree	Disagree	Strongly disagree
[1] Completing the survey was easy.	☐	☐	☐	☐
[2] The pdf guide was helpful.	☐	☐	☐	☐
[3] Completing the survey went quickly.	☐	☐	☐	☐
[4] It was difficult to keep track of which contact has already been registered during completing.	☐	☐	☐	☐

[Papier] Have you used the note tables from the pdf manual to keep track of your entries?

☐ Yes

☐ No

[Anmerkung] Do you have any further comments or suggestions for improvement?
enter your answer here

If you have entered all the contacts you had on your chosen day, you can now end the survey by clicking on "Submit".

E: Adult vaccinations

In Germany, an independent body of experts recommends which vaccinations are useful in which age. Most vaccinations are carried out in the early childhood in order to protect children from infections as early as possible. In this questionnaire, however, we are specifically interested in vaccinations in adulthood.

1: Vaccinations

[IM] Have you been vaccinated against the following infections in the last 10 years?

	Yes	No	Don’t know
[di] Diphtheria	☐	☐	☐
[te] Tetanus (wound stiff spasm)	☐	☐	☐
[pe] Pertussis (whooping cough)	☐	☐	☐
[pn] Pneumococcal infection	☐	☐	☐
[po] Poliomyelitis (infantile paralysis)	☐	☐	☐
[he] Hepatitis B	☐	☐	☐

[IM1] Are you planning to get vaccinated against one or more of the above-mentioned infections?

	Yes	No	Don’t know
[di] Diphtheria	☐	☐	☐
[te] Tetanus (wound stiff spasm)	☐	☐	☐
[pe] Pertussis (whooping cough)	☐	☐	☐
[pn] Pneumococcal infection	☐	☐	☐
[po] Poliomyelitis (infantile paralysis)	☐	☐	☐
[he] Hepatitis B	☐	☐	☐

2: Influenza

[IM2gr] How many times have you been vaccinated against influenza in the last 10 years?
☐ Not at all
☐ Once only
☐ 2 to 3 times
☐ 4 to 7 times
☐ Almost every year (8 to 9 times)
☐ Every year (10 times)
☐ Don’t know

[IM2gr_v] Are you planning to get vaccinated against influenza in the 2014/2015 season?
☐ Yes
☐ No
☐ Don’t know

[IM2gr_a] Does your employer provide the influenza vaccination for employees?
☐ Yes
☐ No
☐ I don’t have an employer
☐ Don’t know

[IM2h1n1] Have you been vaccinated against "swine flu" in autumn / winter 2009/2010?
☐ Yes
☐ No
☐ Don’t know

3: Vaccination boosts

[IM3ber] Have you talked with your general practioner in the past 12 months about vaccination boosts?

☐ No, because I was not at my general practioner in the last 12 months.
☐ No, we have not talked about it.
☐ Yes, I asked my general practioner.
☐ Yes, my general practioner has asked me about it.
☐ I don't have a general practioner.
☐ Don't know

[IM3ber2] Have you, within the last 12 months, been advised by your specialist physician about vaccinations? (multiple choice possible)

☐ No
☐ Yes, from medical doctor
☐ Yes, from pharmacist
☐ Yes, from a doctor's assistant
☐ Yes, from a midwife
☐ Yes, from the medical advice center
☐ Yes, from the Health Office
☐ Yes, from my health insurance
☐ Don't know

4: Vaccination card

[IM4pass] Do you have a vaccination card?

☐ Yes
☐ No
☐ Don’t know

[IM4pass_wo] Do you know where your vaccination card is currently?
▼ IM4pass == “Yes”

☐ Yes, exactly
☐ Yes, approximately
☐ No

5: Official recommendations

[IM5know] Do you know which adult vaccinations are currently recommended?

	True	Untrue	Don't know
[1] All adults over 18 years should be vaccinated every 10 years against diphtheria and tetanus.	☐	☐	☐
[2] All adults over 18 years should be vaccinated every 10 years against pertussis.	☐	☐	☐
[3] All adults, who are not protected against measles, should be vaccinated.	☐	☐	☐
[4] All adults over 18 years should be vaccinated against influenza.	☐	☐	☐
[5] All adults over 60 years should be vaccinated once against pneumococcal infection. (Pneumococci are bacteria that can cause severe infections such as pneumonia.)	☐	☐	☐
[6] All adults over 18 years, who were not vaccinated against poliomyelitis, should be vaccinated.	☐	☐	☐
[7] All dog owners should be vaccinated against rabies.	☐	☐	☐

6: Vaccinations for adults

[IM6att] We would like to ask you some questions about your opinion about vaccinations.

	Strongly agree	Agree	Disagree	Strongly disagree	Don’t know
[1] Vaccinations are effective means to protect against severe infectious diseases.	☐	☐	☐	☐	☐
[2] Vaccinations are getting better and safer.	☐	☐	☐	☐	☐
[3] I am concerned that my immune system could become weakened as a result of too many vaccinations.	☐	☐	☐	☐	☐
[4] I will not get vaccinations because of fear of injections.	☐	☐	☐	☐	☐
[5] I will not get vaccinations because of fear of side effects.	☐	☐	☐	☐	☐
[6] I will not get vaccinations because of fear of long-term consequences.	☐	☐	☐	☐	☐
[7] I am for mandatory vaccinations for all adults.	☐	☐	☐	☐	☐
[8] I am for mandatory vaccinations for some individuals such as health care workers.	☐	☐	☐	☐	☐

7: Attitude

[IM7att_gen] What is your general attitude towards vaccinations?
- ☐ Supportive
- ☐ Slightly supportive
- ☐ Slightly negative
- ☐ Negative
- ☐ Don't know

[IM7att_te] What is your attitude towards vaccination against tetanus?
- ☐ Supportive
- ☐ Slightly supportive
- ☐ Slightly negative
- ☐ Negative
- ☐ Don't know

[IM7att_gr] What is your attitude towards vaccination against influenza?
- ☐ Supportive
- ☐ Slightly supportive
- ☐ Slightly negative
- ☐ Negative
- ☐ Don't know

8: HPV

[IM8hpv_know] Have you ever heard of human papillomavirus (HPV) vaccination?
☐ Yes
☐ No
☐ Don’t know

[IM8hpv] Are you vaccinated against HPV?
▼ IM8hpv_know != “No”
☐ Yes
☐ No
☐ Don’t know

[IM8hpv_v] Are you planning to get vaccinated against HPV?
▼ IM8hpv_know != “No”
☐ Yes
☐ No
☐ Don’t know

9: HPV children

[IM9hpv] Do you have children between the ages of 12 and 17?
(multiple choice possible)
▼ IM8hpv_know != "No"
☐ Yes, a daughter / several daughters
☐ Yes, a son / several sons
☐ No

[IM9hpv_to_im] Is your daughter / at least one of your daughters vaccinated against HPV?
▼ IM9hpv_IM9hpv_to == "Yes"
☐ Yes
☐ No
☐ Don't know

[IM9hpv_to_v] If your daughter is / daughters are not vaccinated against HPV: Would you recommend her / them to vaccinate against HPV?
▼ IM9hpv_IM9hpv_to == "Yes"
☐ Yes
☐ No
☐ Don't know

[IM9hpv_so_im] Is your son / at least one of your sons vaccinated against HPV?
▼ IM9hpv_IM9hpv_so == "Yes"
☐ Yes
☐ No
☐ Don't know

[Im9hpv_so_v] If your son is / sons are not vaccinated against HPV: Would you recommend him / them to vaccinate against HPV?
▼ IM9hpv_IM9hpv_so == "Yes"
☐ Yes
☐ No
☐ Don't know

F: Ebola risk perception

In March 2014, the first cases of Ebola virus disease were notified in Guinea (West Africa). In the following months, the disease spread to Sierra Leone, Liberia, Nigeria, and Senegal. The WHO declared this outbreak as a Public Health Emergency of International Concern.

We are interested in your thoughts about this outbreak and if/how Ebola affects your daily life in Germany.

1: Ebola virus disease

[angst1] Are you worried about Ebola?
☐ Yes
☐ No

[angst2] How much are you worried about Ebola?
Please mark a number from 1=”not much” to 5=”very much”
▼ angst1 == “Yes”

Not much			Very much	
1	2	3	4	5
☐	☐	☐	☐	☐

[wissen1] How do you rate your personal knowledge about Ebola virus disease?
☐ Very poor
☐ Poor
☐ Moderate
☐ Good
☐ Very good

[wissen2] How do you rate the information that you get from the media about the situation in African countries affected by Ebola?
☐ Very poor
☐ Poor
☐ Moderate
☐ Good
☐ Very good

2: Probability of acquiring Ebola

[knowledge] How can Ebola be transmitted?

	Yes	No	Don’t know
By direct contact with bodily fluids of infected persons, either dead or living	☐	☐	☐
By direct contact with infected, but asymptomatic persons	☐	☐	☐
Through air, if infected people cough or sneeze	☐	☐	☐
Through material which has been heavily contaminated with bodily fluids of dead or living infected persons	☐	☐	☐
Through drinking water	☐	☐	☐
Through food produced in Germany	☐	☐	☐
By casual contact with someone already sick, such as sitting next to the person (without any direct contact of bodily fluids)	☐	☐	☐
By wild animals in Africa (monkeys, bats)	☐	☐	☐
By wild animals in Germany (rats, foxes)	☐	☐	☐
By insects in Africa (mosquitoes, tsetse flies)	☐	☐	☐
By insects in Germany (midges)	☐	☐	☐

3: Probability of acquiring Ebola (continued)

[risk] If you think of the recent worldwide situation about Ebola: Do you think that you have a personal risk of acquiring Ebola…

	Highly likely	Quite likely	Quite unlikely	Highly unlikely	Does not apply
… at work?	☐	☐	☐	☐	☐
... in public transport?	☐	☐	☐	☐	☐
... in public places (school, childcare …) or public events?	☐	☐	☐	☐	☐
… at an airport in Germany?	☐	☐	☐	☐	☐
… as a patient in a German hospital?	☐	☐	☐	☐	☐
… at a doctor's office in Germany?	☐	☐	☐	☐	☐
… during a travel to affected countries?	☐	☐	☐	☐	☐
… by food imported from Western African countries?	☐	☐	☐	☐	☐
... by other products originating in West Africa?	☐	☐	☐	☐	☐

4: Probability of acquiring Ebola (continued)

[pandemie] Are you worried that…

	Highly likely	Quite likely	Quite unlikely	Highly unlikely
… in the next three months people might arrive in Germany who are identified as infected persons after their entry?	☐	☐	☐	☐
… individual persons might be infected with the Ebola virus in Germany during the next six months?	☐	☐	☐	☐
… in the next six months Ebola could spread in the general population of Germany similar to how it is spreading currently in West Africa?	☐	☐	☐	☐

5: Personal behavior and prevention measures

[travel1] Imagine that you have booked a flight for the coming week to travel to any of the affected countries in West Africa. What would you do?

- ☐ Take the flight.
- ☐ Cancel the flight, but only if I am paid back my money.
- ☐ Cancel the flight even if 100% of the travel costs would be lost.
- ☐ Cancel the flight, but only to a maximum loss of …% of the travel costs.

[travel2] Imagine that you have booked a flight for the coming week to a non-affected country in Africa. What would you do?

- ☐ Take the flight.
- ☐ Cancel the flight, but only if I am paid back my money.
- ☐ Cancel the flight even if 100% of the travel costs would be lost.
- ☐ Cancel the flight, but only to a maximum loss of …% of the travel costs.

6: Personal behavior and prevention measures (continued)

[action_change] Did you change your behavior because of the Ebola outbreak in West Africa?

	Yes	No	Does not apply
I engaged in precautionary purchases.	☐	☐	☐
I avoid contact to African people from acquaintanceship.	☐	☐	☐
I avoid contact to African people in public places.	☐	☐	☐
I avoid going to public events (football, theater …).	☐	☐	☐
I engaged in precautionary purchases.	☐	☐	☐

7: Personal behavior and prevention measures (continued)

[action_hospital] Would you change your behavior if an Ebola patient was evacuated from Africa and brought to Germany for treatment in a near-by hospital?

	Yes	Rather yes	Rather no	No
I would avoid public events and crowded places.	☐	☐	☐	☐
I would avoid using public transport.	☐	☐	☐	☐
I would avoid physical contact with other people.	☐	☐	☐	☐
I would increase my hygiene behavior (e.g. wash my hands more often)	☐	☐	☐	☐
I would wear a face mask outside of my home.	☐	☐	☐	☐
I would not want to be admitted to the same hospital.	☐	☐	☐	☐
I would not visit friends admitted to the same hospital.	☐	☐	☐	☐

8: Personal behavior and prevention measures (continued)

[measure] Should the following measures be introduced to prevent the spread of Ebola to Europe?

	Not on any account	Not encouraged	Encouraged	Yes, absolutely
Provide information on Ebola to all travelers coming from affected areas and provide advice in case one develops signs and symptoms	☐	☐	☐	☐
Get personal information of all travelers coming from affected areas and control their health for three weeks long upon arrival	☐	☐	☐	☐
Forbid return transport for Germans who get infected during aid missions in West Africa	☐	☐	☐	☐
Forbid bringing Ebola patients for treatment to Germany	☐	☐	☐	☐
Measure temperature for all travelers coming from affected countries upon arrival at Europe with subsequent quarantine for those with high temperature	☐	☐	☐	☐
Measure temperature for all travelers coming from affected countries when they are about to leave Africa with subsequent quarantine for those with high temperature	☐	☐	☐	☐
Three weeks of mandatory quarantine for all volunteers returning from aid missions in West Africa	☐	☐	☐	☐
Entry restrictions for people from affected countries	☐	☐	☐	☐
Forbid travelling from Germany to affected countries in Africa	☐	☐	☐	☐
Compulsory vaccination against Ebola for all inhabitants of affected countries as soon as a vaccine is available	☐	☐	☐	☐

9: Personal commitment

[help1] Would you volunteer to fight Ebola in West Africa if your experience and knowledge were needed and if your personal situation and your health allowed so?

☐ Yes ☐ Unlikely
☐ Likely ☐ No
☐ Don’t know

[help2] What would be the main reason not to volunteer?

☐ I would be worried about getting infected.
☐ I think that the help would not be useful.
☐ I would be afraid to be overwhelmed by the situation on site.
☐ I would be worried that I might not return to Germany if I get infected.
☐ I would be worried about not being able to get back to Germany because of an entry restriction.
☐ I think every country should solve its problems by itself without depending on help from other countries.
☐ Other reason, namely:

[money1] Would you be willing to donate for the fight against Ebola in Africa?

☐ Yes ☐ Unlikely
☐ Likely ☐ No
☐ Don’t know

[money2] Which sum would you donate?
If you have already donated: How much have you donated?
▼ money1 != “No”

☐ Up to 10 Euros
☐ 11 to 20 Euros
☐ 21 to 50 Euros
☐ 51 to 100 Euros
☐ 101 to 200 Euros
☐ More than 200 Euros

[money3] Would you support a nonrecurring, compulsory, and income-related payment for the fight against Ebola in Africa?

☐ Yes ☐ Unlikely
☐ Likely ☐ No
☐ Don’t know

[money4] Which sum would you pay for the cause?
Please enter as a percentage of your monthly net income.
▼ money3 != “No”
enter your answer here

10: Vaccination

[vaccination1] If a vaccine against Ebola existed, would you opt for the vaccination even if you do not plan to visit affected countries in West Africa and do not have contact with Ebola patients ever?

☐ Yes
☐ No
☐ Don't know

[vaccination2] Would you still do so if the vaccine was associated with occasional mild side effects?
▼ vaccination1 != "No"

☐ Yes
☐ No
☐ Don't know

[vaccination3] Would you still do so if the vaccine was associated with rare and severe side effects?
▼ vaccination1 != "No"

☐ Yes
☐ No
☐ Don't know

[vaccination4] Should there be a compulsory vaccination against Ebola for the medical staff in Germany?

☐ Yes
☐ No
☐ Don't know

[vaccination5] Should there be a compulsory vaccination against Ebola for the general population in Germany if the number of Ebola cases in Germany increased?

☐ Yes
☐ No
☐ Don't know

[vaccination6] A vaccine must be tested for safety and efficacy. In which country should this be done?

	Yes	No	Don't know
In the country that develops the vaccine	☐	☐	☐
In the affected countries of West Africa	☐	☐	☐

11: Activities about Ebola

[info] Have you changed your media use since the Ebola outbreak has become public?

	Yes	No
I use the Internet more often/in addition to inform myself about Ebola.	☐	☐
I use television more often/in addition to inform myself about Ebola.	☐	☐
I use the radio more often/in addition to inform myself about Ebola.	☐	☐
I use print media more often/in addition to inform myself about Ebola.	☐	☐
I use information from public institutions (e.g. Robert Koch-Institute, World Health Organization) more often/in addition to inform myself about Ebola.	☐	☐

[activity] Did you do something among the following list of actions in connection to Ebola?
(multiple choice possible)

☐ Write a letter to the editor or a blog entry about Ebola in a paper or on the Internet
☐ Write a comment to an article in the Internet (e.g. Tagesschau.de, Spiegel online)
☐ Discussion in the circle of acquaintances
☐ Adhered to preventive measures at work
☐ Donations
☐ Participation in information events
☐ Organizing an information event
☐ Offer to participate in aid missions in Africa
☐ Offer to help in Germany

[contact] At your workplace, do you have contact to…?

	Yes	No
… patients or persons in need of care?	☐	☐
… more than 10 persons a day (e.g. students, customers, colleagues)?	☐	☐

G: Tick-borne infections

Some diseases are transmitted by contact with animals, e.g. through stitches of ticks or mosquitoes. This questionnaire is about your experience with ticks.

1: Devices used

First, however, we would like to ask you 3 general questions about which device you use to complete the HaBIDS questionnaires. We can use your answers to optimize the presentation of our questionnaires.

[momentan] Which device have you used most frequently to fill the questionnaires of the HaBIDS study?
☐ Desktop PC
☐ Laptop PC
☐ Tablet PC
☐ Smartphone
☐ Internet-enabled game console
☐ Internet-enabled TV (Smart TV)
☐ Other device, namely:

[Zoll] How big is the screen of the device taht you have used most often to complete the questionnaires of the HaBIDS study?
You can specify the size (screen diagonal) in inches or cm. A smartphone has e.g. often a size of about 5 inches, a laptop can be 12 to 17 inches in size.
cm: *enter your answer here*
inches: *enter your answer here*

[zufrieden] Are you satisfied with the presentation of the questionnaires?
Please refer to the device you have used most frequently to complete the questionnaires of the HaBIDS study.
☐ Yes
☐ No

[change] What can we improve on the presentation of the questionnaires?
▼ zufrieden == “No”
enter your answer here

2

We continue with questions about your experiences with ticks.

[ZE1beruf] Are you often (at least once a week) professionally in the woods or meadows?
☐ Yes
☐ No

[ZE2privat] How often are you privately in the woods or meadows?
☐ Every day
☐ Three times or more per week, but not daily
☐ Once or twice a week
☐ Less than once a week
☐ Never

3

[ZE3ges] Did you see a tick in summer 2014?
☐ Yes
☐ No
☐ Don’t know

[ZE3ges_NS] Was it in Lower Saxony?
▼ ZE3ges == “Yes”
☐ Yes
☐ No
☐ Don’t know

[ZE4gest] Have you ever been bitten by a tick in your life?
☐ Yes
☐ No
☐ Don’t know

[ZE5som] Were you bitten by a tick in summer 2014?
▼ ZE4gest == “Yes”
☐ Yes
☐ No
☐ Don’t know

[ZE5som_oft] How many times have you been bitten by a tick this summer?
▼ ZE5som == “Yes”
enter your answer here

[ZE5som_NS] Was it at least once in Lower Saxony?
▼ ZE5som == “Yes”
☐ Yes
☐ No
☐ Don’t know

4

[ZE6know] How do you assess the following statements?

	Applies	Rather apllies	Does rather not aplly	Does not apply	Don’t know
[1] Borreliosis is transmitted by ticks.	☐	☐	☐	☐	☐
[2] Borreliosis is a serious disease.	☐	☐	☐	☐	☐
[3] Children are particularly at risk of getting infected with borreliosis.	☐	☐	☐	☐	☐
[4] I am worried about getting infected with borreliosis.	☐	☐	☐	☐	☐
[5] The disease early summer meningoencephalitis (TBE) is transmitted by ticks.	☐	☐	☐	☐	☐
[6] TBE is a serious disease.	☐	☐	☐	☐	☐
[7] Children are particularly at risk of getting infected with TBE.	☐	☐	☐	☐	☐
[8] I am worried about getting infected with TBE.	☐	☐	☐	☐	☐

5

[ZE7bor] Have you ever been diagnosed with borreliosis by a doctor?
☐ No
☐ Yes, it was in (please enter year):

[ZE7bor_symp] What was the reason for the fact that you were diagnosed with borreliosis? (multiple choice possible)
▼ (ZE7bor == “Yes”)
☐ I was bitten by a tick and had it removed from the doctor.
☐ After a tick bite, a red spot spread out from the puncture point of the tick (erythema migrans).
☐ After a tick bite, I had joint pain.
☐ After a tick bite, I had a headache.
☐ After a tick bite, I had a fever.
☐ I went to the doctor because of joint pain (regardless of a tick bite).
☐ I went to the doctor because of headache (regardless of a tick bite).
☐ I went to the doctor because of fever (regardless of a tick bite).
☐ Other circumstance, namely:

[ZE8bor_di] Do you know someone who has been diagnosed with borreliosis?
☐ Yes
☐ No

6

The following questions refer to the tick vaccination.

[ZE9im] Have you ever heard of tick vaccination?
☐ Yes
☐ No

[ZE9im] How do you assess the following statements?

	Applies	Rather applies	Does rather not apply	Does not apply	Don't know
[bor] The tick vaccination protects against borreliosis.	☐	☐	☐	☐	☐
[FSME] The tick vaccination protects against early summer meningoencephalitis (TBE).	☐	☐	☐	☐	☐

[ZE9im_er] Have you ever received a tick vaccination?
☐ Yes
☐ No
☐ Don't know

7

[ZE10ver] Do you think that the following behaviors can reduce the risk of being bitten by a tick?

	Protects very well	Protects well	Doesn‘t protect well	Doesn‘t protect at all	Don’t know
[1] Avoid walks in the woods	☐	☐	☐	☐	☐
[2] Avoid walks on meadows	☐	☐	☐	☐	☐
[3] Wear long clothing when staying in the woods or on meadows	☐	☐	☐	☐	☐
[4] Wear ankle-high shoes when staying in the woods or meadows	☐	☐	☐	☐	☐
[5] Put pants in the socks	☐	☐	☐	☐	☐
[6] Application of anti-tick agents	☐	☐	☐	☐	☐
[7] After staying in the woods or meadows, search the body for ticks	☐	☐	☐	☐	☐

[ZE11bew] The following is about the right procedure when one is bitten by a tick. How do you assess the following statements?

	Very important	Rather important	Rather important	Unim-portant	Don’t know
[1] The tick should be removed immediately.	☐	☐	☐	☐	☐
[2] You should go to the doctor to have the tick removed.	☐	☐	☐	☐	☐
[3] Before removing, an alcoholic solution should be applied to the tick.	☐	☐	☐	☐	☐
[4] Before removing, an ointment, e.g. toothpaste, should be applied to the tick.	☐	☐	☐	☐	☐
[5] Before removing, oil should be applied to the tick.	☐	☐	☐	☐	☐
[6] You should pull the tick straight out.	☐	☐	☐	☐	☐
[7] You should turn the tick out.	☐	☐	☐	☐	☐

[ZE13nach] How do you proceed after a tick bite or how would you proceed?

	In any case	Rather yes	Rather no	No way	Don't know
[1] I remove the tick immediately.	☐	☐	☐	☐	☐
[2] I go to the doctor to get the tick removed.	☐	☐	☐	☐	☐
[3] Before removing, I apply an alcoholic solution to the tick.	☐	☐	☐	☐	☐
[4] Before removal, I apply an ointment, e.g. toothpaste, to the tick.	☐	☐	☐	☐	☐
[5] Before removing, I apply oil to the tick.	☐	☐	☐	☐	☐
[6] I pull the tick straight out.	☐	☐	☐	☐	☐
[7] I turn the tick out.	☐	☐	☐	☐	☐

[ZE12schutz] What do you personally do to protect yourself from ticks?

	Always	Often	Some-times	Never	Don't know
[1] I avoid going into the woods.	☐	☐	☐	☐	☐
[2] I avoid going on meadows.	☐	☐	☐	☐	☐
[3] I wear long clothes when I go into the woods or meadows.	☐	☐	☐	☐	☐
[4] I wear ankle-high shoes when I go into the woods or meadows.	☐	☐	☐	☐	☐
[5] I put my pants in my socks when I walk into the woods or meadows.	☐	☐	☐	☐	☐
[6] I use anti-tick agents.	☐	☐	☐	☐	☐
[7] After the stay in the woods or meadows, I search the body for ticks.	☐	☐	☐	☐	☐

H: Antibiotics

In this part of the questionnaire, we ask you about antibiotics. Antibiotics are drugs that can slow down or kill pathogens in their growth. Infections are caused by pathogens and are often associated with fever.
In Germany antibiotics must be prescribed by a doctor.

This survey contains 9 questions. At the end of the survey, you'll also find a free text field where you can make entries if you want to tell us something about antibiotics.

1: Questions about antibiotics

[Antibiotika] Please remember the last 12 months. How often have you been prescribed an antibiotic during this period?
☐ Not at all
☐ Once
☐ Two to three times
☐ Four times or more
☐ Don’t know

[Beschwerden] On what complaints did you get prescribed antibiotics the last time?
(multiple choice possible)
▼ Antibiotika >= “Once”
☐ Cough
☐ Sore throat
☐ Earache
☐ Burning when urinating
☐ Wound / soft tissue inflammation
☐ Don‘t know
☐ Others, namely:

[Antibiotikum_jemals] Have you ever taken antibiotics in your life?
▼ Antibiotika == “Not at all”
☐ Yes
☐ No
☐ Don’t know

2: Knowledge

[AN] How do you assess the following statements?

	Applies	Rather applies	Does rather not apply	Does not apply	Don’t know
[1] Antibiotics act against bacteria.	☐	☐	☐	☐	☐
[2] Antibiotics act against viruses.	☐	☐	☐	☐	☐
[3] Penicillin is an antibiotic.	☐	☐	☐	☐	☐
[4] Paracetamol is an antibiotic.	☐	☐	☐	☐	☐
[5] Ibuprofen is an antibiotic.	☐	☐	☐	☐	☐
[6] If an antibiotic is not taken as recommended by the doctor, the risk of pathogens becoming resistant to this antibiotic increases. That is, they are no longer killed by the antibiotic.	☐	☐	☐	☐	☐
[7] Those who are more likely to take antibiotics have a higher risk that the body becomes immune to antibiotics. That is, the antibiotics no longer work.	☐	☐	☐	☐	☐
[8] I have ever asked my general practioner for the prescription of an antibiotic because of a cold.	☐	☐	☐	☐	☐
[9] I have antibiotics at home and take them if necessary.	☐	☐	☐	☐	☐

[Art] I have the following antibiotic / antibiotics at home:
▼ AN_9 != “Does not apply”
Antibiotic 1: *enter your answer here*
Antibiotic 2: *enter your answer here*

3

[AN] How do you assess the following statements?
Please answer spontaneously.

	Applies	Rather applies	Does rather not apply	Does not apply	Don’t know
[10] I usually take antibiotics exactly as recommended by the doctor or pharmacist.	☐	☐	☐	☐	☐
[11] I stop taking an antibiotic as soon as I can get better.	☐	☐	☐	☐	☐
[12] When a relative becomes ill, I share my antibiotic with him / her.	☐	☐	☐	☐	☐
[13] I don’t use antibiotics.	☐	☐	☐	☐	☐
[14] The development of antibiotic resistance is my concern. (Antibiotics lose their effectiveness through resistance.)	☐	☐	☐	☐	☐

4

[Nebenwirkungen] During the last antibiotic use, I suffered from the following side effects:
(multiple choice possible)
▼ Antibiotikum_jemals != “0”
☐ None
☐ Allergic reaction
☐ Skin rash
☐ Diarrhea
☐ Nausea
☐ Other:

[Th_beendet] If you have stopped the therapy at an early stage, why?
(multiple choice possible)
▼ Antibiotikum_jemals != “No”
☐ Because I had forgotten it
☐ Because I had too much stress
☐ Because I had already felt better
☐ Because I had feared side effects
☐ Because side effects occurred
☐ Other reason, namely:

[AN] How do you assess the following statements?
Please answer spontaneously.
When answering the following questions, please refer to the last time you took antibiotics.
▼ Antibiotikum_jemals != “No”

	Applies	Rather applies	Does rather not apply	Does not apply	Don’t know
[15] The last time I was prescribed an antibiotic, I asked my doctor for the prescription of antibiotics.	☐	☐	☐	☐	☐
[16] I have taken the recommended number of pills per day.	☐	☐	☐	☐	☐
[17] I have taken the pills in the recommended time intervals.	☐	☐	☐	☐	☐
[18] I have adhered to the recommended duration of treatment (e.g. 7 days).	☐	☐	☐	☐	☐

5: Remarks

[AN_remark] Is there anything else you would like to tell us about this questionnaire?
enter your answer here

I: Social contact patterns (winter)

This questionnaire is about how many people you have contact with in one day.

Contact means that you have spoken with one person more than 10 words and were not more than 2 meters away from that person. Shaking hands or kissing is also counted (i.e., whenever you touch a person's skin). You can fill in the contacts for yesterday, or wait until tomorrow and fill in the contacts retrospectively for today.

If you have a lot of contact with other people, that is, more than 10 contacts with customers, pupils, students, colleagues, etc., you don't need to list these professional contacts individually.

To make sure that you don't forget any contact outside of work / education / school, we suggest that you try to keep your records in mind all day. Your appointment book may be useful. Since it can be difficult not to mess with the contacts, we suggest that you first write down all contact persons on paper and then pass the information. You can also take notes during the day and enter your contacts in the evening.

1: Regular contacts in work / education / school

"Contact" means, for example:
You spoke to a person and the distance was no more than two meters. Please don't enter any contacts that have been made by telephone only.
or
You have touched the skin of another person (for example, shaking hands, kissing, contact sports ...).

[Kontakt] Do you regularly have a lot of contact with other people, in other words, more than 10 contacts with customers, pupils, students, colleagues, etc.?
☐ Yes
☐ No

[Kontakt_zu] I regularly have lots of contact with…
(multiple choice possible)
▼ Kontakt == "Yes"
☐ Customers
☐ Pupils
☐ Students
☐ Patients
☐ Colleagues
☐ Other (please specify):

[Kontakt_anz] With how many people (customers, students, patients, etc.) per day do you regularly have lots of contact?
Please include the persons who you only meet during work / training or at school. Please don't include these persons in the following questions. There, please enter only the persons you have met (also) private.
▼ Kontakt == "Yes"
enter your answer here

2: How to register your contacts

On the next pages of the survey, you can enter your contacts into such a table:

	Age		Sex		Place of contact						Have you touched the skin of this contact person?		How often do you usually have contact with this person?					Total time spent with the person					
	Exact age	or age range	Female	Male	At home	At work	Kindergarten / school / university	Means of transport (train, car, bus…)	Leisure time	Other (please specify):	Yes	No	(Almost) every day	A few times a week	A few times a month	A few times a year or less often	The first time	Less than 5 minutes	5 to 15 minutes	15 minutes to 1 hour	1 to 4 hours	4 hours or more	How many people with these characteristics did you meet?
[1]			☐	☐	☐	☐	☐	☐	☐	☐	☐	☐	☐	☐	☐	☐	☐	☐	☐	☐	☐	☐	

If you don’t enter anything in the last column, we assume that you have only one person with these properties.

[weiter] Click "Next" to start entering your contacts.
If you don’t want to continue this survey, please let us know:
☐ I don’t want to continue the survey.

3: Interpersonal contacts in leisure time

We would like to ask you to enter all the contacts which have taken place within one day in the following questionnaire. Look out day, e.g. yesterday. Please refer to a total of 24 hours.

[Datum] Please specify the date of the day for which you complete the questionnaire for your personal contacts.
enter your answer here

4: Contacts 1 - 10

	Age		Sex		Place of contact						Have you touched the skin of this person?		How often do you usually have contact with this person?					Total time spent with the person					
	Exact age	or age range	Female	Male	At home	At work	Kindergarten / school / university	Means of transport (train, car, bus…)	Leisure time	Other (please specify):	Yes	No	(Almost) every day	A few times a week	A few times a month	A few times a year or less often	The first time	Less than 5 minutes	5 to 15 minutes	15 minutes to 1 hour	1 to 4 hours	4 hours or more	How many people with these characteristics did you meet?
[1]			☐	☐	☐	☐	☐	☐	☐	☐	☐	☐	☐	☐	☐	☐	☐	☐	☐	☐	☐	☐	
[2]			☐	☐	☐	☐	☐	☐	☐	☐	☐	☐	☐	☐	☐	☐	☐	☐	☐	☐	☐	☐	
[3]			☐	☐	☐	☐	☐	☐	☐	☐	☐	☐	☐	☐	☐	☐	☐	☐	☐	☐	☐	☐	
[4]			☐	☐	☐	☐	☐	☐	☐	☐	☐	☐	☐	☐	☐	☐	☐	☐	☐	☐	☐	☐	
[5]			☐	☐	☐	☐	☐	☐	☐	☐	☐	☐	☐	☐	☐	☐	☐	☐	☐	☐	☐	☐	
[6]			☐	☐	☐	☐	☐	☐	☐	☐	☐	☐	☐	☐	☐	☐	☐	☐	☐	☐	☐	☐	
[7]			☐	☐	☐	☐	☐	☐	☐	☐	☐	☐	☐	☐	☐	☐	☐	☐	☐	☐	☐	☐	
[8]			☐	☐	☐	☐	☐	☐	☐	☐	☐	☐	☐	☐	☐	☐	☐	☐	☐	☐	☐	☐	
[9]			☐	☐	☐	☐	☐	☐	☐	☐	☐	☐	☐	☐	☐	☐	☐	☐	☐	☐	☐	☐	
[10]			☐	☐	☐	☐	☐	☐	☐	☐	☐	☐	☐	☐	☐	☐	☐	☐	☐	☐	☐	☐	

[weiter_10] Would you like to add more contacts?

☐ Yes

☐ No

5: Contacts 11 - 20

…

[Participants could fill in up to 70 contacts]

10: Contacts 61 - 70

11: Thank you for your participation

[verstaendlich] Thank you for completing the questionnaire. We now have a few questions to you about the survey itself and how you have coped with completing it. The answers help us to improve the survey and make it more comprehensible. How do you assess the following statements?

	Strongly agree	Agree	Disagree	Strongly disagree
[1] Completing the survey was easy.	☐	☐	☐	☐
[2] Completing the survey went quickly.	☐	☐	☐	☐
[3] It was difficult to keep track of which contact has already been registered during the completion process.	☐	☐	☐	☐

[Anmerkung] Would you like to tell us anything else?
enter your answer here

J: Frequency of infections during the winter season & influenza vaccination (season 2014/15)

In this questionnaire, we are interested in whether you had an infection of the respiratory tract in the past months and whether you were vaccinated against influenza in the autumn / winter season 2014/15.

1: Signs and symptoms

[IN1_2015] How often have you had an infection of the airways (for example, cold, flu, or otitis media) in the past 12 months?
☐ Not at all
☐ Once
☐ Twice
☐ Three to four times
☐ More than four times
☐ Don't know

[GR_1] Since September 2014, have you had an infection with high fever (body temperature above 38° C) lasting several days?
☐ Yes
☐ No
☐ I'm not sure

[GR_2] How many days did you have malaise?
▼ GR_1 != "No"
enter your answer here

[GR_3] How many days did you have high fever (body temperature above 38° C)?
▼ GR_1 != "No"
enter your answer here

[GR_4] Did you have other symptoms besides fever?
▼ GR_1 != "No"

	Yes	No	Don't know
[1] Throat pain	☐	☐	☐
[2] Dry cough	☐	☐	☐
[3] Muscle pain	☐	☐	☐
[4] Joint pain	☐	☐	☐
[5] Back pain	☐	☐	☐
[6] Headaches	☐	☐	☐
[7] Feeling of weakness	☐	☐	☐

[GR_4_8] Other symptoms:
▼ GR_1 != "No"
enter your answer here

2: Seeking medical advice

[GR_5] Did you see a doctor because of this infection?
▼ GR_1 != “No”
☐ Yes
☐ No

[GR_6] Did the doctor take a sample to diagnose a flu?
▼ (GR_5 == “Yes”)
☐ Yes
☐ No

[GR_7] Did the doctor diagnose a real flu or other disease, e.g. pneumonia, otitis media, or similar?
▼ (GR_1 != “No”) & (GR_5 == “Yes”)
☐ The doctor has not made any diagnosis.
☐ The doctor has diagnosed a real flu.
☐ The doctor has diagnosed another illness, namely:

3: Characteristics of the disease

[GR_8] How many days were you so ill that you had to stay in bed or could not pursue your normal everyday life (housework, hobbies etc.)?
▼ GR_1 != “No”
enter your answer here

[GR_9] How many days did you report ill and could not pursue your profession?
▼ GR_1 != “No”
☐ Does not apply to me
☐ Days:

[GR_10] How many of these {GR_9} days were you on ill leave?
▼ (GR_9 > “0”)
☐ Does not apply to me
☐ Days:

[GR_11] Do you think that this disease might have been the real flu?
☐ Yes
☐ No
☐ I’m not sure

[GR_11_comment_1] Why do you think it was a real flu?
▼ GR_11 == “Yes”
enter your answer here

[GR_11_comment_0] Why do you think it was not a real flu?
▼ GR_11 == “No”
enter your answer here

4: Household

[P2_hh_single] Do you live alone in your household?
If you live in several households (e.g. weekend commuters), please refer to the household where you spend most of your time.

☐ Yes
☐ No

[P2_hh_p] How many people, including yourself, are living in your household?
A household includes all persons who live and work together here. Please also think of all children living in the household.
▼ P2_hh_single == “No”
enter your answer here

[P2_hh_age1] How old are the member of your household (including yourself)?
Please start with the youngest member. For children under one year, please enter "0".
▼ P2_hh_single == “No”
[1] Age (in years)
[2] Age (in years)
[3] Age (in years)
[4] Age (in years)
[5] Age (in years)
[6] Age (in years)
[7] Age (in years)
[8] Age (in years)
[9] Age (in years)
[10] Age (in years)

[GR_12] Are there among those household members people who are particularly vulnerable to flu, e.g. elderly or chronically ill persons?
▼ P2_hh_single == “No”

☐ No
☐ I am not sure
☐ Yes, number of persons:

5: Household II

[GR_13] Since September 2014, did someone from your household (not counting you) have an disase with high fever (body temperature above 38° C) lasting several days?
▼ P2_hh_single == “No”
☐ Yes
☐ No
☐ I’m not sure

[GR_14] How many persons from your household (not counting you) have had an disease with high fever (body temperature above 38° C) lasting several days?
▼ GR_13 == “Yes”
enter your answer here

[GR_15] Do you think that this disease might have been the real flu?
▼ GR_13 == “Yes”
☐ Yes
☐ No
☐ I’m not sure

[GR_15_comment_1] Why do you think it was a real flu?
▼ GR_15 == “Yes”
enter your answer here

[GR_15_comment_0] Why do you think it was not a real flu?
▼ GR_15 == “No”
enter your answer here

[GR_16] If you think of your infection with high fever, were the other ill person(s) in your household ill before, at the same time or after yourself?
With this question we would like to find out whether there was any contagion within your household. Please tick all that apply.
(multiple choice possible)
▼ (GR_1 == “Yes”) & (GR_13 == “Yes”)
☐ In my household, one or more people were ill within two weeks before myself.
☐ In my household, one or more people were ill within two weeks after myself.
☐ In my household, one or more people were ill with me at the same time.
☐ In my household, one or more people were ill at another time, that is, not within 2 weeks before or after my own illness.
☐ I don’t know when the other person(s) in my household was ill.

6: Influenza vaccination

[GR_17] Have you been vaccinated against influenza since August 2014?
☐ Yes
☐ No
☐ I'm not sure

[GR_18] When did you get vaccinated against flu?
▼ GR_17 == "Yes"
enter your answer here

[GR_19] Where was the influenza vaccination performed?
▼ GR_17 == "Yes"
☐ General practioner
☐ Company doctor
☐ Gynecologist
☐ Pediatrician
☐ Somewhere else

[GR_20] Was this influenza vaccination recorded in your vaccination card?
▼ GR_17 == "Yes"
☐ I don't have a vaccination card.
☐ Yes
☐ No
☐ Don't know

[GR_21] Did you have to pay for the influenza vaccination?
▼ GR_17 == "Yes"
☐ Yes
☐ No
☐ Don't know

[GR_21_Zahlung] How much did you have to pay?
▼ GR_21 == "Yes"
enter your answer here

[GR_22] Would you have been vaccinated against influenza if you had to pay for the vaccination? (The vaccination costs about 20 euros.)
▼ GR_21 == "No"
☐ Yes
☐ No
☐ Don't know

[GR_23] After the influenza vaccination, did you have any complaints that you have associated with vaccination?
▼ GR_17 == "Yes"
☐ No
☐ Yes (please specify):

7: Influenza vaccination II

[GR_24] Did anyone give you the advice or recommendation to get vaccinated against influenza?
☐ Yes
☐ No
☐ Don’t know

[GR_25] Who gave you the advice or recommendation to get vaccinated against flu?
(multiple choice possible)
▼ GR_24 == “Yes”
☐ General practioner
☐ Gynecologist
☐ Pediatrician
☐ Family
☐ Someone else, namely:

[GR_26] Was the fact that someone gave you the advice or recommendation to get vaccinated against influenza very important for your decision to get vaccinated?
▼ (GR_17 == “Yes”) & (GR_24 == “Yes”)
☐ Yes
☐ No

[GR_27] Has your company / employer recommended the vaccination, e.g. email, notice on the bulletin board or similar?
☐ Yes
☐ No
☐ Does not apply to me

[GR_28] Was the fact that your company / employer recommended the vaccination very important for your decision to get vaccinated?
▼ (GR_17 == “Yes”) & (GR_27 == “Yes”)
☐ Yes
☐ No

8: Influenza vaccination III

[GR_29] Do you work in the medical field and have patient contact?
☐ Yes
☐ No

[GR_30] Was the fact that you have patient contact very important for your decision to get vaccinated?
▼ (GR_17 == “Yes”) & (GR_29 == “Yes”)
☐ Yes
☐ No

[GR_31] Was the fact that your household is home to a person for whom a flu would be particularly harmful, very important for your decision to get vaccinated?
▼ (GR_17 == “Yes”) & (GR_12 == “Yes”)
☐ Yes
☐ No

9: Reason for influenza vaccination

[GR_32] Did you have the impression that in the media the influenza wave was shown as particularly strong in winter 2014/2015?

☐ Yes

☐ No

[GR_33] Was the fact that in the media the flu wave in the winter 2014/2015 was shown as particularly strong very important for your decision to get vaccinated?

▼ (GR_17 == “Yes”) & (GR_32 == “Yes”)

☐ Yes

☐ No

[GR_34] Was the fact that you did not want to get influenza yourself very important for your decision to get vaccinated?

▼ GR_17 == “Yes”

☐ Yes

☐ No

[GR_35] Please summarize briefly why you have decided to get vaccinated:

▼ GR_17 == “Yes”

enter your answer here

10: Statements about influenza vaccination

How do you assess the following two statements?

[GR_36] The information about the influenza vaccination, which I receive through the media, allows me to make a decision on whether to vaccinate myself or not.

- ☐ Applies
- ☐ Rather applies
- ☐ Does rather not apply
- ☐ Does not apply
- ☐ I have not received any such information through the media.

[GR_37] The information about the influenza vaccination, which I receive through the media, is clear to me.

- ☐ Applies
- ☐ Rather applies
- ☐ Does rather not apply
- ☐ Does not apply
- ☐ I have not received any such information through the media.

11: Recommendation influenza vaccination

[GR_38] For which groups of persons is the influenza vaccination recommended by the Standing Immunization Commission (STIKO) at the Robert Koch Institute?

	Yes	No	Don’t know
[1] For the elderly over 60 years.	☐	☐	☐
[2] For all adults over 18 years.	☐	☐	☐
[3] For persons with increased health risk due to chronic condition.	☐	☐	☐
[4] For children who visit a children's day-care center.	☐	☐	☐
[5] For persons who work in the medical field and have contact with the patient.	☐	☐	☐
[6] For women who are pregnant during the winter half year.	☐	☐	☐

12: Outlook influenza vaccination

[GR_39_f] Do you belong to one of the following groups?
(multiple choice possible)

☐ I am over 60 years old.
☐ I have a chronic condition.
☐ I have a child / children visiting a children's day.
☐ I gave birth to a child between September 2014 and March 2015.
☐ I am currently pregnant.
☐ I don’t belong to one of these groups.

[GR_40] Are you planning to get vaccinated against influenza in the autumn / winter season 2015/16?

☐ Yes
☐ No
☐ Not decided yet

[GR_41] Have your experiences with the influenza vaccination in the 2014/15 season affected your decision for the autumn / winter season 2015/16?
▼ GR_17 == “Yes”

☐ Yes
☐ No
☐ Don’t know

[GR_42] How did your experiences with the influenza vaccination in the 2014/15 season influence your decision for the autumn / winter season 2015/16?
▼ GR_41 == “Yes”
enter your answer here

[GR_remark] Is there anything else you would like to tell us about this questionnaire?
enter your answer here

K: Sexual contact patterns & medical history

The risk of getting an infection may be related to whether or not you are suffering from another condition. In the following, we are therefore interested in diseases which are not infections. These include e.g. sugar disease, heart attack or allergies.

1: Information about yourself and general health

For the analysis of this survey, it is very important that we analyse the questions separately by gender and age group. The frequency and severity of many diseases differs depending on gender or age.
Unfortunately, not all participants have entered gender and age. We would therefore ask you to enter these two details here (regardless of whether you have already made this information).

[sex_ON11] What is your gender?

☐ Female
☐ Male

[geb_dat_ON11] In which year were you born?
enter your answer here

[health_2015] How would you describe your health condition in general?

☐ Excellent
☐ Very good
☐ Good
☐ Fair
☐ Poor

[QoL] How satisfied are you with your quality of life?

☐ Very satisfied
☐ Satisfied
☐ Rather satisfied
☐ Rather dissatisfied
☐ Dissatisfied

2: Known dieases

[ANAM_1] Have you ever been diagnosed with a disease by a doctor (infections excluded)?
☐ Yes
☐ No
☐ Don’t know

[ANAM_2] Have you ever been in a hospital because of an illness (infections excluded)?
☐ Yes
☐ No
☐ Don’t know

[ANAM_3] Do you currently take any medication for a disease (infections excluded)?
☐ Yes
☐ No
☐ Don’t know

[ANAM_4] What are the diseases?
▼ (ANAM_1 == “Yes”) OR (ANAM_2 == “Yes”) OR (ANAM_3 == “Yes”)

	Yes	No	Don’t know
[1] Cardiovascular disease<br>e.g. stroke, heart attack, hypertension, cardiac arrhythmia, circulatory problems in the legs, cardiac insufficiency or constriction of the coronary vessels	☐	☐	☐
[2] Cancer	☐	☐	☐
[3] Metabolic disease<br>e.g. sugar disease, increased blood lipid, gout or thyroid disease	☐	☐	☐
[4] Muscle-skeletal disease<br>e.g. osteoporosis, osteoarthritis or back pain for 3 months or longer	☐	☐	☐
[5] Pulmonary disease<br>e.g. chronic bronchitis or asthma	☐	☐	☐
[6] Allergy	☐	☐	☐
[7] Gastrointestinal or liver disease<br>e.g. heartburn, stomach ulcer, Crohn's disease, bile stones or liver cirrhosis	☐	☐	☐
[8] Skin disease<br>e.g. neurodermatitis or psoriasis	☐	☐	☐
[9] Kidney disease	☐	☐	☐
[10] Neurological disease<br>e.g. migraine, epilepsy, multiple sclerosis, or Parkinson's disease	☐	☐	☐
[11] Psychiatric disease<br>e.g. depression or anxiety disease	☐	☐	☐
[12] Other (not listed) disease	☐	☐	☐

[ANAM_4_12_text] What other (not listed) disease is it?
▼ ANAM_4_12 == “Yes”
enter your answer here

3: Cardiovascular diseases

[ANAM_5] Which cardiovascular disease is / was it?
(multiple choice possible)
▼ ANAM_4_1 == “Yes”

☐ Stroke
☐ Heart attack
☐ High blood pressure
☐ Arrhythmia
☐ Circulation disease in the legs
☐ Cardiac insufficiency
☐ Narrowing of the coronary vessels or angina pectoris
☐ Others, namely:

[ANAM_6] When was the stroke? If you had multiple strokes, please refer to the first stroke only.
Please indicate your age at the time of the first diagnosis or the year of the first diagnosis, whichever you remember better.
▼ (ANAM_4_1 == “Yes”) AND (ANAM_5_1 == “Yes”)
Age: *enter your answer here*
Year: *enter your answer here*

[ANAM_7] Are you currently in medical treatment because of a stroke?
▼ (ANAM_4_1 == “Yes”) AND (ANAM_5_1 == “Yes”)

☐ Yes
☐ No

[ANAM_8] When was the heart attack? If you had multiple heart attacks, please refer to the first heart attack only.
Please indicate your age at the time of the first diagnosis or the year of the first diagnosis, whichever you remember better.
▼ (ANAM_4_1 == “Yes”) AND (ANAM_5_2 == “Yes”)
Age: *enter your answer here*
Year: *enter your answer here*

[ANAM_9] Are you currently in medical treatment because of heart attack?
▼ (ANAM_4_1 == “Yes”) AND (ANAM_5_2 == “Yes”)

☐ Yes
☐ No

[ANAM_10] When did hypertension occur for the first time?
Please indicate your age at the time of the first diagnosis or the year of the first diagnosis, whichever you remember better.
▼ (ANAM_4_1 == “Yes”) AND (ANAM_5_3 == “Yes”)
Age: *enter your answer here*
Year: *enter your answer here*

[ANAM_11] Are you currently in medical treatment because of hypertension?
▼ (ANAM_4_1 == “Yes”) AND (ANAM_5_3 == “Yes”)

☐ Yes
☐ No

[ANAM_12] When did arrhythmias occur for the first time?
Please indicate your age at the time of the first diagnosis or the year of the first diagnosis, whichever you remember better.
▼ (ANAM_4_1 == “Yes”) AND (ANAM_5_4 == “Yes”)
Age: *enter your answer here*
Year: *enter your answer here*

[ANAM_13] Are you currently in medical treatment because of arrhythmias?
▼ (ANAM_4_1 == “Yes”) AND (ANAM_5_4 == “Yes”)
☐ Yes
☐ No

[ANAM_14] When did circulatory problmes in the legs occur for the first time?
Please indicate your age at the time of the first diagnosis or the year of the first diagnosis, whichever you remember better.
▼ (ANAM_4_1 == “Yes”) AND (ANAM_5_5 == “Yes”)
Age: *enter your answer here*
Year: *enter your answer here*

[ANAM_15] Are you currently in medical treatment because of circulatory problems in the legs?
▼ (ANAM_4_1 == “Yes”) AND (ANAM_5_5 == “Yes”)
☐ Yes
☐ No

[ANAM_16] When did cardiac insufficiency occur for the first time?
Please indicate your age at the time of the first diagnosis or the year of the first diagnosis, whichever you remember better.
▼ (ANAM_4_1 == “Yes”) AND (ANAM_5_6 == “Yes”)
Age: *enter your answer here*
Year: *enter your answer here*

[ANAM_17] Are you currently in medical treatment because of cardiac insufficiency?
▼ (ANAM_4_1 == “Yes”) AND (ANAM_5_6 == “Yes”)
☐ Yes
☐ No

[ANAM_18] When did angina pectoris occur for the first time?
Please indicate your age at the time of the first diagnosis or the year of the first diagnosis, whichever you remember better.
▼ (ANAM_4_1 == “Yes”) AND (ANAM_5_7 == “Yes”)
Age: *enter your answer here*
Year: *enter your answer here*

[ANAM_19] Are you currently in medical treatment because of angina pectoris?
▼ (ANAM_4_1 == “Yes”) AND (ANAM_5_7 == “Yes”)
☐ Yes
☐ No

[ANAM_20] When did {ANAM_5_other} occur for the first time?
Please indicate your age at the time of the first diagnosis or the year of the first diagnosis, whichever you remember better.
▼ (ANAM_4_1 == “Yes”) AND (! (is_empty(ANAM_5_other))
Age: *enter your answer here*
Year: *enter your answer here*

[ANAM_21] Are you currently in medical treatment because of {ANAM_5_other}?
▼ (ANAM_4_1 == “Yes”) AND (! (is_empty(ANAM_5_other))
☐ Yes
☐ No

4: Cancer

[ANAM_22_f] Which cancer is / was it?
(multiple choice possible)
▼ (ANAM_4_2 == “Yes”) AND (sex_ON11 == “Female”)

☐ Lung
☐ Intestine
☐ Mammary gland
☐ Uterine body (endometrium)
☐ Cervix
☐ Ovaries
☐ Bladder
☐ Malignant melanoma of the skin (black skin cancer)
☐ Pancreas
☐ Stomach
☐ Kidney and urinary tract
☐ Mouth and throat
☐ Non-Hodgkin lymphoma
☐ Hodgkin's disease
☐ Leukemia
☐ Liver
☐ Esophagus
☐ Brain
☐ Thyroid
☐ Bone
☐ Larynx
☐ Gallbladder
☐ Others, namely:

[ANAM_22_m] Which cancer is / was it?
(multiple choice possible)
▼ (ANAM_4_2 == “Yes”) AND (sex_ON11 == “Male”)

☐ Lung
☐ Intestine
☐ Prostate
☐ Testicle
☐ Mammary gland
☐ Bladder
☐ Malignant melanoma of the skin (black skin cancer)
☐ Pancreas
☐ Stomach
☐ Kidney and urinary tract
☐ Mouth and throat
☐ Non-Hodgkin lymphoma
☐ Hodgkin's disease
☐ Leukemia
☐ Liver
☐ Esophagus
☐ Brain
☐ Thyroid
☐ Bone
☐ Larynx
☐ Gallbladder
☐ Others, namely:

[ANAM_23] When did cancer of the lung occur for the first time?
Please indicate your age at the time of the first diagnosis or the year of the first diagnosis, whichever you remember better.
▼ (ANAM_4_2 == “Yes”) AND (ANAM_22_f_1 == “Yes” OR ANAM_22_m_1 == “Yes”)
Age: *enter your answer here*
Year: *enter your answer here*

[ANAM_24] Are you currently in medical treatment because of cancer of the lung?
▼ (ANAM_4_2 == “Yes”) AND (ANAM_22_f_1 == “Yes” OR ANAM_22_m_1 == “Yes”)
☐ Yes
☐ No

[ANAM_25] When did cancer of the intestine occur for the first time?
Please indicate your age at the time of the first diagnosis or the year of the first diagnosis, whichever you remember better.
▼ (ANAM_4_2 == “Yes”) AND (ANAM_22_f_2 == “Yes” OR ANAM_22_m_2 == “Yes”)
Age: *enter your answer here*
Year: *enter your answer here*

[ANAM_26] Are you currently in medical treatment because of cancer of the intestine?
▼ (ANAM_4_2 == “Yes”) AND (ANAM_22_f_2 == “Yes” OR ANAM_22_m_2 == “Yes”)
☐ Yes
☐ No

[ANAM_27] When did cancer of the prostate occur for the first time?
Please indicate your age at the time of the first diagnosis or the year of the first diagnosis, whichever you remember better.
▼ (ANAM_4_2 == “Yes”) AND (ANAM_22_m_3 == “Yes”)
Age: *enter your answer here*
Year: *enter your answer here*

[ANAM_28] Are you currently in medical treatment because of cancer of the prostate?
▼ (ANAM_4_2 == “Yes”) AND (ANAM_22_m_3 == “Yes”)
☐ Yes
☐ No

[ANAM_29] When did cancer of the testicle occur for the first time?
Please indicate your age at the time of the first diagnosis or the year of the first diagnosis, whichever you remember better.
▼ (ANAM_4_2 == “Yes”) AND (ANAM_22_m_4 == “Yes”)
Age: *enter your answer here*
Year: *enter your answer here*

[ANAM_30] Are you currently in medical treatment because of cancer of the testicle?
▼ (ANAM_4_2 == “Yes”) AND (ANAM_22_m_4 == “Yes”)
☐ Yes
☐ No

[ANAM_31] When did cancer of the mammary gland occur for the first time?
Please indicate your age at the time of the first diagnosis or the year of the first diagnosis, whichever you remember better.
▼ (ANAM_4_2 == “Yes”) AND (ANAM_22_f_5 == “Yes” OR ANAM_22_m_5 == “Yes”)
Age: *enter your answer here*
Year: *enter your answer here*

[ANAM_32] Are you currently in medical treatment because of cancer of the mammary gland?
▼ (ANAM_4_2 == “Yes”) AND (ANAM_22_f_5 == “Yes” OR ANAM_22_m_5 == “Yes”)
☐ Yes
☐ No

[ANAM_33] When did cancer of the uterine body (endometrium) occur for the first time?
Please indicate your age at the time of the first diagnosis or the year of the first diagnosis, whichever you remember better.
▼ (ANAM_4_2 == “Yes”) AND (ANAM_22_f_6 == “Yes”)
Age: *enter your answer here*
Year: *enter your answer here*

[ANAM_34] Are you currently in medical treatment because of cancer of the uterine body (endometrium)?
▼ (ANAM_4_2 == “Yes”) AND (ANAM_22_f_6 == “Yes”)
☐ Yes
☐ No

[ANAM_35] When did cancer of the cervix occur for the first time?
Please indicate your age at the time of the first diagnosis or the year of the first diagnosis, whichever you remember better.
▼ (ANAM_4_2 == “Yes”) AND (ANAM_22_f_7 == “Yes”)
Age: *enter your answer here*
Year: *enter your answer here*

[ANAM_36] Are you currently in medical treatment because of cancer of the cervix?
▼ (ANAM_4_2 == “Yes”) AND (ANAM_22_f_7 == “Yes”)
☐ Yes
☐ No

[ANAM_37] When did cancer of the ovaries occur for the first time?
Please indicate your age at the time of the first diagnosis or the year of the first diagnosis, whichever you remember better.
▼ (ANAM_4_2 == “Yes”) AND (ANAM_22_f_8 == “Yes”)
Age: *enter your answer here*
Year: *enter your answer here*

[ANAM_38] Are you currently in medical treatment because of cancer of the ovaries?
▼ (ANAM_4_2 == “Yes”) AND (ANAM_22_f_8 == “Yes”)
☐ Yes
☐ No

[ANAM_39] When did cancer of the bladder occur for the first time?
Please indicate your age at the time of the first diagnosis or the year of the first diagnosis, whichever you remember better.
▼ (ANAM_4_2 == “Yes”) AND (ANAM_22_f_9 == “Yes” OR ANAM_22_m_9 == “Yes”)
Age: *enter your answer here*
Year: *enter your answer here*

[ANAM_40] Are you currently in medical treatment because of cancer of the bladder?
▼ (ANAM_4_2 == “Yes”) AND (ANAM_22_f_9 == “Yes” OR ANAM_22_m_9 == “Yes”)
☐ Yes
☐ No

[ANAM_41] When did black skin cancer occur for the first time?
Please indicate your age at the time of the first diagnosis or the year of the first diagnosis, whichever you remember better.
▼ (ANAM_4_2 == “Yes”) AND (ANAM_22_f_10 == “Yes” OR ANAM_22_m_10 == “Yes”)
Age: *enter your answer here*
Year: *enter your answer here*

[ANAM_42] Are you currently in medical treatment because of black skin cancer?
▼ (ANAM_4_2 == “Yes”) AND (ANAM_22_f_10 == “Yes” OR ANAM_22_m_10 == “Yes”)
☐ Yes
☐ No

[ANAM_43] When did cancer of the pancreas occur for the first time?
Please indicate your age at the time of the first diagnosis or the year of the first diagnosis, whichever you remember better.
▼ (ANAM_4_2 == “Yes”) AND (ANAM_22_f_11 == “Yes” OR ANAM_22_m_11 == “Yes”)
Age: *enter your answer here*
Year: *enter your answer here*

[ANAM_44] Are you currently in medical treatment because of cancer of the pancreas?
▼ (ANAM_4_2 == “Yes”) AND (ANAM_22_f_11 == “Yes” OR ANAM_22_m_11 == “Yes”)
☐ Yes
☐ No

[ANAM_45] When did cancer of the stomach occur for the first time?
Please indicate your age at the time of the first diagnosis or the year of the first diagnosis, whichever you remember better.
▼ (ANAM_4_2 == “Yes”) AND (ANAM_22_f_12 == “Yes” OR ANAM_22_m_12 == “Yes”)
Age: *enter your answer here*
Year: *enter your answer here*

[ANAM_46] Are you currently in medical treatment because of cancer of the stomach?
▼ (ANAM_4_2 == “Yes”) AND (ANAM_22_f_12 == “Yes” OR ANAM_22_m_12 == “Yes”)
☐ Yes
☐ No

[ANAM_47] When did cancer of the kidney and urinary tract occur for the first time?
Please indicate your age at the time of the first diagnosis or the year of the first diagnosis, whichever you remember better.
▼ (ANAM_4_2 == “Yes”) AND (ANAM_22_f_13 == “Yes” OR ANAM_22_m_13 == “Yes”)
Age: *enter your answer here*
Year: *enter your answer here*

[ANAM_48] Are you currently in medical treatment because of cancer of the kidney and urinary tract?
▼ (ANAM_4_2 == “Yes”) AND (ANAM_22_f_13 == “Yes” OR ANAM_22_m_13 == “Yes”)
☐ Yes
☐ No

[ANAM_49] When did cancer of the mouth and throat occur for the first time?
Please indicate your age at the time of the first diagnosis or the year of the first diagnosis, whichever you remember better.
▼ (ANAM_4_2 == “Yes”) AND (ANAM_22_f_14 == “Yes” OR ANAM_22_m_14 == “Yes”)
Age: *enter your answer here*
Year: *enter your answer here*

[ANAM_50] Are you currently in medical treatment because of cancer of the mouth and throat?
▼ (ANAM_4_2 == “Yes”) AND (ANAM_22_f_14 == “Yes” OR ANAM_22_m_14 == “Yes”)
☐ Yes
☐ No

[ANAM_51] When did non-Hodgkin lymphoma occur for the first time?
Please indicate your age at the time of the first diagnosis or the year of the first diagnosis, whichever you remember better.
▼ (ANAM_4_2 == “Yes”) AND (ANAM_22_f_15 == “Yes” OR ANAM_22_m_15 == “Yes”)
Age: *enter your answer here*
Year: *enter your answer here*

[ANAM_52] Are you currently in medical treatment because of non-Hodgkin lymphoma?
▼ (ANAM_4_2 == “Yes”) AND (ANAM_22_f_15 == “Yes” OR ANAM_22_m_15 == “Yes”)
☐ Yes
☐ No

[ANAM_53] When did Hodgkin's disease occur for the first time?
Please indicate your age at the time of the first diagnosis or the year of the first diagnosis, whichever you remember better.
▼ (ANAM_4_2 == “Yes”) AND (ANAM_22_f_16 == “Yes” OR ANAM_22_m_16 == “Yes”)
Age: *enter your answer here*
Year: *enter your answer here*

[ANAM_54] Are you currently in medical treatment because of Hodgkin's disease?
▼ (ANAM_4_2 == “Yes”) AND (ANAM_22_f_16 == “Yes” OR ANAM_22_m_16 == “Yes”)
☐ Yes
☐ No

[ANAM_55] When did leukemia occur for the first time?
Please indicate your age at the time of the first diagnosis or the year of the first diagnosis, whichever you remember better.
▼ (ANAM_4_2 == “Yes”) AND (ANAM_22_f_17 == “Yes” OR ANAM_22_m_17 == “Yes”)
Age: *enter your answer here*
Year: *enter your answer here*

[ANAM_56] Are you currently in medical treatment because of leukemia?
▼ (ANAM_4_2 == “Yes”) AND (ANAM_22_f_17 == “Yes” OR ANAM_22_m_17 == “Yes”)
☐ Yes
☐ No

[ANAM_57] When did cancer of the liver occur for the first time?
Please indicate your age at the time of the first diagnosis or the year of the first diagnosis, whichever you remember better.
▼ (ANAM_4_2 == “Yes”) AND (ANAM_22_f_18 == “Yes” OR ANAM_22_m_18 == “Yes”)
Age: *enter your answer here*
Year: *enter your answer here*

[ANAM_58] Are you currently in medical treatment because of cancer of the liver?
▼ (ANAM_4_2 == “Yes”) AND (ANAM_22_f_18 == “Yes” OR ANAM_22_m_18 == “Yes”)
☐ Yes
☐ No

[ANAM_59] When did cancer of the esophagus occur for the first time?
Please indicate your age at the time of the first diagnosis or the year of the first diagnosis, whichever you remember better.
▼ (ANAM_4_2 == “Yes”) AND (ANAM_22_f_19 == “Yes” OR ANAM_22_m_19 == “Yes”)
Age: *enter your answer here*
Year: *enter your answer here*

[ANAM_60] Are you currently in medical treatment because of cancer of the esophagus?
▼ (ANAM_4_2 == “Yes”) AND (ANAM_22_f_19 == “Yes” OR ANAM_22_m_19 == “Yes”)
☐ Yes
☐ No

[ANAM_61] When did cancer of the brain occur for the first time?
Please indicate your age at the time of the first diagnosis or the year of the first diagnosis, whichever you remember better.
▼ (ANAM_4_2 == “Yes”) AND (ANAM_22_f_20 == “Yes” OR ANAM_22_m_20 == “Yes”)
Age: *enter your answer here*
Year: *enter your answer here*

[ANAM_62] Are you currently in medical treatment because of cancer of the brain?
▼ (ANAM_4_2 == “Yes”) AND (ANAM_22_f_20 == “Yes” OR ANAM_22_m_20 == “Yes”)
☐ Yes
☐ No

[ANAM_63] When did cancer of the thyroid occur for the first time?
Please indicate your age at the time of the first diagnosis or the year of the first diagnosis, whichever you remember better.
▼ (ANAM_4_2 == “Yes”) AND (ANAM_22_f_21 == “Yes” OR ANAM_22_m_21 == “Yes”)
Age: *enter your answer here*
Year: *enter your answer here*

[ANAM_64] Are you currently in medical treatment because of cancer of the thyroid?
▼ (ANAM_4_2 == “Yes”) AND (ANAM_22_f_21 == “Yes” OR ANAM_22_m_21 == “Yes”)
☐ Yes
☐ No

[ANAM_65] When did cancer of the bones occur for the first time?
Please indicate your age at the time of the first diagnosis or the year of the first diagnosis, whichever you remember better.
▼ (ANAM_4_2 == “Yes”) AND (ANAM_22_f_22 == “Yes” OR ANAM_22_m_22 == “Yes”)
Age: *enter your answer here*
Year: *enter your answer here*

[ANAM_66] Are you currently in medical treatment because of cancer of the bones?
▼ (ANAM_4_2 == “Yes”) AND (ANAM_22_f_22 == “Yes” OR ANAM_22_m_22 == “Yes”)
☐ Yes
☐ No

[ANAM_67] When did cancer of the larynx occur for the first time?
Please indicate your age at the time of the first diagnosis or the year of the first diagnosis, whichever you remember better.
▼ (ANAM_4_2 == “Yes”) AND (ANAM_22_f_23 == “Yes” OR ANAM_22_m_23 == “Yes”)
Age: *enter your answer here*
Year: *enter your answer here*

[ANAM_68] Are you currently in medical treatment because of cancer of the larynx?
▼ (ANAM_4_2 == “Yes”) AND (ANAM_22_f_23 == “Yes” OR ANAM_22_m_23 == “Yes”)
☐ Yes
☐ No

[ANAM_69] When did cancer of the gallbladder occur for the first time?
Please indicate your age at the time of the first diagnosis or the year of the first diagnosis, whichever you remember better.
▼ (ANAM_4_2 == “Yes”) AND (ANAM_22_f_24 == “Yes” OR ANAM_22_m_24 == “Yes”)
Age: *enter your answer here*
Year: *enter your answer here*

[ANAM_70] Are you currently in medical treatment because of cancer of the gallbladder?
▼ (ANAM_4_2 == “Yes”) AND (ANAM_22_f_24 == “Yes” OR ANAM_22_m_24 == “Yes”)
☐ Yes
☐ No

[ANAM_71] When did {ANAM_22_f_other}{ANAM_22_m_other} occur for the first time?
Please indicate your age at the time of the first diagnosis or the year of the first diagnosis, whichever you remember better.
▼ (ANAM_4_2 == “Yes”) AND (! (is_empty(ANAM_22_f_other)) OR (! (is_empty(ANAM_22_m_other))
Age: *enter your answer here*
Year: *enter your answer here*

[ANAM_72] Are you currently in medical treatment because of {ANAM_22_f_other}{ANAM_22_m_other}?
▼ (ANAM_4_2 == “Yes”) AND (! (is_empty(ANAM_22_f_other)) OR (! (is_empty(ANAM_22_m_other))
☐ Yes
☐ No

5: Metabolic diseases

[ANAM_73] Which metabolic disease is / was it?
(multiple choice possible)
▼ ANAM_4_3 == “Yes”
☐ Sugar disease or diabetes mellitus
☐ Increased blood lipids or cholesterol or triglycerides
☐ Gout or a uric acid disease
☐ Hyperfunction of the thyroid gland (hyperthyroidism)
☐ Underactive thyroid (hypothyroidism)
☐ Others, namely:

[ANAM_74] When did diabetes occur for the first time?
Please indicate your age at the time of the first diagnosis or the year of the first diagnosis, whichever you remember better.
▼ (ANAM_4_3 == “Yes”) AND (ANAM_73_1 == “Yes”)
Age: *enter your answer here*
Year: *enter your answer here*

[ANAM_75] Have you been diagnosed with diabetes for the first time during pregnancy?
▼ (ANAM_4_3 == “Yes”) AND (ANAM_73_1 == “Yes”) AND (sex_ON11 == “2”)
☐ Yes
☐ No
☐ Don’t know

[ANAM_76] How are you currently being treated? Please also think of injected insulin and insulin pumps.
▼ (ANAM_4_3 == “Yes”) AND (ANAM_73_1 == “Yes”)
☐ Only with insulin
☐ Only with tablets
☐ With insulin and tablets
☐ Only dietary
☐ No treatment
☐ Don‘t know
☐ Others, namely:

[ANAM_77] How long have you been treated?
Please indicate either your age at the beginning of the treatment or the year of commencement of treatment, whichever you remember better.
▼ (ANAM_4_3 == “Yes”) AND (ANAM_73_1 == “Yes”) AND (ANAM_76 != “No treatment”)
Age: *enter your answer here*
Year: *enter your answer here*

[ANAM_78] When did increased blood lipids occur for the first time?
Please indicate your age at the time of the first diagnosis or the year of the first diagnosis, whichever you remember better.
▼ (ANAM_4_3 == “Yes”) AND (ANAM_73_2 == “Yes”)
Age: *enter your answer here*
Year: *enter your answer here*

[ANAM_79] Are you currently in medical treatment because of increased blood lipids?
▼ (ANAM_4_3 == “Yes”) AND (ANAM_73_2 == “Yes”)
☐ Yes
☐ No

[ANAM_80] When did gout occur for the first time?
Please indicate your age at the time of the first diagnosis or the year of the first diagnosis, whichever you remember better.
▼ (ANAM_4_3 == “Yes”) AND (ANAM_73_3 == “Yes”)
Age: *enter your answer here*
Year: *enter your answer here*

[ANAM_81] Are you currently in medical treatment because of gout?
▼ (ANAM_4_3 == “Yes”) AND (ANAM_73_3 == “Yes”)
☐ Yes
☐ No

[ANAM_82] When did {if(ANAM_73_4 == “Yes”, “hyperthyroidism”, “hypothyroidism”)} occur for the first time?
Please indicate your age at the time of the first diagnosis or the year of the first diagnosis, whichever you remember better.
▼ (ANAM_4_3 == “Yes”) AND ((ANAM_73_4 == “Yes”) OR (ANAM_73_5 == “Yes”))
Age: *enter your answer here*
Year: *enter your answer here*

[ANAM_83] How are you currently being treated?
▼ (ANAM_4_3 == “Yes”) AND ((ANAM_73_4 == “Yes”) OR (ANAM_73_5 == “Yes”))
☐ Thyroid hormones
☐ Radioiodine therapy
☐ Irradiation in the neck area
☐ Thyroid surgery
☐ Iodine tablets
☐ No treatment
☐ Don‘t know
☐ Others, namely:

[ANAM_84] How long have you been treated?
Please indicate either your age at the beginning of the treatment or the year of commencement of treatment, whichever you remember better.
▼ (ANAM_4_3 == “Yes”) AND ((ANAM_73_4 == “Yes”) OR (ANAM_73_5 == “Yes”)) AND (ANAM_83 != “No treatment”)
Age: *enter your answer here*
Year: *enter your answer here*

[ANAM_85] When did {ANAM_73_other} occur for the first time?
Please indicate your age at the time of the first diagnosis or the year of the first diagnosis, whichever you remember better.
▼ (ANAM_4_3 == “Yes”) AND (! (is_empty(ANAM_73_other))
Age: *enter your answer here*
Year: *enter your answer here*

[ANAM_86] Are you currently in medical treatment because of {ANAM_73_other}?
▼ (ANAM_4_3 == “Yes”) AND (! (is_empty(ANAM_73_other))
☐ Yes
☐ No

6: Musculoskeletal diseases

[ANAM_87] When did a musculoskeletal disease occur for the first time?
Please indicate your age at the time of the first diagnosis or the year of the first diagnosis, whichever you remember better.
▼ ANAM_4_4 == “Yes”
Age: *enter your answer here*
Year: *enter your answer here*

[ANAM_88] Which musculoskeletal disease is / was it?
enter your answer here

[ANAM_89] Are you currently in medical treatment because of {ANAM_88}?
☐ Yes
☐ No

7: Lung diseases

[ANAM_90] When did a lung disease occur for the first time?
Please indicate your age at the time of the first diagnosis or the year of the first diagnosis, whichever you remember better.
▼ ANAM_4_5 == “Yes”
Age: *enter your answer here*
Year: *enter your answer here*

[ANAM_91] Which lung disease is / was it?
enter your answer here

[ANAM_92] Are you currently in medical treatment because of {ANAM_91}?
☐ Yes
☐ No

8: Allergies

[ANAM_93] Which allergy is / was it?
(multiple choice possible)
▼ ANAM_4_6 == “Yes”
☐ Hay fever (pollen allergy)
☐ Insect venom allergy
☐ Food allergy
☐ House dust allergy
☐ Animal hair allergy
☐ Contact allergy
☐ Drug allergy
☐ Others, namely:

[ANAM_94] When did hay fever occur for the first time?
Please indicate your age at the time of the first diagnosis or the year of the first diagnosis, whichever you remember better.
▼ (ANAM_4_6 == “Yes”) AND (ANAM_93_1 == “Yes”)
Age: *enter your answer here*
Year: *enter your answer here*

[ANAM_95] Are you currently in medical treatment because of hay fever?
▼ (ANAM_4_6 == “Yes”) AND (ANAM_93_1 == “Yes”)
☐ Yes
☐ No

[ANAM_96] When did insect venom allergy occur for the first time?
Please indicate your age at the time of the first diagnosis or the year of the first diagnosis, whichever you remember better.
▼ (ANAM_4_6 == “Yes”) AND (ANAM_93_2 == “Yes”)
Age: *enter your answer here*
Year: *enter your answer here*

[ANAM_97] Are you currently in medical treatment because of insect venom allergy?
▼ (ANAM_4_6 == “Yes”) AND (ANAM_93_2 == “Yes”)
☐ Yes
☐ No

[ANAM_98] When did food allergy occur for the first time?
Please indicate your age at the time of the first diagnosis or the year of the first diagnosis, whichever you remember better.
▼ (ANAM_4_6 == “Yes”) AND (ANAM_93_3 == “Yes”)
Age: *enter your answer here*
Year: *enter your answer here*

[ANAM_99] Are you currently in medical treatment because of food allergy?
▼ (ANAM_4_6 == “Yes”) AND (ANAM_93_3 == “Yes”)
☐ Yes
☐ No

[ANAM_100] When did house dust allergy occur for the first time?
Please indicate your age at the time of the first diagnosis or the year of the first diagnosis, whichever you remember better.
▼ (ANAM_4_6 == “Yes”) AND (ANAM_93_4 == “Yes”)
Age: *enter your answer here*
Year: *enter your answer here*

[ANAM_101] Are you currently in medical treatment because of house dust allergy?
▼ (ANAM_4_6 == “Yes”) AND (ANAM_93_4 == “Yes”)
☐ Yes
☐ No

[ANAM_102] When did animal hair allergy occur for the first time?
Please indicate your age at the time of the first diagnosis or the year of the first diagnosis, whichever you remember better.
▼ (ANAM_4_6 == “Yes”) AND (ANAM_93_5 == “Yes”)
Age: *enter your answer here*
Year: *enter your answer here*

[ANAM_103] Are you currently in medical treatment because of animal hair allergy?
▼ (ANAM_4_6 == “Yes”) AND (ANAM_93_5 == “Yes”)
☐ Yes
☐ No

[ANAM_104] When did contact allergy occur for the first time?
Please indicate your age at the time of the first diagnosis or the year of the first diagnosis, whichever you remember better.
▼ (ANAM_4_6 == “Yes”) AND (ANAM_93_6 == “Yes”)
Age: *enter your answer here*
Year: *enter your answer here*

[ANAM_105] Are you currently in medical treatment because of contact allergy?
▼ (ANAM_4_6 == “Yes”) AND (ANAM_93_6 == “Yes”)
☐ Yes
☐ No

[ANAM_106] When did drug allergy occur for the first time?
Please indicate your age at the time of the first diagnosis or the year of the first diagnosis, whichever you remember better.
▼ (ANAM_4_6 == “Yes”) AND (ANAM_93_7 == “Yes”)
Age: *enter your answer here*
Year: *enter your answer here*

[ANAM_107] Are you currently in medical treatment because of drug allergy?
▼ (ANAM_4_6 == “Yes”) AND (ANAM_93_7 == “Yes”)
☐ Yes
☐ No

[ANAM_108] When did {ANAM_93_other} occur for the first time?
Please indicate your age at the time of the first diagnosis or the year of the first diagnosis, whichever you remember better.
▼ (ANAM_4_6 == “Yes”) AND (! (is_empty(ANAM_93_other))
Age: *enter your answer here*
Year: *enter your answer here*

[ANAM_109] Are you currently in medical treatment because of {ANAM_93_other}?
▼ (ANAM_4_6 == “Yes”) AND (! (is_empty(ANAM_93_other))
☐ Yes
☐ No

9: Gastrointestinal or liver diseases

[ANAM_110] When did a gastrointestinal or liver disease occur for the first time?
Please indicate your age at the time of the first diagnosis or the year of the first diagnosis, whichever you remember better.
▼ ANAM_4_7 == “Yes”
Age: *enter your answer here*
Year: *enter your answer here*

[ANAM_111] Which gastrointestinal or liver disease is / was it?
enter your answer here

[ANAM_112] Are you currently in medical treatment because of {ANAM_111}?
☐ Yes
☐ No

10: Skin diseases

[ANAM_113] When did a skin disease occur for the first time?
Please indicate your age at the time of the first diagnosis or the year of the first diagnosis, whichever you remember better.
▼ ANAM_4_8 == “Yes”
Age: *enter your answer here*
Year: *enter your answer here*

[ANAM_114] Which skin disease is / was it?
enter your answer here

[ANAM_115] Are you currently in medical treatment because of {ANAM_114}?
- ☐ Yes
- ☐ No

11: Kidney diseases

[ANAM_116] When did a kidney disease occur for the first time?
Please indicate your age at the time of the first diagnosis or the year of the first diagnosis, whichever you remember better.
▼ ANAM_4_9 == “Yes”
Age: *enter your answer here*
Year: *enter your answer here*

[ANAM_117] Which kidney disease is / was it?
enter your answer here

[ANAM_118] Are you currently in medical treatment because of {ANAM_117}?
☐ Yes
☐ No

[ANAM_119] Have you ever been treated with a dialysis (blood wash)?
☐ Yes
☐ No

12: Neurological diseases

[ANAM_120] When did a neurological disease occur for the first time?
Please indicate your age at the time of the first diagnosis or the year of the first diagnosis, whichever you remember better.
▼ ANAM_4_10 == “Yes”
Age: *enter your answer here*
Year: *enter your answer here*

[ANAM_121] Which neurological disease is / was it?
enter your answer here

[ANAM_122] Are you currently in medical treatment because of {ANAM_121}?
☐ Yes
☐ No

13: Psychiatric diseases

[ANAM_123] When did a psychiatric disease occur for the first time?
Please indicate your age at the time of the first diagnosis or the year of the first diagnosis, whichever you remember better.
▼ ANAM_4_11 == “Yes”
Age: *enter your answer here*
Year: *enter your answer here*

[ANAM_124] Which psychiatric disease is / was it?
enter your answer here

[ANAM_125] Are you currently in medical treatment because of {ANAM_124}?
☐ Yes
☐ No

14: Other diseases

[ANAM_126] When did {ANAM_4_12_text} occur for the first time?
Please indicate your age at the time of the first diagnosis or the year of the first diagnosis, whichever you remember better.
▼ ANAM_4_12 == “Yes”
Age: *enter your answer here*
Year: *enter your answer here*

[ANAM_127] Are you currently in medical treatment because of {ANAM_4_12_text}?
☐ Yes
☐ No

15: Seeking medical advice

[Arzt_1] How often have you visited a doctor (except for dentists) in the last 12 months?
In answering this question, please refer to all doctors (except for dentists) who you have visited in the past 12 months, e.g. general practitioner, ophthalmologist, dermatologist or other specialists.

☐ In the last 12 months I have not visited a doctor.
☐ Number of visits:

[Arzt_2] For what reason(s) did you visit a doctor in the last 12 months?
(multiple choice possible)
▼ Arzt_1 == Yes"

☐ Acute disease, e.g. flu, diarrhea, accident
☐ Disease which is not an infection, e.g. sugar disease, hypertension, allergies
☐ Feeling ill, e.g. general malaise, sleep diseases
☐ Consultation
☐ Visit without medical consultation, e.g. prescription, irradiation
☐ Preventive examination, vaccination

[Arzt_3] Do you have a general practioner?

☐ Yes
☐ No

16: Smoking

[smoke_1] Have you ever smoked regularly in your life over a period of more than 6 months?
Under “regularly” we understand: 1 cigarette per day or at least 5 cigarettes per week or at least 1 pack of cigarettes per month or 1 cigarillo per day or at least 5 per week or 2 cigars per week or 2 pipes per week.

☐ Yes
☐ No

[smoke_2] When did you start regular smoking?
Please specify either your age or the year in which you started smoking, depending on what you remember better.
▼ smoke_1 == “Yes”
Age: *enter your answer here*
Year: *enter your answer here*

[smoke_3] Do you smoke at the moment, though occasionally?
▼ (smoke_1 == “Yes”

☐ Yes, every day
☐ Yes, occasionally
☐ No

How much do you currently smoke?
Please specify the number per day / per week or grams per week depending on what you can estimate better.

[smoke_4_1] Filter cigarettes (ready to use)
▼ (smoke_1 == “Yes”) AND (smoke_3 == “Yes”)
Number per day: *enter your answer here*
Number per week: *enter your answer here*

[smoke_4_2] Filterless cigarettes (ready to use)
▼ (smoke_1 == “Yes”) AND (smoke_3 == “Yes”)
Number per day: *enter your answer here*
Number per week: *enter your answer here*

[smoke_4_3] Cigars, cigarillos, stumps
▼ (smoke_1 == “Yes”) AND (smoke_3 == “Yes”)
Number per day: *enter your answer here*
Number per week: *enter your answer here*

[smoke_4_4] Self-turned cigarettes
▼ (smoke_1 == “Yes”) AND (smoke_3 == “Yes”)
Grams per week: *enter your answer here*
Number per day: *enter your answer here*

[smoke_4_5] Pipes
▼ (smoke_1 == “Yes”) AND (smoke_3 == “Yes”)
Grams per week: *enter your answer here*
Number per day: *enter your answer here*

[smoke_5] When did you stop smoking?
Please specify either your age or the year in which you stopped smoking, depending on what you remember better.
▼ (smoke_1 == “Yes”) AND (smoke_3 == “No”)
Age: *enter your answer here*
Year: *enter your answer here*

How much have you usually smoked in the past?
Please enter the number per day / per week or per week depending on what you can estimate.

[smoke_6_1] Filter cigarettes (ready to use)
▼ (smoke_1 == “Yes”) AND (smoke_3 == “No”)
Number per day: *enter your answer here*
Number per week: *enter your answer here*

[smoke_6_2] Filterless cigarettes (ready to use)
▼ (smoke_1 == “Yes”) AND (smoke_3 == “No”)
Number per day: *enter your answer here*
Number per week: *enter your answer here*

[smoke_6_3] Cigars, cigarillos, stumps
▼ (smoke_1 == “Yes”) AND (smoke_3 == “No”)
Number per day: *enter your answer here*
Number per week: *enter your answer here*

[smoke_6_4] Self-turned cigarettes
▼ (smoke_1 == “Yes”) AND (smoke_3 == “No”)
Grams per week: *enter your answer here*
Number per day: *enter your answer here*

[smoke_6_5] Pipes
▼ (smoke_1 == “Yes”) AND (smoke_3 == “No”)
Grams per week: *enter your answer here*
Number per day: *enter your answer here*

17: Alcohol consumption

[AUDIT_1] How often do you drink alcohol?
- ☐ Never
- ☐ About once a month
- ☐ Two to four times a month
- ☐ Two to three times a week
- ☐ Four times or more per week

[AUDIT_2] If you drink alcohol in one day, how many alcoholic drinks do you typically drink?
- ☐ 1 or 2 glasses
- ☐ 3 or 4 glasses
- ☐ 5 or 6 glasses
- ☐ 7 or 8 glasses
- ☐ 10 glasses or more

[AUDIT_3] How often have you drunk more than 6 alcoholic beverages in the last 12 months?
- ☐ Never
- ☐ Less than 1 time per month
- ☐ Once a month
- ☐ Once a week
- ☐ Daily or almost daily

18: Sexual contacts

In the following, we are now asking for sexual contacts, that is, those with whom sexual intercourse has taken place. There are diseases that can be transmitted via sexual intercourse. The transmission takes place only sometimes and not everyone is affected. We ask these questions to understand the possible spread of these pathogens. This is not about whether someone was or is ill. It is also very important to get answers from people who don’t have sexually transmitted diseases. Again, we would like to stress at this point that your answers will remain anonymous and no individual responses will be visible. The answer to the questions is, of course, voluntary.

[keine_Antwort] If you don’t want to complete this section of the survey, please let us know.
In the course of the survey you have also the option "I don’t want to answer".
☐ I don’t want to complete this section of the survey.

[sex1] How old were you when you first had sexual intercourse?
If you are not sure how old you were, please give an estimate.
▼ keine_Antwort_1 != “Yes”
☐ I don’t want to answer.
☐ I have not had any sexual contact yet.
☐ My age in years was then:

[sex2] Do you currently have a sexual partnership?
Sexual partnership means: a partnership in which sexual intercourse takes place.
▼ (keine_Antwort_1 != “Yes”) & (sex1 != “I have not had any sexual contact yet.”)
☐ Yes
☐ No
☐ I don’t want to answer.

[sex2_dauer] Do you think that this sexual partnership will continue in 3 months?
▼ sex2 == “Yes”
☐ Yes
☐ No
☐ Don‘t know
☐ I don’t want to answer.

19: Contacts with persons of the opposite sex

[sex3] If you think about your whole life, with how many people of the opposite sex have you had sexual intercourse so far?

enter your answer here

☐ I don’t want to answer.

[sex4] If you think about the last 12 months (since June 1, 2014), with how many people of the opposite sex did you have sexual intercourse?

▼ sex3_1_anz >= “1”

enter your answer here

☐ I don’t want to answer.

[sex4neu] How many of these {sex4_1_anz} people you have had sexual intercourse with over the past 12 months have been new partners with whom you have had sexual intercourse for the first time?

▼ sex4_1_anz >= “1”

enter your answer here

☐ I don’t want to answer.

20: Contacts with persons of the same sex

[sex5] If you think about your entire life, with how many people of the same sex have you had sexual intercourse so far?

enter your answer here

☐ I don’t want to answer.

[sex6] If you think about the last 12 months (since June 1, 2014), with how many people of the same sex did you have sexual intercourse?

▼ sex5_1_anz >= “1”

enter your answer here

☐ I don’t want to answer.

[sex6neu] How many of these {sex6_1_anz} people you have had sexual intercourse with over the last 12 months have been new partners with whom you had sexual intercourse for the first time?

▼ sex6_1_anz >= “1”

enter your answer here

☐ I don’t want to answer.

21: Age of contact persons

[sex7_99] How old were the last partners with whom you had sexual intercourse?
Please refer only to your sexual partners during the last 12 months (since June 1, 2014).
Please also provide details of how often you / your partner used condoms when having sexual intercourse.
☐ I don’t want to answer.

If you don’t know the exact age, please give your best estimate of age.

		Gender		Frequency of condom use		
	Age	Male	Female	Always (for every sexual intercourse)	Sometimes yes, sometimes no	Never
[1]		☐	☐	☐	☐	☐
[2]		☐	☐	☐	☐	☐
[3]		☐	☐	☐	☐	☐

22: Contacts of the partner

{if (sex2 ==“Yes”, “The following questions pertain to your current partner(s). "," The following questions refer to your last sexual partnership. ")}
[sex9] Did your partner have sexual intercourse with other people in the last 12 months (since June 1, 2014)?
☐ Yes (I know exactly)
☐ Yes (I suppose, but I don’t know)
☐ No (I know exactly)
☐ I don’t know it
☐ I don’t want to answer.

[sex9_anz] Do you know how many sexual partners your partner has had in the last 12 months (excluding yourself)?
▼ sex9 == “Yes
☐ No, I don’t know that.
☐ I don’t want to answer.
☐ Yes, number of partners:

[sex10] Do you know how many sexual partners your partner has had in your life (excluding yourself)?
☐ No, I don’t know that.
☐ I don’t want to answer.
☐ Yes, number of partners:

[sex11] How often did your partner use a condom in the past partnerships in his / her life?
☐ Always (for every sexual intercourse)
☐ Sometimes yes, sometimes no
☐ Never
☐ Do not know
☐ I do not want to answer.

23: Submit questionnaire

[ON11_remark] Thank you for completing the survey!
If you would like to tell us something about this survey, you can do it here:
enter your answer here

L: Ebola risk perception (follow-up)

In November 2014, we asked you what you think about the Ebola outbreak in West Africa and whether / how Ebola affects your everyday life. We would now like to examine whether these aspects have changed over time. That is why we ask you a few questions from November, some of them in changed form.

1: Ebola virus disease

[angst1_2015] Are you currently worried about Ebola?
☐ Yes
☐ No

[angst_reason_2015] Why are you worried?
(multiple choice possible)
▼ angst1_2015 == "Yes"
☐ Worried to get infected with Ebola yourself
☐ Worried that a family members gets infected with Ebola
☐ Worried that the outbreak could turn into a pandemic
☐ Worried that many people die in the world
☐ Other reason, namely:

[angst2_2015] How much are you worried about Ebola?
Please mark a number from 1="not much" to 5="very much"
▼ angst1_2015 == "Yes"

Not much			Very much	
1	2	3	4	5
☐	☐	☐	☐	☐

[angst1_2014] At the time of the last survey (November 2014): Were you worried about Ebola?
☐ Yes
☐ No

[angst_reason_2014] Why were you worried at that time?
(multiple choice possible)
▼ angst1_2014 == "Yes"
☐ Worried to get infected with Ebola yourself
☐ Worried that a family members gets infected with Ebola
☐ Worried that the outbreak could turn into a pandemic
☐ Worried that many people die in the world
☐ Other reason, namely:

2: Ebola virus disease (continued)

[wissen1_2015] How do you rate your current personal knowledge about Ebola virus disease?
- ☐ Very poor
- ☐ Poor
- ☐ Moderate
- ☐ Good
- ☐ Very good

[wissen2_2015] How do you rate the current information that you get from the media about the situation in African countries affected by Ebola?
- ☐ Very poor
- ☐ Poor
- ☐ Moderate
- ☐ Good
- ☐ Very good

3: Probability of acquiring Ebola

[knowledge_2015] How can Ebola be transmitted?

	Yes	No	Don’t know
By direct contact with bodily fluids of infected persons, either dead or living	☐	☐	☐
By direct contact with infected, but asymptomatic persons	☐	☐	☐
Through air, if infected people cough or sneeze	☐	☐	☐
Through material which has been heavily contaminated with bodily fluids of dead or living infected persons	☐	☐	☐
Through drinking water	☐	☐	☐
Through food produced in Germany	☐	☐	☐
By casual contact with someone already sick, such as sitting next to the person (without any direct contact of bodily fluids)	☐	☐	☐
By wild animals in Africa (monkeys, bats)	☐	☐	☐
By wild animals in Germany (rats, foxes)	☐	☐	☐
By insects in Africa (mosquitoes, tsetse flies)	☐	☐	☐
By insects in Germany (midges)	☐	☐	☐

4: Probability of acquiring Ebola (continued)

[risk_2015] If you think of the recent worldwide situation about Ebola: Do you think that you have a personal risk of acquiring Ebola…

	Highly likely	Quite likely	Quite unlikely	Highly unlikely	Does not apply
… at work?	☐	☐	☐	☐	☐
... in public transport?	☐	☐	☐	☐	☐
... in public places (school, childcare …) or public events?	☐	☐	☐	☐	☐
… at an airport in Germany?	☐	☐	☐	☐	☐
… as a patient in a German hospital?	☐	☐	☐	☐	☐
… at a doctor’s office in Germany?	☐	☐	☐	☐	☐
… during a travel to affected countries?	☐	☐	☐	☐	☐
… by food imported from Western African countries?	☐	☐	☐	☐	☐
... by other products originating in West Africa?	☐	☐	☐	☐	☐

5: Probability of acquiring Ebola (continued)

[pandemie_2015] Are you worried that…

	Highly likely	Quite likely	Quite unlikely	Highly unlikely
… in the next three months people might arrive in Germany who are identified as infected persons after their entry?	☐	☐	☐	☐
… individual persons might be infected with the Ebola virus in Germany during the next six months?	☐	☐	☐	☐
… in the next six months Ebola could spread in the general population of Germany similar to how it is spreading currently in West Africa?	☐	☐	☐	☐

6: Personal behavior and prevention measures

[travel1_2015] Consider the following scenario: You have won a trip to Africa. You like Africa as a destination and you are happy about winning.
If you are thinking of the current global situation regarding Ebola: Would you take this trip if the trip would go to one of the affected areas in West Africa?

☐ Yes
☐ No
☐ Don't know

[travel2_2015] Consider the following scenario: You have won a trip to Africa. You like Africa as a destination and you are happy about winning.
If you are thinking of the current global situation regarding Ebola: Would you take this trip if the trip would go to non-affected parts of Africa?

☐ Yes
☐ No
☐ Don't know

[travel_result] Between March 2014 and today: Did you actually cancel a journey to Africa because of Ebola?

☐ Yes, I cancelled a journey to Africa due to the Ebola outbreak.
☐ In the period from March 2014 until today, I did not plan / undertake any trip to Africa at all.
☐ I have made the trip to Africa as planned.

[travel_destination] In which African country {if(travel_result == “made the trip”, “did you travel”, “did you plan to travel”)}?
▼ travel_result == “cancelled” | travel_result == “made the trip”
enter your answer here

7: Personal behavior and prevention measures (continued)

[action_hospital_2015] Would you change your behavior if an Ebola patient was evacuated from Africa and brought to Germany for treatment in a near-by hospital?

	Yes	Rather yes	Rather no	No
I would avoid public events and crowded places.	☐	☐	☐	☐
I would avoid using public transport.	☐	☐	☐	☐
I would avoid physical contact with other people.	☐	☐	☐	☐
I would increase my hygiene behavior (e.g. wash my hands more often)	☐	☐	☐	☐
I would wear a face mask outside of my home.	☐	☐	☐	☐
I would not want to be admitted to the same hospital.	☐	☐	☐	☐
I would not visit friends admitted to the same hospital.	☐	☐	☐	☐

8: Personal behavior and prevention measures (continued)

[measure_2015] Should the following measures be introduced to prevent the spread of Ebola to Europe?

	Not on any account	Not encouraged	Encouraged	Yes, absolutely
Provide information on Ebola to all travelers coming from affected areas and provide advice in case one develops signs and symptoms	☐	☐	☐	☐
Get personal information of all travelers coming from affected areas and control their health for three weeks long upon arrival	☐	☐	☐	☐
Forbid return transport for Germans who get infected during aid missions in West Africa	☐	☐	☐	☐
Forbid bringing Ebola patients for treatment to Germany	☐	☐	☐	☐
Measure temperature for all travelers coming from affected countries upon arrival at Europe with subsequent quarantine for those with high temperature	☐	☐	☐	☐
Measure temperature for all travelers coming from affected countries when they are about to leave Africa with subsequent quarantine for those with high temperature	☐	☐	☐	☐
Three weeks of mandatory quarantine for all volunteers returning from aid missions in West Africa	☐	☐	☐	☐
Entry restrictions for people from affected countries	☐	☐	☐	☐
Forbid travelling from Germany to affected countries in Africa	☐	☐	☐	☐
Compulsory vaccination against Ebola for all inhabitants of affected countries as soon as a vaccine is available	☐	☐	☐	☐

9: Personal commitment

[help1_2015] Suppose there was another major outbreak of an infectious disease in Africa: Would you go there to help?

- ☐ Yes
- ☐ Unlikely
- ☐ Likely
- ☐ No
- ☐ Don’t know

[help1_1_2015] Do you think that your experience/knowledge would be helpful to do that?

- ☐ Yes
- ☐ Unlikely
- ☐ Likely
- ☐ No
- ☐ Don’t know

[help1_2_2015] Would your personal situation allow you to go and help in Africa?
(multiple choice possible)

- ☐ Yes
- ☐ No, I cannot go because of my family
- ☐ No, I cannot go because of my job
- ☐ Don't know
- ☐ Other (please specify):

[help2_2015] What would be the main reason not to volunteer?

- ☐ I would be worried about getting infected.
- ☐ I think that the help would not be useful.
- ☐ I would be afraid to be overwhelmed by the situation on site.
- ☐ I would be worried that I might not return to Germany if I get infected.
- ☐ I would be worried about not being able to get back to Germany because of an entry restriction.
- ☐ I think every country should solve its problems by itself without depending on help from other countries.
- ☐ Other reason, namely:

[help3_2015] Do you know someone who has helped in Africa during the Ebola outbreak?

- ☐ Yes
- ☐ No

10: Personal commitment (continued)

[money1_2015] Would you be willing to donate for the fight against Ebola in Africa?

☐ Yes ☐ Unlikely
☐ Likely ☐ No
☐ Don't know

[money2_2015] Which sum would you donate?
If you have already donated: How much have you donated?
▼ money1_2015 != "No"

☐ Up to 10 Euros
☐ 11 to 20 Euros
☐ 21 to 50 Euros
☐ 51 to 100 Euros
☐ 101 to 200 Euros
☐ More than 200 Euros

[money3_2015] Would you support a nonrecurring, compulsory, and income-related payment for the fight against Ebola in Africa?

☐ Yes ☐ Unlikely
☐ Likely ☐ No
☐ Don't know

[money4_2015] Which sum would you pay for the cause?
Please enter as a percentage of your monthly net income.
▼ money3_2015 != "No"
enter your answer here

11: Vaccination

[vaccination1_2015] If a vaccine against Ebola existed, would you opt for the vaccination even if you do not plan to visit affected countries in West Africa and do not have contact with Ebola patients ever?

☐ Yes
☐ No
☐ Don't know

[vaccination2_2015] Would you still do so if the vaccine was associated with occasional mild side effects?
▼ vaccination1_2015 != "No"

☐ Yes
☐ No
☐ Don't know

[vaccination3_2015] Would you still do so if the vaccine was associated with rare and severe side effects?
▼ vaccination1_2015 != "No"

☐ Yes
☐ No
☐ Don't know

[vaccination4_2015] Should there be a compulsory vaccination against Ebola for the medical staff in Germany?

☐ Yes
☐ No
☐ Don't know

[vaccination5_2015] Should there be a compulsory vaccination against Ebola for the general population in Germany if the number of Ebola cases in Germany increased?

☐ Yes
☐ No
☐ Don't know

12: Activities about Ebola

[activity_2015] Did you do something among the following list of actions in connection to Ebola?
(multiple choice possible)

- ☐ Write a letter to the editor or a blog entry about Ebola in a paper or on the Internet
- ☐ Write a comment to an article in the Internet (e.g. Tagesschau.de, Spiegel online)
- ☐ Discussion in the circle of acquaintances
- ☐ Adhered to preventive measures at work
- ☐ Donations
- ☐ Participation in information events
- ☐ Organizing an information event
- ☐ Offer to participate in aid missions in Africa
- ☐ Offer to help in Germany

M: Tick-borne infections (follow-up)

In July 2014 we interviewed you about ticks and tick infections. Here we could get interesting results. We would like to ask you more about this topic in order to be able to interpret them more precisely. You already know some of the questions from the first questionnaire on this topic. Nevertheless, please answer all questions in this questionnaire.

1: Fear

[ZE_2_1] Are you afraid of ticks?
☐ Yes
☐ No

[ZE_2_2] I am afraid…
▼ ZE_2_1 == “Yes”

	Applies	Rather applies	Does rather not apply	Does not apply
[1] … to get infected with borreliosis.	☐	☐	☐	☐
[2] … to get infected with early summer meningoencephalitis (TBE).	☐	☐	☐	☐
[3] … of ticks (spiders) in general.	☐	☐	☐	☐
[4] …to have to remove the tick.	☐	☐	☐	☐

2: Prevention – tick vaccination

[ZE_2_3] Have you ever received a tick vaccination?
☐ Yes
☐ No
☐ Don’t know

[ZE_2_4] How many times have you been vaccinated against ticks in your life (1 injection corresponds to a vaccination)?
If you do not know the exact number, please provide an estimate.
▼ ZE_2_3 == “Yes”
enter your answer here

[ZE_IM_wann] When did you receive these tick vaccinations?
If you can not remember exactly what month you received this vaccination, please specify only the year. If possible, check your vaccination card.
▼ ZE_2_3 == “Yes”
☐ I do not know for all vaccinations and can not check it out.

	Month	Year
1st vaccination		
2nd vaccination		
3rd vaccination		
4th vaccination		
5th vaccination		
6th vaccination		
7th vaccination		
8th vaccination		
9th vaccination		
10th vaccination		

3: Prevention – why tick vaccination?

[ZE_2_6] Why did you opt for tick vaccination? (Please state the most important reason for the last vaccination.)
▼ ZE_2_3 == “Yes”
☐ Because I've traveled to a risk area.
☐ Because I was privately endangered at my place of residence to come into contact with ticks.
☐ Because I was professionally at risk of coming into contact with ticks.
☐ Another reason, namely:

[ZE_2_7] What risk area did you travel to?
▼ ZE_2_6 == “Yes”
enter your answer here

[ZE_2_8] How many times did you go there?
▼ ZE_2_6 == “Yes”
☐ Once
☐ Multiple times
☐ I have lived there for a while.

[ZE_2_9] Where can / could you possibly come into contact with ticks?
▼ ZE_2_6 == “at risk of coming into contact with ticks”
☐ In Lower Saxony
☐ In a risk area
☐ In a non-risk area outside Lower Saxony

[ZE_2_10] In which area do / did you come into contact with ticks?
▼ ZE_2_9 == “risk area”
enter your answer here

[ZE_2_11] How did you decide to opt for tick vaccination? (Please state the most important reason.)
▼ ZE_2_3 == “Yes”
☐ Because my physician / employer has recommended to me the vaccination.
☐ Because my family / friends / acquaintances have recommended to me the vaccination.
☐ Because I have informed myself.
☐ Other:

4: Tick bites

[ZE_2_12] Have you been bitten by a tick at least once in the last 6 months?
☐ Yes
☐ No

[ZE_2_13] In which country were you bitten?
(multiple choice possible)
▼ ZE_2_12 == “Yes”
☐ In Germay
☐ Abroad

[ZE_2_13DE]
(multiple choice possible)
▼ ZE_2_13 == “Germany”
☐ Baden-Württemberg
☐ Bavaria
☐ Berlin
☐ Brandenburg
☐ Bremen
☐ Hamburg
☐ Hesse
☐ Mecklenburg-Vorpommern
☐ Lower Saxony
☐ North Rhine-Westphalia
☐ Rhineland-Palatinate
☐ Saarland
☐ Saxony
☐ Saxony-Anhalt
☐ Schleswig-Holstein
☐ Thuringia

[ZE_2_13AUS]
(multiple choice possible)
▼ ZE_2_13 == “Abroad”
☐ Austria
☐ Poland
☐ Czech Republic
☐ Switzerland
☐ Baltic countries
☐ Other country:

[ZE_2_14] How did you remove the tick? (If you have been bitten more than once, please refer to the last time you were bitten.)
▼ ZE_2_12 == “Yes”
☐ I myself removed the tick (within 1 hour) after the discovery /
had it removed by a person (not medically trained).
☐ I myself removed the tick, but not immediately (more than an hour after the discovery) /
had it removed by a person (not medically trained).
☐ I visited a doctor to have the tick removed.

5: Seeking medical advice

[ZE_2_15] Have you ever seen a doctor because you were bitten by a tick or suspected a tick bite?
☐ Yes
☐ No

[ZE_2_16] How often has it happened so far that you have visited a doctor because you were bitten by a tick or suspected a tick bite?
▼ ZE_2_15 == “Yes”
enter your answer here

6: Last time doctor

[ZE_2_17] Please remember the last time that you have visited a doctor because you were bitten by a tick or suspected a tick bite: What was the main reason for visiting the doctor?
▼ ZE_2_15 == “Yes”
☐ I wanted to have the tick removed.
☐ I was unsure about having the tick properly removed.
☐ I was afraid of being infected with borreliosis.
☐ I was afraid of being infected with early summer meningoencephalitis (TBE).
☐ I felt ill without being able to say exactly why.
☐ Another reason, namely:

[ZE_2_18] Were there other reasons to visit the doctor, besides the above mentioned reason? (multiple choice possible)
▼ ZE_2_15 == “Yes”
☐ No, there were no other reasons.
☐ I wanted to have the tick removed.
☐ I was unsure about having the tick properly removed.
☐ I was afraid of being infected with borreliosis.
☐ I was afraid of being infected with early summer meningoencephalitis (TBE).
☐ I felt ill without being able to say exactly why.

7: Suspicion of borreliosis

[ZE_2_19] Did you have any symptoms of the disease that let you suspect to be infected with borreliosis?
▼ (ZE_2_17 == “I was afraid of being infected with borreliosis.”) OR (ZE_2_18 == “I was afraid of being infected with borreliosis.”)

☐ Yes
☐ No

[ZE_2_20] What were the symptoms?
(multiple choice possible)
▼ ZE_2_19 == “Yes”

☐ Erythema migrans: redness of the skin around the puncture site that has migrated over time
☐ Fever
☐ Fatigue
☐ Headache
☐ Swollen lymph nodes
☐ Burning pain
☐ Paralysis
☐ Numbness on the skin
☐ Visual or hearing impairment
☐ Arrhythmia
☐ Joint pain
☐ Other:

[ZE_2_21] Has your suspicion of borreliosis been confirmed by a doctor?
▼ (ZE_2_17 == “I was afraid of being infected with borreliosis.”) OR (ZE_2_18 == “I was afraid of being infected with borreliosis.”)

☐ Yes
☐ No

8: Diagnosis of borreliosis

In the previous questions, we asked if had a suspicion of borreliosis. In the following questions, we are concerned with the question of whether borreliosis has ever been diagnosed and how this diagnosis came about. The questions are therefore similar to the previous questions. Please answer the following questions anyway.

[ZE_2_22] Have you ever been diagnosed with borreliosis?
☐ Yes
☐ No

[ZE_2_23] When was borreliosis diagnosed?
▼ ZE_2_22 == “Yes”
☐ Immediately after a tick bite
☐ Within 4 weeks after ticking
☐ After more than 4 weeks after the tick bite
☐ I could not remember the tick bite.

[ZE_2_24] What symptoms have you had in relation to the diagnosed borreliosis? (multiple choice possible)
▼ ZE_2_22 == “Yes”
☐ Erythema migrans: redness of the skin around the puncture site that has migrated over time
☐ Fever
☐ Fatigue
☐ Headache
☐ Swollen lymph nodes
☐ Burning pain
☐ Paralysis
☐ Numbness on the skin
☐ Visual or hearing impairment
☐ Arrhythmia
☐ Joint pain
☐ Other:

[ZE_2_25] Has treatment of borreliosis been performed?
▼ ZE_2_22 == “Yes”
☐ Yes, I got an antibiotic for 7-14 days.
☐ Yes, I have had an antibiotic for more than four weeks.
☐ No, I have not received any antibiotics.

9: Suspicion of TBE

[ZE_2_26] Did you have any symptoms of the disease that let you suspect to be infected with TBE?
▼ (ZE_2_17 == “I was afraid of being infected with early summer meningoencephalitis (TBE).”) OR (ZE_2_18 == “I was afraid of being infected with early summer meningoencephalitis (TBE).”)
☐ Yes
☐ No

[ZE_2_27] What were the symptoms?
(multiple choice possible)
▼ ZE_2_26 == “Yes”
☐ Erythema migrans: redness of the skin around the puncture site that has migrated over time
☐ Fever
☐ Fatigue
☐ Headache
☐ Swollen lymph nodes
☐ Burning pain
☐ Paralysis
☐ Numbness on the skin
☐ Visual or hearing impairment
☐ Arrhythmia
☐ Joint pain
☐ Other:

[ZE_2_28] Has your suspicion of TBE disease been confirmed by a doctor?
▼ (ZE_2_17 == “I was afraid of being infected with early summer meningoencephalitis (TBE).”) OR (ZE_2_18 == “I was afraid of being infected with early summer meningoencephalitis (TBE).”)
☐ Yes
☐ No

10: Other disease

[ZE_2_29] Which symtoms did you have?
(multiple choice possible)
▼ (ZE_2_17 == “I felt ill without being able to say exactly why.”) OR (ZE_2_18 == “I felt ill without being able to say exactly why.”)

- ☐ Erythema migrans: redness of the skin around the puncture site that has migrated over time
- ☐ Fever
- ☐ Fatigue
- ☐ Headache
- ☐ Swollen lymph nodes
- ☐ Burning pain
- ☐ Paralysis
- ☐ Numbness on the skin
- ☐ Visual or hearing impairment
- ☐ Arrhythmia
- ☐ Joint pain
- ☐ Other:

[ZE_2_30] Has the doctor diagnosed a disease?
▼ (ZE_2_17 == “5”) OR (ZE_2_18_5 == “Yes”)

- ☐ No
- ☐ Yes (please specify):

N: Multi-drug resistant pathogens

We would like to ask you about multiresistant germs, also known as "hospital germs". By this we mean pathogens which are resistant to various antibiotics. If these germs cause illness, the affected persons must be treated with special antibiotics.

1: Start

[MRE1] Have you ever heard of multiresistant germs?
- ☐ Yes
- ☐ No

2: General

[MRE2] Where or by whom did you hear about multiresistant germs?

☐ TV	☐ Brochures / information booklets
☐ Radio	☐ Doctor
☐ Newspaper	☐ Hospital staff
☐ Family / friends	☐ Colleagues
☐ Internet	☐ Others, namely:

[MRE3] Do you consider the topic of multiresistant germs as important?

☐ Very important
☐ Quite important
☐ Mediocre important
☐ Little important
☐ Not important

[MRE4] Have you ever been diagnosed with a multiresistant germ?

☐ Yes
☐ No
☐ Don't know

[MRE5] Do you know someone who has ever been diagnosed with a multidirectional germ?

☐ Yes
☐ No

[MRE6] Who do you know that has ever been diagnosed with a multidirectional germ? (multiple choice possible)

☐ Family member
☐ Friend(s)
☐ Acquaintance(s)
☐ Someone else, namely:

[MRE7] Are you personally afraid of getting infected with multiresistant germs?

☐ Very
☐ Quite
☐ Mediocre
☐ Little
☐ Not

[MRE8] Are you afraid that a family member could get infected with a multiresistant germ?

☐ Very
☐ Quite
☐ Mediocre
☐ Little
☐ Not

[MRE9] Are you afraid of multiresistant germs as a whole social problem?

☐ Very
☐ Quite
☐ Mediocre
☐ Little
☐ Not

3: Opinion

In the following, we would like to gather your opinion on multiresistent germs.

[MRE10] One can only get infected with multiresistant germs in the hospital.
☐ Strongly agree
☐ Agree
☐ Disagree
☐ Strongly disagree
☐ Don’t know

[MRE11] Multiresistant germs can not be treated.
☐ Strongly agree
☐ Agree
☐ Disagree
☐ Strongly disagree
☐ Don’t know

[MRE12] If I stop taking an antibiotic immediately in case of improvement of the disease, I contribute to the prevention of multiresistant germs.
☐ Strongly agree
☐ Agree
☐ Disagree
☐ Strongly disagree
☐ Don’t know

[MRE13] As long as I have a multiresistant germ only on the skin or mucous membrane, it is not dangerous for me.
☐ Strongly agree
☐ Agree
☐ Disagree
☐ Strongly disagree
☐ Don’t know

4: Reasons

In the following, we would like to know what you think contributes to the spread of multiresistant germs.

[MRE14] What do you think is important for the spread of multiresistant germs?

	Very important	Quite important	Mediocre important	Little important	Not important	Don't know
Improper use of antibiotics in the population	☐	☐	☐	☐	☐	☐
Improper use of antibiotics in animal breeding	☐	☐	☐	☐	☐	☐
Lack of hygiene in the medical field in general	☐	☐	☐	☐	☐	☐
Lack of hands-on hospital staff	☐	☐	☐	☐	☐	☐
Lack of hand hygiene in the population	☐	☐	☐	☐	☐	☐
Lack of bedding in hospitals	☐	☐	☐	☐	☐	☐
Too little effective medication	☐	☐	☐	☐	☐	☐
Other cause, namely:	☐	☐	☐	☐	☐	☐

5: Animal breeding

The following questions deal with the topic of animal breeding.

[MRE15] Do you or a person who lives in your household have professional care with farm animals, e.g. as a farmer, veterinarian or in meat processing?

- ☐ Yes, myself
- ☐ Yes, another person who lives in my household
- ☐ No

[MRE16] Politicians are responsible for reducing the use of antibiotics in animal breeding.

- ☐ Strongly agree
- ☐ Agree
- ☐ Disagree
- ☐ Strongly disagree
- ☐ Don’t know

[MRE17] Farmers are responsible for reducing the use of antibiotics in animal breeding.

- ☐ Strongly agree
- ☐ Agree
- ☐ Disagree
- ☐ Strongly disagree
- ☐ Don’t know

[MRE18] Consumers are responsible for reducing the use of antibiotics in animal breeding.

- ☐ Strongly agree
- ☐ Agree
- ☐ Disagree
- ☐ Strongly disagree
- ☐ Don’t know

[MRE19] I am willing to spend more money on meat (comparable to the costs of organic products) if this leads to a reduced use of antibiotics.

- ☐ Strongly agree
- ☐ Agree
- ☐ Disagree
- ☐ Strongly disagree
- ☐ Don’t know

6: Dissemination and responsibility

The following three questions address the issue of the spread of antibiotic resistance and responsibility in the health system.

[MRE20] Everyone is responsible for reducing the spread of multiresistant germs through the correct use of antibiotics.

☐ Strongly agree
☐ Agree
☐ Disagree
☐ Strongly disagree
☐ Don’t know

[MRE21] Doctors and nursing staff are responsible for reducing the spread of multiresistant germs in the health system.

☐ Strongly agree
☐ Agree
☐ Disagree
☐ Strongly disagree
☐ Don’t know

[MRE22] Politicians are responsible for reducing the spread of multiresistant germs in the health system.

☐ Strongly agree
☐ Agree
☐ Disagree
☐ Strongly disagree
☐ Don’t know

Two case studies follow to conclude the questionnaire.

7: Case study 1

[MRE23] Your neighbor, a single senior, needs some help and you have been buying groceries for him for a few months. After a hospital stay, he tells you that he has been diagnosed with a hospital germ. How would you behave?

	Strongly agree	Agree	Disagree	Strongly disagree
I behave exactly as before.	☐	☐	☐	☐
I wash my hands thoroughly after the visit.	☐	☐	☐	☐
I disinfect my hands after the visit.	☐	☐	☐	☐
I change my clothes after visiting.	☐	☐	☐	☐
My children are no longer allowed to visit the neighbor.	☐	☐	☐	☐
I now put the groceries in the hallway and avoid contact.	☐	☐	☐	☐
I now completely avoid the neighbors.	☐	☐	☐	☐
I'm afraid of being infected.	☐	☐	☐	☐

8: Case study 2

[MRE24] Your work colleague, with whom you share the office and sometimes use the same items, tells you after a hospital stay that she has been infected with a hospital germ. How would you behave?

	Strongly agree	Agree	Disagree	Strongly disagree
I behave exactly as before.	☐	☐	☐	☐
I often wash my hands.	☐	☐	☐	☐
I often disinfect my hands.	☐	☐	☐	☐
I change my clothes after work.	☐	☐	☐	☐
I avoid body contact with her.	☐	☐	☐	☐
I avoid contact with materials that are touched together.	☐	☐	☐	☐
I ask my boss to move to another office / workspace.	☐	☐	☐	☐
I'm afraid of being infected.	☐	☐	☐	☐

O: Fair allocation of scarce medical resources

In medicine, medical resources may not be available to all who need them. One example is organ transplantation. Even in the case of a flu pandemic, the vaccine can become scarce. The purpose of this questionnaire is to examine how scarce medical resources should be distributed. In the following questionnaire, there are no "right" or "wrong" answers. We are much more interested in your personal opinion about the distribution of medical resources.

1: Randomisation

[FM_Szenario]
random number (1 or 2)

[FM_Mortalitaet]
random number (0 or 1)

[FM_Info]
random number (0 or 1 or 2)

[FM_Fragestellung]
random number (0 or 1)

2: Distribution of medical services

Please read the following statements and state the degree of your approval.

[FM_Prinzip] In the distribution of medical resources (for example, therapy places, hospital beds, etc.), I feel it is fair, …

	Strongly disagree				Strongly agree
... if the resources are distributed according to the principle "first come, first served".	☐	☐	☐	☐	☐
... when younger people are preferred to older ones.	☐	☐	☐	☐	☐
... if the costs for beauty treatments (to comply with beauty ideals) are taken over by the health insurance company.	☐	☐	☐	☐	☐
... when ill persons receive the treatments they need.	☐	☐	☐	☐	☐
... if the costs for accident-related beauty procedures are taken over by the health insurance company.	☐	☐	☐	☐	☐
... if those receive the better services that pay more (for example, supplementary insurance or cost sharing).	☐	☐	☐	☐	☐
... if all get the services they want to have.	☐	☐	☐	☐	☐
... when people get less benefits that have an unhealthy lifestyle.	☐	☐	☐	☐	☐
... if the services are distributed according to the random principle.	☐	☐	☐	☐	☐
... if the general health of the patient (i.e. the medical prognosis) is taken into account.	☐	☐	☐	☐	☐

3: Distribution problem (alternative 1)

▼ FM_Szenario == "1"
The following situation is invented. However, it shows similarities with processes in reality. In a city, a sexually transmitted disease has spread for 20 years. This disease leads to death within if (FM_Mortalitaet == "1", "15 years", "5 years"). Currently every 50th inhabitant of the city is ill. The inhabitants differ in how often they change sexual partners and how often they have several sexual partners at the same time: approximately every 5th inhabitant belongs to the group with frequently changing partners or several partners at the same time. The remaining 80% of the population rarely change sexual partners. Younger residents of this city are changing their partners more often than older ones. A vaccine is now available for the first time. This protects reliably against contamination, already diseased persons are not cured by the vaccination however. The vaccine stock is currently not sufficient for all residents of the city. The vaccination can be distributed according to different criteria.

4: Distribution problem (alternative 1)

▼ (FM_Szenario == "1") AND ((FM_Info == "1") OR (FM_Info == "2"))
Without vaccination if (FM_Info == "1", "10,000", "20,000") inhabitants of the city would die of the disease. Researchers have estimated how much the number of deaths can be reduced depending on how the vaccination is distributed.

▼ FM_Info == "1"

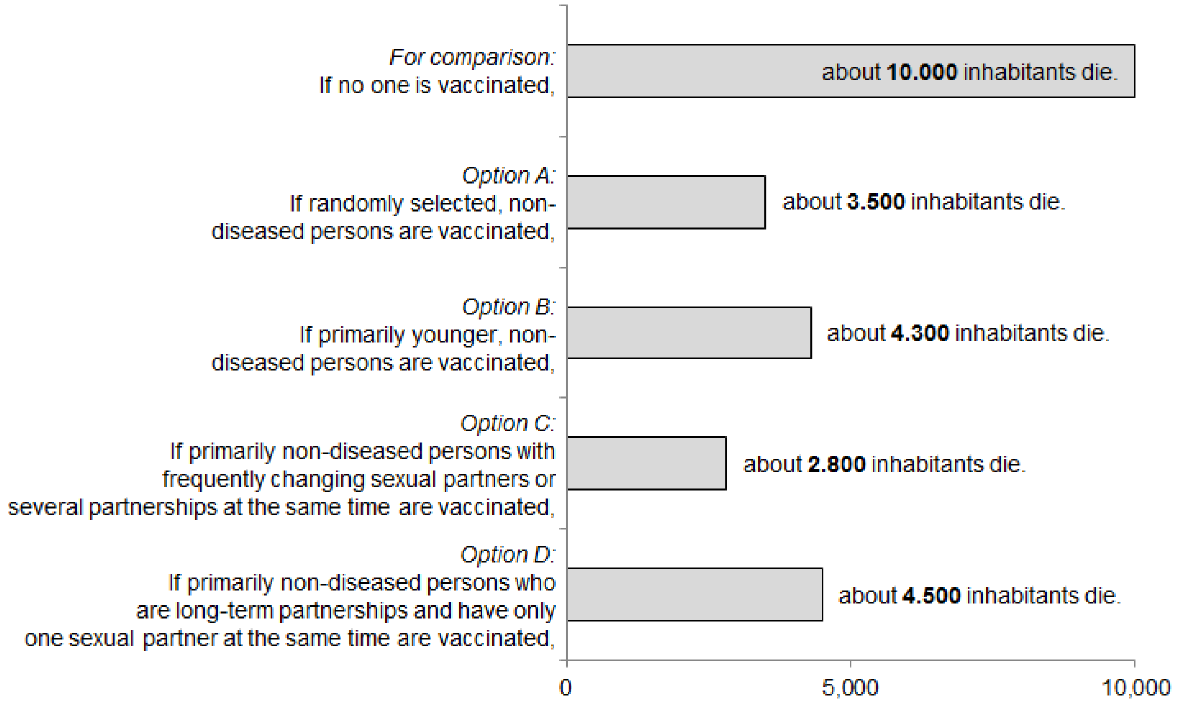

▼ FM_Info == "2"

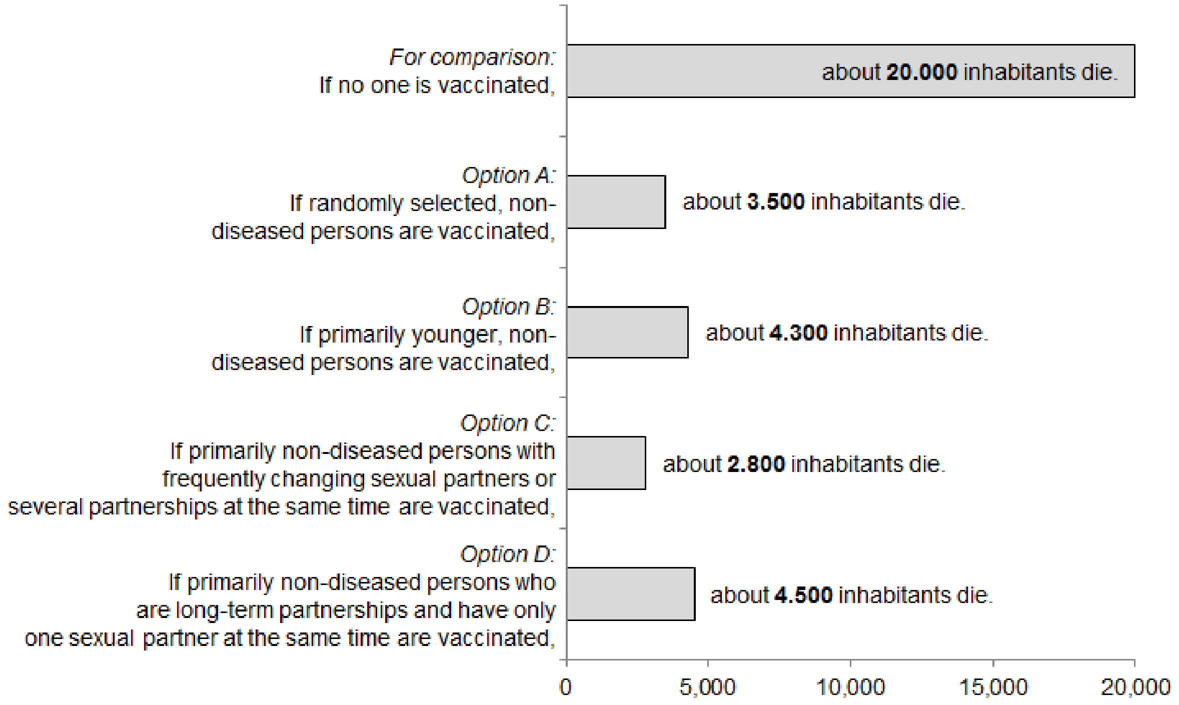

3: Distribution problem (alternative 2)

▼ FM_Szenario == "2"
The following situation is invented. However, it shows similarities with processes in reality. In a city, a sexually transmitted disease has spread for 20 years. This disease leads to death within if (FM_Mortalitaet == "1", "15 years", "5 years"). Currently every 50th inhabitant of the city is ill. The inhabitants differ in how often they change sexual partners and how often they have several sexual partners at the same time: approximately every 5th inhabitant belongs to the group with frequently changing partners or several partners at the same time. The remaining 80% of the population rarely change sexual partners. Younger residents of this city are changing their partners more often than older ones. A treatment option is now available for the first time. A ill person who is treated can become as old as a healthy person. The treatment protects others from infection. The treatment is very expensive. Therefore, it is not possible to treat all persons who have been infected. The treatment can be distributed according to different criteria.

4: Distribution problem (alternative 2)

▼ (FM_Szenario == "2") AND ((FM_Info == "1") OR (FM_Info == "2"))
Without treatment, if (FM_Info == "1", "10,000", "20,000") residents of the city would die from the disease. Researchers have estimated how much the number of deaths can be reduced depending on how the treatment is distributed.

▼ FM_Info == "1"

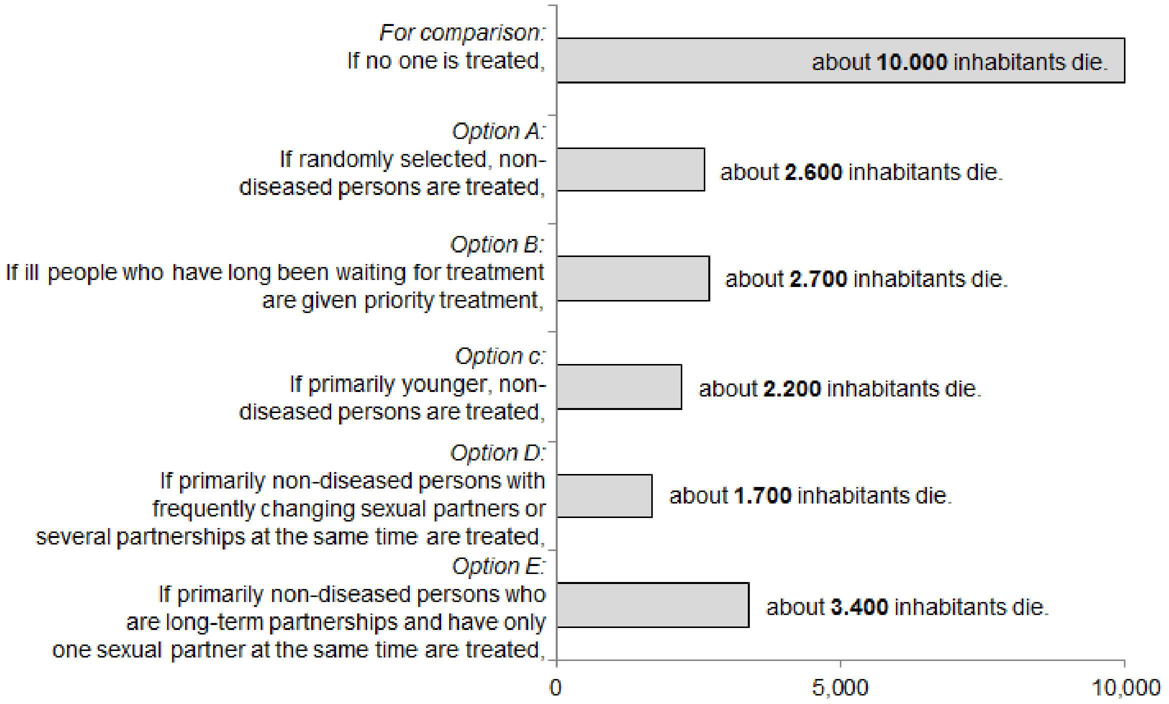

▼ FM_Info == "2"

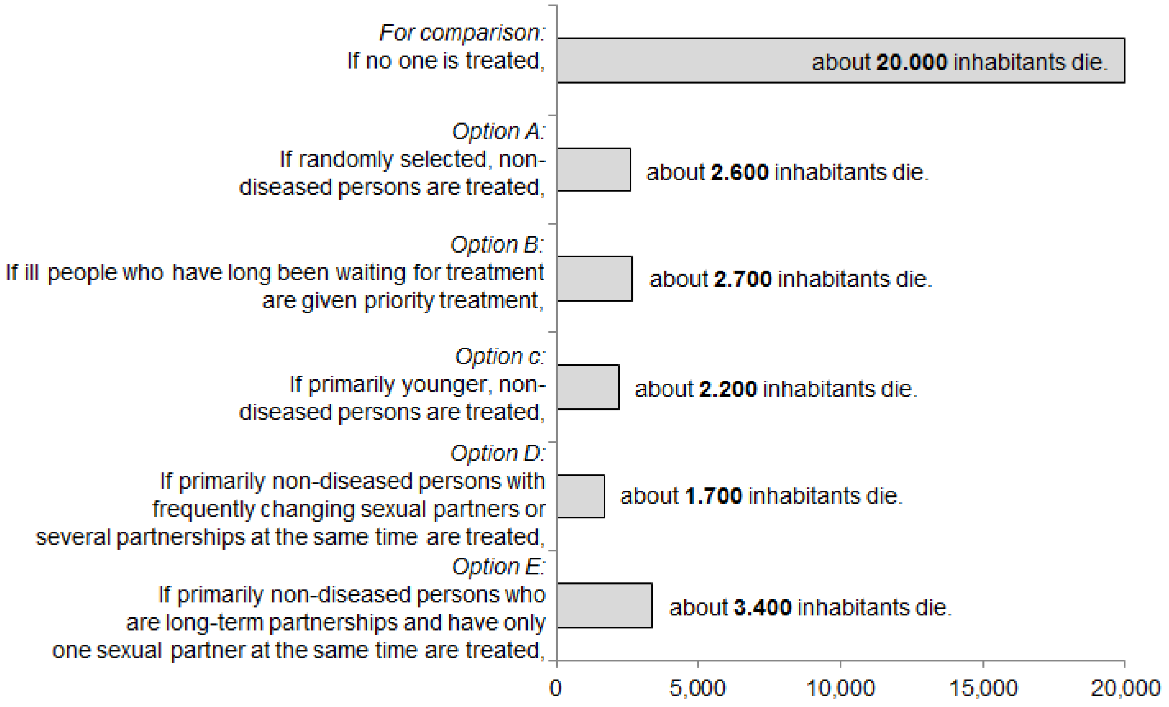

5: Question about distribution problem

[FM_Impfung_choice] if(FM_Fragestellung == "1", "How do you think the vaccinations should be distributed in the described situation?", "Which of the following distribution rules do you think is the fairest in the situation described?")
▼ FM_Szenario == "1"
☐ Option A: Randomly selected, non-diseased persons are vaccinated.
☐ Option B: Younger, non-diseased persons are primarily vaccinated.
☐ Option C: Priority is given to non-diseased individuals who often have changing sexual partners or several partnerships at the same time.
☐ Option D: Priority is given to non-diseased persons who have long-lasting partnerships and have only one sexual partner at the same time.
☐ I cannot decide.

[FM_Impfung_reason] if(FM_Impfung_choice == "90"," Why are you unable to decide? ", If (FM_Fragestellung ==" 1 "," Why did you choose this distribution of vaccination? "," Why do you consider the distribution rule you have chosen as the fairest? ")
Please justify your choice with 1 to 5 short sentences. Your answers are very important to the study.
enter your answer here

[FM_Behandlung_choice] if(FM_Fragestellung == "1", " How, in your opinion, should the treatment be distributed in the described situation? "," Which of the following distribution rules do you think is the fairest in the situation described? ")
▼ FM_Szenario == "2"
☐ Option A: Randomy selected ill persons are treated.
☐ Option B: Priority is given to ill people who have been waiting for treatment for a long time.
☐ Option C: Most younger, ill people are treated.
☐ Option D: Priority is given to ill-treated persons who frequently have changing sexual partners or several partnerships at the same time.
☐ Option E: Mainly ill people are treated who have long-lasting partnerships and have only one sexual partner at the same time.
☐ I cannot decide.

[FM_Behandlung_reason] if(FM_Impfung_choice == "90", "Why are you unable to decide?", If (FM_Fragestellung == "1", "Why did you choose this distribution of treatment?", "Why do you consider the distribution rule you have chosen as the fairest?")
Please justify your choice with 1 to 5 short sentences. Your answers are very important to the study.
enter your answer here

P: Frequency of infections during the winter season & influenza vaccination (season 2015/16)

In this questionnaire, we are interested in whether you had an infection of the respiratory tract in the past months and whether you were vaccinated against influenza in the autumn / winter season 2015/16.

1: Signs and symptoms

[IN1_2016] How often have you had an infection of the airways (for example, cold, flu, or otitis media) in the past 12 months?

☐ Not at all
☐ Once
☐ Twice
☐ Three to four times
☐ More than four times
☐ Don’t know

[GR_1] Since September 2014, have you had an illness with high fever (body temperature above 38° C) lasting several days?

☐ Yes
☐ No
☐ I’m not sure

[GR_2] How many days did you have malaise?
▼ GR_1 != “No”
enter your answer here

[GR_3] How many days did you have high fever (body temperature above 38° C)?
▼ GR_1 != “No”
enter your answer here

[GR_4] Did you have other symptoms besides fever?
▼ GR_1 != “No”

	Yes	No	Don’t know
[1] Throat pain	☐	☐	☐
[2] Dry cough	☐	☐	☐
[3] Muscle pain	☐	☐	☐
[4] Joint pain	☐	☐	☐
[5] Back pain	☐	☐	☐
[6] Headaches	☐	☐	☐
[7] Feeling of weakness	☐	☐	☐

[GR_4_8] Other symptoms:
▼ GR_1 != “No”
enter your answer here

2: Seeking medical advice

[GR_5] Did you see a doctor because of this illness?
▼ GR_1 != “No”
☐ Yes
☐ No

[GR_6] Did the doctor take a sample to diagnose a flu?
▼ (GR_5 == “Yes”)
☐ Yes
☐ No

[GR_7] Did the doctor diagnose a real flu or other disease, e.g. pneumonia, otitis media, or similar?
▼ (GR_1 != “No”) & (GR_5 == “Yes”)
☐ The doctor has not made any diagnosis.
☐ The doctor has diagnosed a real flu.
☐ The doctor has diagnosed another illness, namely:

3: Characteristics of the disease

[GR_8] How many days were you so ill that you had to stay in bed or could not do your normal everyday life (housework, hobbies etc.)?
▼ GR_1 != “No”
enter your answer here

[GR_9] How many days did you report ill and could not pursue your profession?
▼ GR_1 != “No”
☐ Does not apply to me
☐ Days:

[GR_10] How many of these {GR_9_other} days were you on ill leave?
▼ (GR_9 == “-oth-”)
☐ Does not apply to me
☐ Days:

[GR_11] Do you think that this disease might have been the real flu?
☐ Yes
☐ No
☐ I’m not sure

[GR_11_comment_1] Why do you think it was a real flu?
▼ GR_11 == “Yes”
enter your answer here

[GR_11_comment_0] Why do you think it was not a real flu?
▼ GR_11 == “No”
enter your answer here

4: Household

[P2_hh_single] Do you live alone in your household?
If you live in several households (e.g. weekend commuters), please refer to the household where you spend most of your time.
☐ Yes
☐ No

[P2_hh_p] How many people, including yourself, are living in your household?
A household includes all persons who live and work together here. Please also think of all children living in the household.
▼ P2_hh_single == “No”
enter your answer here

[P2_hh_age1] How old are the members of your household (including yourself)?
Please start with the youngest members. For children under one year, please enter "0".
▼ P2_hh_single == “No”
[1] Age (in years)
[2] Age (in years)
[3] Age (in years)
[4] Age (in years)
[5] Age (in years)
[6] Age (in years)
[7] Age (in years)
[8] Age (in years)
[9] Age (in years)
[10] Age (in years)

[GR_12] Are there among those household members people who are particularly vulnerable to flu, e.g. elderly or chronically ill persons?
▼ P2_hh_single == “No”
☐ No
☐ I am not sure
☐ Yes, number of persons:

5: Household II

[GR_13] Since September 2014, did someone from your household (not counting you) have an illness with high fever (body temperature above 38° C) lasting several days?
▼ P2_hh_single == “No”
☐ Yes
☐ No
☐ I’m not sure

[GR_14] How many persons from your household (not counting you) have had an illness with high fever (body temperature above 38° C) lasting several days?
▼ GR_13 == “Yes”
enter your answer here

[GR_15] Do you think that this disease might have been the real flu?
▼ GR_13 == “Yes”
☐ Yes
☐ No
☐ I’m not sure

[GR_15_comment_1] Why do you think it was a real flu?
▼ GR_15 == “Yes”
enter your answer here

[GR_15_comment_0] Why do you think it was not a real flu?
▼ GR_15 == “No”
enter your answer here

[GR_16] If you think of your infection with high fever, were the other ill person(s) in your household ill before, at the same time or after yourself?
With this question we would like to find out whether there was any contagion within your household. Please tick all that apply.
(multiple choice possible)
▼ (GR_1 == “Yes”) & (GR_13 == “Yes”)
☐ In my household, one or more people were ill within 2 weeks before myself.
☐ In my household, one or more people were ill within two weeks after myself.
☐ In my household, one or more people were ill with me at the same time.
☐ In my household, one or more people were ill at another time, that is, not within 2 weeks before or after my own illness.
☐ I don’t know when the other person (s) in my household was ill.

6: Influenza vaccination

[GR_17] Have you been vaccinated against influenza since August 2014?
☐ Yes
☐ No
☐ I’m not sure

[GR_18] When did you get vaccinated against flu?
▼ GR_17 == “Yes”
enter your answer here

[GR_19] Where was the influenza vaccination performed?
▼ GR_17 == “Yes”
☐ General practioner
☐ Company doctor
☐ Gynecologist
☐ Pediatrician
☐ Somewhere else

[GR_20] Was this influenza vaccination recorded in your vaccination card?
▼ GR_17 == “Yes”
☐ I don’t have a vaccination card.
☐ Yes
☐ No
☐ Don’t know

[GR_21] Did you have to pay for the influenza vaccination?
▼ GR_17 == “Yes”
☐ Yes
☐ No
☐ Don’t know

[GR_21_Zahlung] How much did you have to pay?
▼ GR_21 == “Yes”
enter your answer here

[GR_22] Would you have been vaccinated against influenza if you had to pay for the vaccination? (The vaccination costs about 20 euros.)
▼ GR_21 == “No”
☐ Yes
☐ No
☐ Don’t know

[GR_23] After the influenza vaccination, did you have any complaints that you have associated with vaccination?
▼ GR_17 == “Yes”
☐ No
☐ Yes (please specify):

7: Influenza vaccination II

[GR_24] Did anyone give you the advice or recommendation to get vaccinated against influenza?
☐ Yes
☐ No
☐ Don't know

[GR_25] Who gave you the advice or recommendation to get vaccinated against flu?
(multiple choice possible)
▼ GR_24 == "Yes"
☐ General practioner
☐ Gynecologist
☐ Pediatrician
☐ Family
☐ Someone else, namely:

[GR_26] Was the fact that someone gave you the advice or recommendation to get vaccinated against influenza is very important for your decision to get vaccinated?
▼ (GR_17 == "Yes") & (GR_24 == "Yes")
☐ Yes
☐ No

[GR_27] Has your company / employer recommended the vaccination, e.g. email, notice on the bulletin board or similar?
☐ Yes
☐ No
☐ Does not apply to me

[GR_28] Was the fact that your company / employer recommended the vaccination is very important for your decision to get vaccinated?
▼ (GR_17 == "Yes") & (GR_27 == "Yes")
☐ Yes
☐ No

8: Influenza vaccination III

[GR_29] Do you work in the medical field and have patient contact?

☐ Yes

☐ No

[GR_30] Was the fact that you have patient contact very important for your decision to get vaccinated?

▼ (GR_17 == “Yes”) & (GR_29 == “Yes”)

☐ Yes

☐ No

[GR_31] Was the fact that your household is home to a person for whom a flu would be particularly harmful, very important for your decision to get vaccinated?

▼ (GR_17 == “Yes”) & (GR_12 == “Yes”)

☐ Yes

☐ No

9: Reason for influenza vaccination

[GR_32] Did you have the impression that in the media the influenza wave was shown as particularly strong in winter 2014/2015?

☐ Yes

☐ No

[GR_33] Was the fact that in the media the flu wave in the winter 2014/2015 was shown as particularly strong, very important for your decision to get vaccinated?

▼ (GR_17 == "Yes") & (GR_32 == "Yes")

☐ Yes

☐ No

[GR_34] Was the fact that you did not want to get yourself influenza very important for your decision to get vaccinated?

▼ GR_17 == "Yes"

☐ Yes

☐ No

[GR_35] Please summarize briefly why you have been vaccinated:

▼ GR_17 == "Yes"

enter your answer here

10: Statements about influenza vaccination

How do you assess the following two statements?
[GR_36] The information about the influenza vaccination, which I receive through the media, allows me to make a decision on whether to vaccinate myself or not.

- ☐ Applies
- ☐ Rather applies
- ☐ I have not received any such information through the media.
- ☐ Does rather not apply
- ☐ Does not apply

[GR_37] The information about the influenza vaccination, which I receive through the media, is clear to me.

- ☐ Applies
- ☐ Rather applies
- ☐ I have not received any such information through the media.
- ☐ Does rather not apply
- ☐ Does not apply

11: Recommendation influenza vaccination

[GR_38] For which groups of persons is the influenza vaccination recommended by the Standing Immunization Commission (STIKO) at the Robert Koch Institute?

	Yes	No	Don't know
[1] For the elderly over 60 years.	☐	☐	☐
[2] For all adults over 18 years.	☐	☐	☐
[3] For persons with increased health risk due to chronic condition.	☐	☐	☐
[4] For children who visit a children's day-care center.	☐	☐	☐
[5] For persons who work in the medical field and have contact with the patient.	☐	☐	☐
[6] For women who are pregnant during the winter half year.	☐	☐	☐

12: Outlook influenza vaccination

[GR_39_f] Do you belong to one of the following groups?
(multiple choice possible)

☐ I am over 60 years old.
☐ I have a chronic condition.
☐ I have a child / children visiting a children's day.
☐ I gave birth to a child between September 2014 and March 2015.
☐ I am currently pregnant.
☐ I don’t belong to one of these groups.

[GR_40] Are you planning to get vaccinated against influenza in the autumn / winter season 2016/17?

☐ Yes
☐ No
☐ Not decided yet

[GR_41] Have your experiences with the influenza vaccination in the 2015/16 season affected your decision for the autumn / winter season 2016/17?
▼ GR_17 == “Yes”

☐ Yes
☐ No
☐ Don’t know

[GR_42] How did your experiences with the influenza vaccination in the 2015/16 season influence your decision for the autumn / winter season 2016/17?
▼ GR_41 == “Yes”
enter your answer here

[GR_remark] Is there anything else you would like to tell us about this questionnaire?
enter your answer here
