# Supplementary material for: Factors associated with attrition in a longitudinal online study: results from the HaBIDS panel
Source: BMC Med Res Methodol. 2017 Aug 31;17:132. doi: 10.1186/s12874-017-0408-3 (PMC5580321; doi:10.1186/s12874-017-0408-3)

Article title:

Factors associated with attrition in a longitudinal online study: results from the HaBIDS panel

Authors:

Nicole Rübsamen; Manas K Akmatov; Stefanie Castell; André Karch; Rafael T. Mikolajczyk

Journal:

BMC Medical Research Methodology

Additional file 3 – Flow diagram of numbers of individuals at each stage of study

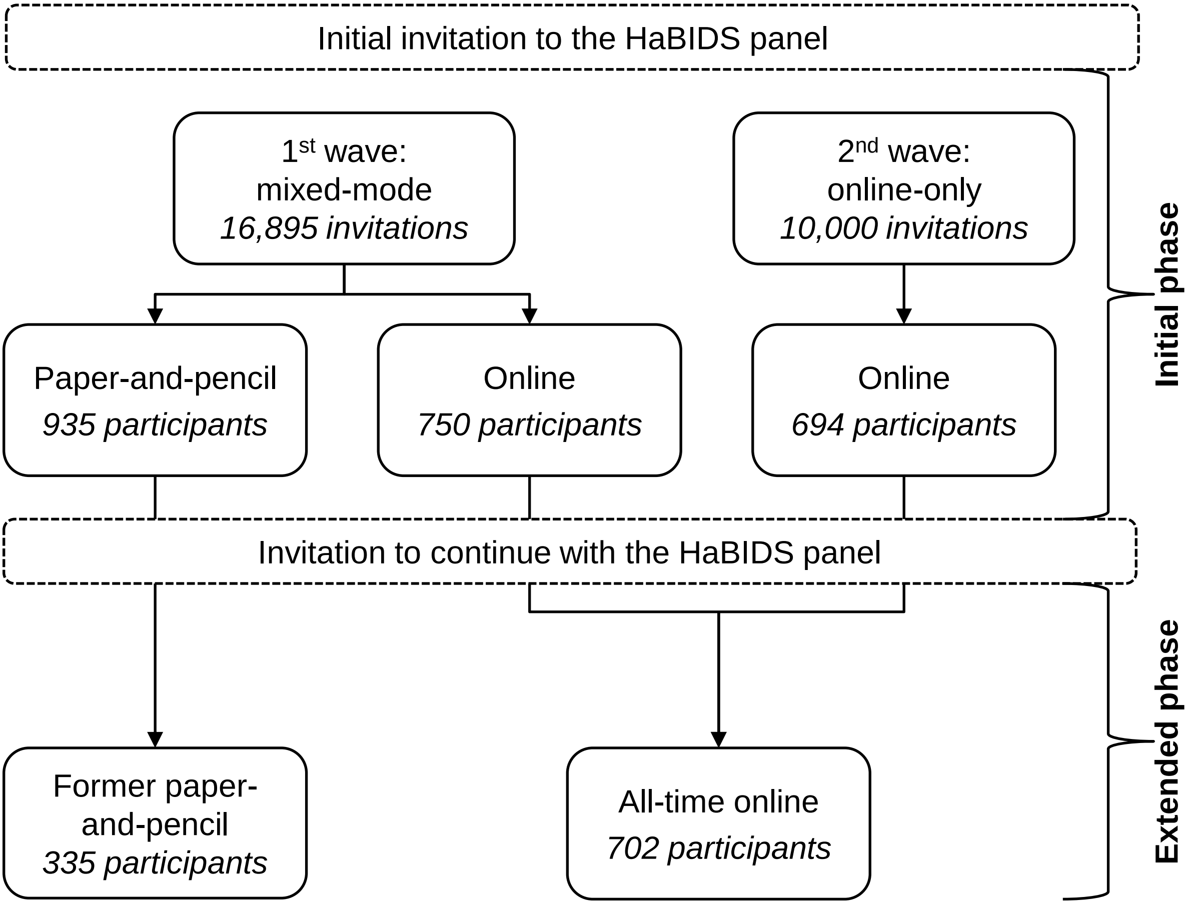

Supplement: Supplementary file 3 — Flow diagram of numbers of individuals at each stage of study. (PDF 137 kb) [file 12874_2017_408_MOESM3_ESM.pdf]
