# Supplementary material for: Factors associated with attrition in a longitudinal online study: results from the HaBIDS panel
Source: BMC Med Res Methodol. 2017 Aug 31;17:132. doi: 10.1186/s12874-017-0408-3 (PMC5580321; doi:10.1186/s12874-017-0408-3)
Supplement: Supplementary file 4 — Definition of discontinuation for each online questionnaire. (PDF 237 kb) [file 12874_2017_408_MOESM4_ESM.pdf]

Article title:

Factors associated with attrition in a longitudinal online study: results from the HaBIDS panel

Authors:
Nicole Rübsamen; Manas K Akmatov; Stefanie Castell; André Karch; Rafael T. Mikolajczyk

Journal:
BMC Medical Research Methodology

Additional file 4 – Definition of discontinuation for each online questionnaire

		**Questionnaire filled in (x = yes, o = no)**															
**Discontinuation at questionnaire**		A	B	C	D	E	F	G	H	I	J	K	L	M	N	O	P
	**Initial phase**																
	A	Start with all participants who filled in questionnaire A															
	B	x	o	o	o	o	o	o	o	o	o	o					
	C	x	x	o	o	o	o	o	o	o	o	o					
	D	x	x	x	o	o	o	o	o	o	o	o					
	E	x	x	x	x	o	o	o	o	o	o	o					
	F	x	x	x	x	x	o	o	o	o	o	o					
	G	x	x	x	x	x	x	o	o	o	o	o					
	H	x	x	x	x	x	x	x	o	o	o	o					
	I	x	x	x	x	x	x	x	x	o	o	o					
	J	x	x	x	x	x	x	x	x	x	o	o					
	K	By definition, discontinuation cannot be evaluated at questionnaire K															
													**Extended phase**				
	L	Start with all participants who filled in questionnaire L															
	M												x	o	o	o	o
	N												x	x	o	o	o
	O												x	x	x	o	o
	P	By definition, discontinuation cannot be evaluated at questionnaire P															
