# Supplementary material for: Factors associated with attrition in a longitudinal online study: results from the HaBIDS panel
Source: BMC Med Res Methodol. 2017 Aug 31;17:132. doi: 10.1186/s12874-017-0408-3 (PMC5580321; doi:10.1186/s12874-017-0408-3)
Supplement: Supplementary file 5 — Comparison of the study population’s composition with the target population’s (inhabitants of Lower Saxony between 15 and 69 years of age) composition. Data about the target population are taken from the Census 2011 by the Federal Statistical Office of Germany [19]. (PDF 322 kb) [file 12874_2017_408_MOESM5_ESM.pdf]

Article title:

Factors associated with attrition in a longitudinal online study: results from the HaBIDS panel

Authors:

Nicole Rübsamen; Manas K Akmatov; Stefanie Castell; André Karch; Rafael T. Mikolajczyk

Journal:

BMC Medical Research Methodology

Additional file 5 – Comparison of the study population's composition with the target population's (inhabitants of Lower Saxony between 15 and 69 years of age) composition

* Still at upper secondary school

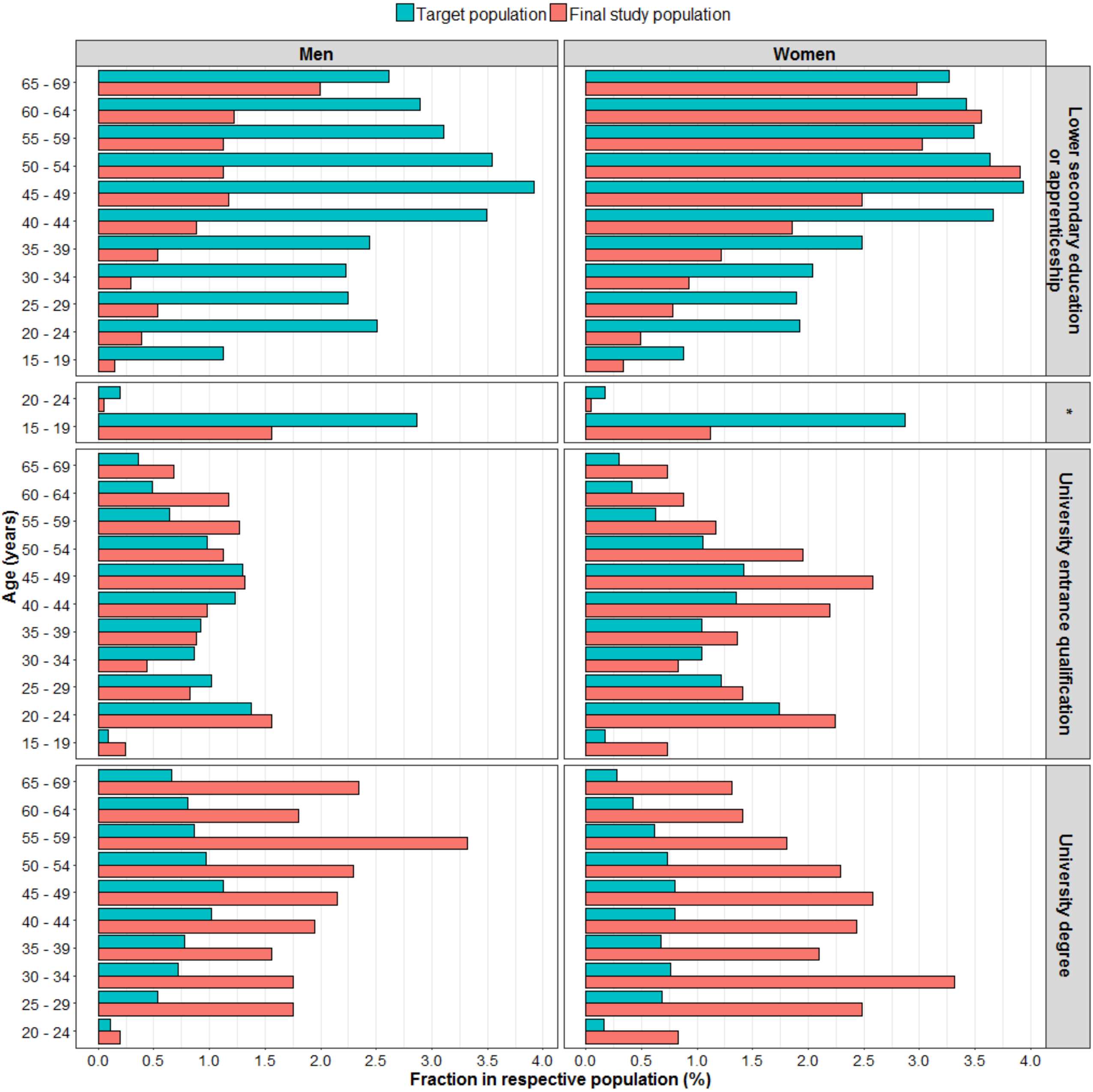

Composition of the target population is available from:

https://ergebnisse.zensus2011.de/#dynTable:statUnit=PERSON;absRel=ANZAHL;ags=03;agsAxis=X;yAxis=SCHULABS,BERUFABS_AUSF,GESCHLECHT,ALTER_05JG

The original data that were exported from the URL above can be found on the following pages.

Personen nach Alter (5er-Jahresgruppen), Höchster beruflicher Abschluss (ausführlich) und weitere Merkmale für Niedersachsen (Bundesland)

Hochrechnung aus der Haushaltsstichprobe

				Niedersachsen (Bundesland)
Insgesamt	Insgesamt	Insgesamt	Insgesamt	6 630 290
			Unter 5	/
			5 - 9	/
			10 - 14	/
			15 - 19	436 170
			20 - 24	444 920
			25 - 29	413 660
			30 - 34	415 880
			35 - 39	452 500
			40 - 44	626 700
			45 - 49	678 070
			50 - 54	590 980
			55 - 59	506 190
			60 - 64	459 030
			65 - 69	406 080
			70 - 74	475 500
			75 - 79	315 960
			80 - 84	226 210
			85 - 89	130 290
			90 und älter	52 140
		Männlich	Insgesamt	3 210 960
			Unter 5	/
			5 - 9	/
			10 - 14	/
			15 - 19	222 630
			20 - 24	227 640
			25 - 29	207 070
			30 - 34	206 760
			35 - 39	224 650
			40 - 44	311 410
			45 - 49	344 130
			50 - 54	297 650
			55 - 59	250 100
			60 - 64	227 130
			65 - 69	197 170
			70 - 74	221 490
			75 - 79	138 960
			80 - 84	85 330
			85 - 89	38 440
			90 und älter	10 410
		Weiblich	Insgesamt	3 419 330
			Unter 5	/
			5 - 9	/
			10 - 14	/
			15 - 19	213 550
			20 - 24	217 280
			25 - 29	206 590
			30 - 34	209 120
			35 - 39	227 850
			40 - 44	315 290
			45 - 49	333 940
			50 - 54	293 330

Noch: Personen nach Alter (5er-Jahresgruppen), Höchster beruflicher Abschluss (ausführlich) und weitere Merkmale für Niedersachsen (Bundesland)

			Niedersachsen (Bundesland)
		55 - 59	256 090
		60 - 64	231 900
		65 - 69	208 920
		70 - 74	254 010
		75 - 79	177 000
		80 - 84	140 890
		85 - 89	91 850
		90 und älter	41 720
Ohne beruflichen Abschluss	Insgesamt	Insgesamt	1 900 720
		Unter 5	/
		5 - 9	/
		10 - 14	/
		15 - 19	421 550
		20 - 24	240 940
		25 - 29	94 740
		30 - 34	76 070
		35 - 39	75 170
		40 - 44	97 120
		45 - 49	113 300
		50 - 54	107 230
		55 - 59	86 650
		60 - 64	79 240
		65 - 69	79 850
		70 - 74	132 590
		75 - 79	120 440
		80 - 84	95 120
		85 - 89	55 690
		90 und älter	25 010
	Männlich	Insgesamt	767 810
		Unter 5	/
		5 - 9	/
		10 - 14	/
		15 - 19	214 660
		20 - 24	121 800
		25 - 29	50 390
		30 - 34	36 740
		35 - 39	34 750
		40 - 44	42 970
		45 - 49	52 550
		50 - 54	45 540
		55 - 59	32 450
		60 - 64	24 840
		65 - 69	23 040
		70 - 74	33 690
		75 - 79	26 230
		80 - 84	17 650
		85 - 89	8 360
		90 und älter	2 160
	Weiblich	Insgesamt	1 132 910
		Unter 5	/
		5 - 9	/
		10 - 14	/
		15 - 19	206 890

Noch: Personen nach Alter (5er-Jahresgruppen), Höchster beruflicher Abschluss (ausführlich) und weitere Merkmale für Niedersachsen (Bundesland)

			Niedersachsen (Bundesland)
		20 - 24	119 130
		25 - 29	44 350
		30 - 34	39 330
		35 - 39	40 420
		40 - 44	54 150
		45 - 49	60 750
		50 - 54	61 690
		55 - 59	54 200
		60 - 64	54 400
		65 - 69	56 820
		70 - 74	98 900
		75 - 79	94 210
		80 - 84	77 470
		85 - 89	47 340
		90 und älter	22 850
Lehre, Berufsausbildung im dualen System	Insgesamt	Insgesamt	3 134 140
		Unter 5	/
		5 - 9	/
		10 - 14	/
		15 - 19	12 110
		20 - 24	160 320
		25 - 29	205 470
		30 - 34	203 260
		35 - 39	234 530
		40 - 44	336 150
		45 - 49	355 270
		50 - 54	303 030
		55 - 59	272 750
		60 - 64	252 930
		65 - 69	225 540
		70 - 74	249 010
		75 - 79	145 410
		80 - 84	99 300
		85 - 89	57 560
		90 und älter	21 510
	Männlich	Insgesamt	1 576 330
		Unter 5	/
		5 - 9	/
		10 - 14	/
		15 - 19	7 280
		20 - 24	90 090
		25 - 29	109 790
		30 - 34	106 090
		35 - 39	117 250
		40 - 44	164 130
		45 - 49	176 380
		50 - 54	154 910
		55 - 59	136 090
		60 - 64	124 230
		65 - 69	108 730
		70 - 74	126 350
		75 - 79	79 380
		80 - 84	48 420

Noch: Personen nach Alter (5er-Jahresgruppen), Höchster beruflicher Abschluss (ausführlich) und weitere Merkmale für Niedersachsen (Bundesland)

			Niedersachsen (Bundesland)
		85 - 89	21 380
		90 und älter	5 840
	Weiblich	Insgesamt	1 557 820
		Unter 5	/
		5 - 9	/
		10 - 14	/
		15 - 19	4 830
		20 - 24	70 240
		25 - 29	95 680
		30 - 34	97 170
		35 - 39	117 270
		40 - 44	172 020
		45 - 49	178 890
		50 - 54	148 130
		55 - 59	136 660
		60 - 64	128 700
		65 - 69	116 810
		70 - 74	122 670
		75 - 79	66 030
		80 - 84	50 880
		85 - 89	36 180
		90 und älter	15 660
Fachschulabschluss	Insgesamt	Insgesamt	688 650
		Unter 5	/
		5 - 9	/
		10 - 14	/
		15 - 19	2 510
		20 - 24	25 420
		25 - 29	41 460
		30 - 34	48 040
		35 - 39	55 420
		40 - 44	83 630
		45 - 49	93 990
		50 - 54	80 630
		55 - 59	59 990
		60 - 64	53 380
		65 - 69	44 500
		70 - 74	46 790
		75 - 79	25 550
		80 - 84	16 090
		85 - 89	8 400
		90 und älter	2 860
	Männlich	Insgesamt	347 500
		Unter 5	/
		5 - 9	/
		10 - 14	/
		15 - 19	680
		20 - 24	8 490
		25 - 29	15 630
		30 - 34	21 430
		35 - 39	25 830
		40 - 44	43 490
		45 - 49	48 060

Noch: Personen nach Alter (5er-Jahresgruppen), Höchster beruflicher Abschluss (ausführlich) und weitere Merkmale für Niedersachsen (Bundesland)

			Niedersachsen (Bundesland)
		50 - 54	40 190
		55 - 59	30 710
		60 - 64	30 710
		65 - 69	26 400
		70 - 74	27 970
		75 - 79	15 030
		80 - 84	8 650
		85 - 89	3 360
		90 und älter	870
	Weiblich	Insgesamt	341 150
		Unter 5	/
		5 - 9	/
		10 - 14	/
		15 - 19	1 830
		20 - 24	16 930
		25 - 29	25 830
		30 - 34	26 610
		35 - 39	29 590
		40 - 44	40 140
		45 - 49	45 930
		50 - 54	40 440
		55 - 59	29 280
		60 - 64	22 670
		65 - 69	18 110
		70 - 74	18 820
		75 - 79	10 520
		80 - 84	7 440
		85 - 89	5 040
		90 und älter	1 990
Abschluss einer Fachakademie oder Berufsakademie	Insgesamt	Insgesamt	82 130
		Unter 5	/
		5 - 9	/
		10 - 14	/
		15 - 19	/
		20 - 24	2 930
		25 - 29	5 970
		30 - 34	8 220
		35 - 39	8 750
		40 - 44	11 400
		45 - 49	10 850
		50 - 54	8 180
		55 - 59	6 450
		60 - 64	6 540
		65 - 69	4 810
		70 - 74	3 730
		75 - 79	1 940
		80 - 84	1 220
		85 - 89	880
		90 und älter	/
	Männlich	Insgesamt	43 970
		Unter 5	/
		5 - 9	/
		10 - 14	/

Noch: Personen nach Alter (5er-Jahresgruppen), Höchster beruflicher Abschluss (ausführlich) und weitere Merkmale für Niedersachsen (Bundesland)

			Niedersachsen (Bundesland)
		15 - 19	/
		20 - 24	1 290
		25 - 29	2 400
		30 - 34	3 720
		35 - 39	4 550
		40 - 44	5 820
		45 - 49	6 100
		50 - 54	4 580
		55 - 59	3 810
		60 - 64	3 640
		65 - 69	3 310
		70 - 74	2 370
		75 - 79	1 250
		80 - 84	650
		85 - 89	/
		90 und älter	/
	Weiblich	Insgesamt	38 160
		Unter 5	/
		5 - 9	/
		10 - 14	/
		15 - 19	/
		20 - 24	1 630
		25 - 29	3 570
		30 - 34	4 500
		35 - 39	4 200
		40 - 44	5 590
		45 - 49	4 750
		50 - 54	3 600
		55 - 59	2 640
		60 - 64	2 890
		65 - 69	1 500
		70 - 74	1 370
		75 - 79	690
		80 - 84	580
		85 - 89	470
		90 und älter	/
Fachhochschulabschluss	Insgesamt	Insgesamt	326 020
		Unter 5	/
		5 - 9	/
		10 - 14	/
		15 - 19	/
		20 - 24	5 620
		25 - 29	21 650
		30 - 34	30 050
		35 - 39	30 310
		40 - 44	40 420
		45 - 49	43 940
		50 - 54	39 020
		55 - 59	30 620
		60 - 64	24 970
		65 - 69	20 720
		70 - 74	18 950
		75 - 79	9 890

Noch: Personen nach Alter (5er-Jahresgruppen), Höchster beruflicher Abschluss (ausführlich) und weitere Merkmale für Niedersachsen (Bundesland)

			Niedersachsen (Bundesland)
		80 - 84	5 570
		85 - 89	3 340
		90 und älter	940
		Insgesamt	213 800
		Unter 5	/
		5 - 9	/
		10 - 14	/
		15 - 19	/
		20 - 24	2 520
		25 - 29	11 360
		30 - 34	16 840
		35 - 39	18 740
	Männlich	40 - 44	25 380
		45 - 49	28 720
		50 - 54	24 860
		55 - 59	19 550
		60 - 64	18 790
		65 - 69	16 220
		70 - 74	15 360
		75 - 79	8 110
		80 - 84	4 250
		85 - 89	2 430
		90 und älter	670
		Insgesamt	112 220
		Unter 5	/
		5 - 9	/
		10 - 14	/
		15 - 19	/
		20 - 24	3 100
		25 - 29	10 290
		30 - 34	13 210
		35 - 39	11 570
	Weiblich	40 - 44	15 040
		45 - 49	15 220
		50 - 54	14 160
		55 - 59	11 070
		60 - 64	6 180
		65 - 69	4 500
		70 - 74	3 590
		75 - 79	1 770
		80 - 84	1 330
		85 - 89	910
		90 und älter	/
		Insgesamt	431 870
		Unter 5	/
		5 - 9	/
		10 - 14	/
Hochschulabschluss	Insgesamt	15 - 19	/
		20 - 24	9 520
		25 - 29	43 010
		30 - 34	45 100
		35 - 39	40 990
		40 - 44	49 880

Noch: Personen nach Alter (5er-Jahresgruppen), Höchster beruflicher Abschluss (ausführlich) und weitere Merkmale für Niedersachsen (Bundesland)

			Niedersachsen (Bundesland)
		45 - 49	51 340
		50 - 54	44 700
		55 - 59	43 170
		60 - 64	36 700
		65 - 69	26 010
		70 - 74	19 900
		75 - 79	9 930
		80 - 84	6 790
		85 - 89	3 690
		90 und älter	1 160
		Insgesamt	215 510
		Unter 5	/
		5 - 9	/
		10 - 14	/
		15 - 19	/
		20 - 24	3 290
		25 - 29	17 020
		30 - 34	19 470
		35 - 39	19 260
	Männlich	40 - 44	24 200
		45 - 49	26 130
		50 - 54	21 990
		55 - 59	22 580
		60 - 64	20 650
		65 - 69	15 680
		70 - 74	12 030
		75 - 79	6 630
		80 - 84	4 020
		85 - 89	2 020
		90 und älter	550
		Insgesamt	216 370
		Unter 5	/
		5 - 9	/
		10 - 14	/
		15 - 19	/
		20 - 24	6 230
		25 - 29	25 980
		30 - 34	25 630
		35 - 39	21 730
	Weiblich	40 - 44	25 670
		45 - 49	25 210
		50 - 54	22 710
		55 - 59	20 600
		60 - 64	16 050
		65 - 69	10 330
		70 - 74	7 860
		75 - 79	3 300
		80 - 84	2 770
		85 - 89	1 680
		90 und älter	610
		Insgesamt	66 750
Promotion	Insgesamt	Unter 5	/
		5 - 9	/

Noch: Personen nach Alter (5er-Jahresgruppen), Höchster beruflicher Abschluss (ausführlich) und weitere Merkmale für Niedersachsen (Bundesland)

			Niedersachsen (Bundesland)
		10 - 14	/
		15 - 19	/
		20 - 24	/
		25 - 29	1 380
		30 - 34	5 140
		35 - 39	7 340
		40 - 44	8 110
		45 - 49	9 380
		50 - 54	8 180
		55 - 59	6 540
		60 - 64	5 280
		65 - 69	4 650
		70 - 74	4 520
		75 - 79	2 810
		80 - 84	2 120
		85 - 89	730
		90 und älter	390
	Männlich	Insgesamt	46 050
		Unter 5	/
		5 - 9	/
		10 - 14	/
		15 - 19	/
		20 - 24	/
		25 - 29	480
		30 - 34	2 470
		35 - 39	4 280
		40 - 44	5 420
		45 - 49	6 200
		50 - 54	5 590
		55 - 59	4 910
		60 - 64	4 270
		65 - 69	3 790
		70 - 74	3 710
		75 - 79	2 330
		80 - 84	1 690
		85 - 89	500
		90 und älter	/
	Weiblich	Insgesamt	20 700
		Unter 5	/
		5 - 9	/
		10 - 14	/
		15 - 19	/
		20 - 24	/
		25 - 29	900
		30 - 34	2 670
		35 - 39	3 070
		40 - 44	2 690
		45 - 49	3 180
		50 - 54	2 590
		55 - 59	1 630
		60 - 64	1 010
		65 - 69	860
		70 - 74	800

Noch: Personen nach Alter (5er-Jahresgruppen), Höchster beruflicher Abschluss (ausführlich) und weitere Merkmale für Niedersachsen (Bundesland)

				Niedersachsen (Bundesland)
			75 - 79	480
			80 - 84	430
			85 - 89	/
			90 und älter	/
1 Ohne oder noch kein Schulabschluss	Insgesamt	Insgesamt	Insgesamt	487 510
			Unter 5	/
			5 - 9	/
			10 - 14	/
			15 - 19	199 050
			20 - 24	19 720
			25 - 29	17 160
			30 - 34	19 460
			35 - 39	21 160
			40 - 44	25 370
			45 - 49	28 870
			50 - 54	26 000
			55 - 59	20 610
			60 - 64	18 640
			65 - 69	16 080
			70 - 74	23 000
			75 - 79	25 120
			80 - 84	16 860
			85 - 89	7 050
			90 und älter	3 330
		Männlich	Insgesamt	240 960
			Unter 5	/
			5 - 9	/
			10 - 14	/
			15 - 19	106 260
			20 - 24	11 240
			25 - 29	8 990
			30 - 34	10 180
			35 - 39	9 780
			40 - 44	12 720
			45 - 49	15 360
			50 - 54	13 720
			55 - 59	10 070
			60 - 64	8 650
			65 - 69	7 540
			70 - 74	9 770
			75 - 79	9 220
			80 - 84	5 390
			85 - 89	1 570
			90 und älter	500
		Weiblich	Insgesamt	246 550
			Unter 5	/
			5 - 9	/
			10 - 14	/
			15 - 19	92 790
			20 - 24	8 490
			25 - 29	8 170
			30 - 34	9 280
			35 - 39	11 390

Noch: Personen nach Alter (5er-Jahresgruppen), Höchster beruflicher Abschluss (ausführlich) und weitere Merkmale für Niedersachsen (Bundesland)

			Niedersachsen (Bundesland)
		40 - 44	12 650
		45 - 49	13 510
		50 - 54	12 270
		55 - 59	10 550
		60 - 64	9 990
		65 - 69	8 540
		70 - 74	13 230
		75 - 79	15 900
		80 - 84	11 470
		85 - 89	5 480
		90 und älter	2 840
		Insgesamt	439 850
		Unter 5	/
		5 - 9	/
		10 - 14	/
		15 - 19	198 560
		20 - 24	18 510
		25 - 29	15 380
		30 - 34	17 390
		35 - 39	18 610
	Insgesamt	40 - 44	22 280
		45 - 49	24 880
		50 - 54	22 070
		55 - 59	16 340
		60 - 64	13 820
		65 - 69	12 780
		70 - 74	18 260
		75 - 79	19 550
		80 - 84	12 630
		85 - 89	6 060
		90 und älter	2 730
Ohne beruflichen Abschluss		Insgesamt	211 180
		Unter 5	/
		5 - 9	/
		10 - 14	/
		15 - 19	105 990
		20 - 24	10 480
		25 - 29	7 740
		30 - 34	8 820
		35 - 39	8 010
	Männlich	40 - 44	10 590
		45 - 49	12 470
		50 - 54	10 900
		55 - 59	7 460
		60 - 64	5 560
		65 - 69	5 720
		70 - 74	6 830
		75 - 79	5 990
		80 - 84	3 040
		85 - 89	1 220
		90 und älter	360
	Weiblich	Insgesamt	228 670
		Unter 5	/

Noch: Personen nach Alter (5er-Jahresgruppen), Höchster beruflicher Abschluss (ausführlich) und weitere Merkmale für Niedersachsen (Bundesland)

			Niedersachsen (Bundesland)
		5 - 9	/
		10 - 14	/
		15 - 19	92 570
		20 - 24	8 030
		25 - 29	7 630
		30 - 34	8 570
		35 - 39	10 600
		40 - 44	11 690
		45 - 49	12 410
		50 - 54	11 170
		55 - 59	8 890
		60 - 64	8 260
		65 - 69	7 050
		70 - 74	11 430
		75 - 79	13 560
		80 - 84	9 590
		85 - 89	4 850
		90 und älter	2 380
		Insgesamt	42 030
		Unter 5	/
		5 - 9	/
		10 - 14	/
		15 - 19	/
		20 - 24	1 020
		25 - 29	1 530
		30 - 34	1 890
		35 - 39	2 260
	Insgesamt	40 - 44	2 700
		45 - 49	3 330
		50 - 54	3 360
		55 - 59	3 950
		60 - 64	4 210
		65 - 69	2 800
		70 - 74	4 280
Lehre, Berufsausbildung im dualen System		75 - 79	5 260
		80 - 84	3 730
		85 - 89	950
		90 und älter	480
		Insgesamt	26 120
		Unter 5	/
		5 - 9	/
		10 - 14	/
		15 - 19	/
		20 - 24	580
		25 - 29	1 070
	Männlich	30 - 34	1 200
		35 - 39	1 550
		40 - 44	1 850
		45 - 49	2 430
		50 - 54	2 440
		55 - 59	2 400
		60 - 64	2 760
		65 - 69	1 510

Noch: Personen nach Alter (5er-Jahresgruppen), Höchster beruflicher Abschluss (ausführlich) und weitere Merkmale für Niedersachsen (Bundesland)

			Niedersachsen (Bundesland)
		70 - 74	2 610
		75 - 79	3 020
		80 - 84	2 070
		85 - 89	310
		90 und älter	/
		Insgesamt	15 910
		Unter 5	/
		5 - 9	/
		10 - 14	/
		15 - 19	/
		20 - 24	440
		25 - 29	460
		30 - 34	680
		35 - 39	710
	Weiblich	40 - 44	850
		45 - 49	900
		50 - 54	930
		55 - 59	1 550
		60 - 64	1 450
		65 - 69	1 290
		70 - 74	1 670
		75 - 79	2 240
		80 - 84	1 660
		85 - 89	630
		90 und älter	360
		Insgesamt	5 630
		Unter 5	/
		5 - 9	/
		10 - 14	/
		15 - 19	/
		20 - 24	/
		25 - 29	/
		30 - 34	/
		35 - 39	/
	Insgesamt	40 - 44	390
		45 - 49	670
		50 - 54	570
		55 - 59	320
Fachschulabschluss		60 - 64	610
		65 - 69	510
		70 - 74	470
		75 - 79	320
		80 - 84	500
		85 - 89	/
		90 und älter	/
		Insgesamt	3 670
		Unter 5	/
		5 - 9	/
	Männlich	10 - 14	/
		15 - 19	/
		20 - 24	/
		25 - 29	/
		30 - 34	/

Noch: Personen nach Alter (5er-Jahresgruppen), Höchster beruflicher Abschluss (ausführlich) und weitere Merkmale für Niedersachsen (Bundesland)

			Niedersachsen (Bundesland)
		35 - 39	/
		40 - 44	/
		45 - 49	/
		50 - 54	/
		55 - 59	/
		60 - 64	/
		65 - 69	/
		70 - 74	/
		75 - 79	/
		80 - 84	/
		85 - 89	/
		90 und älter	/
		Insgesamt	1 970
		Unter 5	/
		5 - 9	/
		10 - 14	/
		15 - 19	/
		20 - 24	/
		25 - 29	/
		30 - 34	/
		35 - 39	/
	Weiblich	40 - 44	/
		45 - 49	/
		50 - 54	/
		55 - 59	/
		60 - 64	/
		65 - 69	/
		70 - 74	/
		75 - 79	/
		80 - 84	/
		85 - 89	/
		90 und älter	/
		Insgesamt	/
		Unter 5	/
		5 - 9	/
		10 - 14	/
		15 - 19	/
		20 - 24	/
		25 - 29	/
		30 - 34	/
		35 - 39	/
Abschluss einer Fachakademie oder Berufsakademie	Insgesamt	40 - 44	/
		45 - 49	/
		50 - 54	/
		55 - 59	/
		60 - 64	/
		65 - 69	/
		70 - 74	/
		75 - 79	/
		80 - 84	/
		85 - 89	/
		90 und älter	/
	Männlich	Insgesamt	/

Noch: Personen nach Alter (5er-Jahresgruppen), Höchster beruflicher Abschluss (ausführlich) und weitere Merkmale für Niedersachsen (Bundesland)

			Niedersachsen (Bundesland)
		Unter 5	/
		5 - 9	/
		10 - 14	/
		15 - 19	/
		20 - 24	/
		25 - 29	/
		30 - 34	/
		35 - 39	/
		40 - 44	/
		45 - 49	/
		50 - 54	/
		55 - 59	/
		60 - 64	/
		65 - 69	/
		70 - 74	/
		75 - 79	/
		80 - 84	/
		85 - 89	/
		90 und älter	/
		Insgesamt	/
	Weiblich	Unter 5	/
		5 - 9	/
		10 - 14	/
		15 - 19	/
		20 - 24	/
		25 - 29	/
		30 - 34	/
		35 - 39	/
		40 - 44	/
		45 - 49	/
		50 - 54	/
		55 - 59	/
		60 - 64	/
		65 - 69	/
		70 - 74	/
		75 - 79	/
		80 - 84	/
		85 - 89	/
		90 und älter	/
		Insgesamt	/
Fachhochschulabschluss	Insgesamt	Unter 5	/
		5 - 9	/
		10 - 14	/
		15 - 19	/
		20 - 24	/
		25 - 29	/
		30 - 34	/
		35 - 39	/
		40 - 44	/
		45 - 49	/
		50 - 54	/
		55 - 59	/
		60 - 64	/

Noch: Personen nach Alter (5er-Jahresgruppen), Höchster beruflicher Abschluss (ausführlich) und weitere Merkmale für Niedersachsen (Bundesland)

			Niedersachsen (Bundesland)
		65 - 69	/
		70 - 74	/
		75 - 79	/
		80 - 84	/
		85 - 89	/
		90 und älter	/
		Insgesamt	/
	Männlich	Unter 5	/
		5 - 9	/
		10 - 14	/
		15 - 19	/
		20 - 24	/
		25 - 29	/
		30 - 34	/
		35 - 39	/
		40 - 44	/
		45 - 49	/
		50 - 54	/
		55 - 59	/
		60 - 64	/
		65 - 69	/
		70 - 74	/
		75 - 79	/
		80 - 84	/
		85 - 89	/
		90 und älter	/
		Insgesamt	/
	Weiblich	Unter 5	/
		5 - 9	/
		10 - 14	/
		15 - 19	/
		20 - 24	/
		25 - 29	/
		30 - 34	/
		35 - 39	/
		40 - 44	/
		45 - 49	/
		50 - 54	/
		55 - 59	/
		60 - 64	/
		65 - 69	/
		70 - 74	/
		75 - 79	/
		80 - 84	/
		85 - 89	/
		90 und älter	/
		Insgesamt	/
Hochschulabschluss	Insgesamt	Unter 5	/
		5 - 9	/
		10 - 14	/
		15 - 19	/
		20 - 24	/
		25 - 29	/

Noch: Personen nach Alter (5er-Jahresgruppen), Höchster beruflicher Abschluss (ausführlich) und weitere Merkmale für Niedersachsen (Bundesland)

		Niedersachsen (Bundesland)
	30 - 34	/
	35 - 39	/
	40 - 44	/
	45 - 49	/
	50 - 54	/
	55 - 59	/
	60 - 64	/
	65 - 69	/
	70 - 74	/
	75 - 79	/
	80 - 84	/
	85 - 89	/
	90 und älter	/
Männlich	Insgesamt	/
	Unter 5	/
	5 - 9	/
	10 - 14	/
	15 - 19	/
	20 - 24	/
	25 - 29	/
	30 - 34	/
	35 - 39	/
	40 - 44	/
	45 - 49	/
	50 - 54	/
	55 - 59	/
	60 - 64	/
	65 - 69	/
	70 - 74	/
	75 - 79	/
	80 - 84	/
	85 - 89	/
	90 und älter	/
Weiblich	Insgesamt	/
	Unter 5	/
	5 - 9	/
	10 - 14	/
	15 - 19	/
	20 - 24	/
	25 - 29	/
	30 - 34	/
	35 - 39	/
	40 - 44	/
	45 - 49	/
	50 - 54	/
	55 - 59	/
	60 - 64	/
	65 - 69	/
	70 - 74	/
	75 - 79	/
	80 - 84	/
	85 - 89	/
	90 und älter	/

Noch: Personen nach Alter (5er-Jahresgruppen), Höchster beruflicher Abschluss (ausführlich) und weitere Merkmale für Niedersachsen (Bundesland)

			Niedersachsen (Bundesland)
		Insgesamt	/
		Unter 5	/
		5 - 9	/
		10 - 14	/
		15 - 19	/
		20 - 24	/
		25 - 29	/
		30 - 34	/
		35 - 39	/
		40 - 44	/
		45 - 49	/
		50 - 54	/
		55 - 59	/
		60 - 64	/
		65 - 69	/
		70 - 74	/
		75 - 79	/
		80 - 84	/
		85 - 89	/
		90 und älter	/
Promotion	Insgesamt	Insgesamt	/
		Unter 5	/
		5 - 9	/
		10 - 14	/
		15 - 19	/
		20 - 24	/
		25 - 29	/
		30 - 34	/
		35 - 39	/
		40 - 44	/
		45 - 49	/
		50 - 54	/
		55 - 59	/
		60 - 64	/
		65 - 69	/
		70 - 74	/
		75 - 79	/
		80 - 84	/
		85 - 89	/
		90 und älter	/
	Männlich	Insgesamt	/
		Unter 5	/
		5 - 9	/
		10 - 14	/
		15 - 19	/
		20 - 24	/
		25 - 29	/
		30 - 34	/
		35 - 39	/
		40 - 44	/
		45 - 49	/
		50 - 54	/
		55 - 59	/
		60 - 64	/
		65 - 69	/
		70 - 74	/
		75 - 79	/
		80 - 84	/
		85 - 89	/
		90 und älter	/
	Weiblich	Insgesamt	/
		Unter 5	/
		5 - 9	/
		10 - 14	/
		15 - 19	/
		20 - 24	/
		25 - 29	/
		30 - 34	/
		35 - 39	/
		40 - 44	/
		45 - 49	/
		50 - 54	/
		55 - 59	/

Noch: Personen nach Alter (5er-Jahresgruppen), Höchster beruflicher Abschluss (ausführlich) und weitere Merkmale für Niedersachsen (Bundesland)

				Niedersachsen (Bundesland)
			60 - 64	/
			65 - 69	/
			70 - 74	/
			75 - 79	/
			80 - 84	/
			85 - 89	/
			90 und älter	/
1.1 Ohne Schulabschluss	Insgesamt	Insgesamt	Insgesamt	294 690
			Unter 5	/
			5 - 9	/
			10 - 14	/
			15 - 19	9 970
			20 - 24	17 060
			25 - 29	16 660
			30 - 34	19 290
			35 - 39	20 980
			40 - 44	25 270
			45 - 49	28 750
			50 - 54	26 000
			55 - 59	20 610
			60 - 64	18 640
			65 - 69	16 080
			70 - 74	23 000
			75 - 79	25 120
			80 - 84	16 860
			85 - 89	7 050
			90 und älter	3 330
		Männlich	Insgesamt	138 470
			Unter 5	/
			5 - 9	/
			10 - 14	/
			15 - 19	6 020
			20 - 24	9 630
			25 - 29	8 700
			30 - 34	10 070
			35 - 39	9 670
			40 - 44	12 670
			45 - 49	15 280
			50 - 54	13 720
			55 - 59	10 070
			60 - 64	8 650
			65 - 69	7 540
			70 - 74	9 770
			75 - 79	9 220
			80 - 84	5 390
			85 - 89	1 570
			90 und älter	500
		Weiblich	Insgesamt	156 220
			Unter 5	/
			5 - 9	/
			10 - 14	/
			15 - 19	3 960
			20 - 24	7 430

Noch: Personen nach Alter (5er-Jahresgruppen), Höchster beruflicher Abschluss (ausführlich) und weitere Merkmale für Niedersachsen (Bundesland)

			Niedersachsen (Bundesland)
		25 - 29	7 960
		30 - 34	9 220
		35 - 39	11 310
		40 - 44	12 600
		45 - 49	13 460
		50 - 54	12 270
		55 - 59	10 550
		60 - 64	9 990
		65 - 69	8 540
		70 - 74	13 230
		75 - 79	15 900
		80 - 84	11 470
		85 - 89	5 480
		90 und älter	2 840
Ohne beruflichen Abschluss	Insgesamt	Insgesamt	247 850
		Unter 5	/
		5 - 9	/
		10 - 14	/
		15 - 19	9 780
		20 - 24	16 170
		25 - 29	14 970
		30 - 34	17 260
		35 - 39	18 450
		40 - 44	22 190
		45 - 49	24 780
		50 - 54	22 070
		55 - 59	16 340
		60 - 64	13 820
		65 - 69	12 780
		70 - 74	18 260
		75 - 79	19 550
		80 - 84	12 630
		85 - 89	6 060
		90 und älter	2 730
	Männlich	Insgesamt	109 200
		Unter 5	/
		5 - 9	/
		10 - 14	/
		15 - 19	5 880
		20 - 24	9 070
		25 - 29	7 540
		30 - 34	8 760
		35 - 39	7 920
		40 - 44	10 540
		45 - 49	12 410
		50 - 54	10 900
		55 - 59	7 460
		60 - 64	5 560
		65 - 69	5 720
		70 - 74	6 830
		75 - 79	5 990
		80 - 84	3 040
		85 - 89	1 220

Noch: Personen nach Alter (5er-Jahresgruppen), Höchster beruflicher Abschluss (ausführlich) und weitere Merkmale für Niedersachsen (Bundesland)

			Niedersachsen (Bundesland)
		90 und älter	360
	Weiblich	Insgesamt	138 650
		Unter 5	/
		5 - 9	/
		10 - 14	/
		15 - 19	3 900
		20 - 24	7 100
		25 - 29	7 430
		30 - 34	8 500
		35 - 39	10 520
		40 - 44	11 650
		45 - 49	12 370
		50 - 54	11 170
		55 - 59	8 890
		60 - 64	8 260
		65 - 69	7 050
		70 - 74	11 430
		75 - 79	13 560
		80 - 84	9 590
		85 - 89	4 850
		90 und älter	2 380
Lehre, Berufsausbildung im dualen System	Insgesamt	Insgesamt	41 550
		Unter 5	/
		5 - 9	/
		10 - 14	/
		15 - 19	/
		20 - 24	800
		25 - 29	1 440
		30 - 34	1 880
		35 - 39	2 240
		40 - 44	2 700
		45 - 49	3 300
		50 - 54	3 360
		55 - 59	3 950
		60 - 64	4 210
		65 - 69	2 800
		70 - 74	4 280
		75 - 79	5 260
		80 - 84	3 730
		85 - 89	950
		90 und älter	480
	Männlich	Insgesamt	25 820
		Unter 5	/
		5 - 9	/
		10 - 14	/
		15 - 19	/
		20 - 24	480
		25 - 29	970
		30 - 34	1 200
		35 - 39	1 540
		40 - 44	1 850
		45 - 49	2 410
		50 - 54	2 440

Noch: Personen nach Alter (5er-Jahresgruppen), Höchster beruflicher Abschluss (ausführlich) und weitere Merkmale für Niedersachsen (Bundesland)

			Niedersachsen (Bundesland)
		55 - 59	2 400
		60 - 64	2 760
		65 - 69	1 510
		70 - 74	2 610
		75 - 79	3 020
		80 - 84	2 070
		85 - 89	310
		90 und älter	/
	Weiblich	Insgesamt	15 740
		Unter 5	/
		5 - 9	/
		10 - 14	/
		15 - 19	/
		20 - 24	/
		25 - 29	460
		30 - 34	680
		35 - 39	710
		40 - 44	850
		45 - 49	890
		50 - 54	930
		55 - 59	1 550
		60 - 64	1 450
		65 - 69	1 290
		70 - 74	1 670
		75 - 79	2 240
		80 - 84	1 660
		85 - 89	630
		90 und älter	360
Fachschulabschluss	Insgesamt	Insgesamt	5 290
		Unter 5	/
		5 - 9	/
		10 - 14	/
		15 - 19	/
		20 - 24	/
		25 - 29	/
		30 - 34	/
		35 - 39	/
		40 - 44	390
		45 - 49	670
		50 - 54	570
		55 - 59	320
		60 - 64	610
		65 - 69	510
		70 - 74	470
		75 - 79	320
		80 - 84	500
		85 - 89	/
		90 und älter	/
	Männlich	Insgesamt	3 460
		Unter 5	/
		5 - 9	/
		10 - 14	/
		15 - 19	/

Noch: Personen nach Alter (5er-Jahresgruppen), Höchster beruflicher Abschluss (ausführlich) und weitere Merkmale für Niedersachsen (Bundesland)

			Niedersachsen (Bundesland)
		20 - 24	/
		25 - 29	/
		30 - 34	/
		35 - 39	/
		40 - 44	/
		45 - 49	/
		50 - 54	/
		55 - 59	/
		60 - 64	/
		65 - 69	/
		70 - 74	/
		75 - 79	/
		80 - 84	/
		85 - 89	/
		90 und älter	/
	Weiblich	Insgesamt	1 830
		Unter 5	/
		5 - 9	/
		10 - 14	/
		15 - 19	/
		20 - 24	/
		25 - 29	/
		30 - 34	/
		35 - 39	/
		40 - 44	/
		45 - 49	/
		50 - 54	/
		55 - 59	/
		60 - 64	/
		65 - 69	/
		70 - 74	/
		75 - 79	/
		80 - 84	/
		85 - 89	/
		90 und älter	/
Abschluss einer Fachakademie oder Berufsakademie	Insgesamt	Insgesamt	/
		Unter 5	/
		5 - 9	/
		10 - 14	/
		15 - 19	/
		20 - 24	/
		25 - 29	/
		30 - 34	/
		35 - 39	/
		40 - 44	/
		45 - 49	/
		50 - 54	/
		55 - 59	/
		60 - 64	/
		65 - 69	/
		70 - 74	/
		75 - 79	/
		80 - 84	/

Noch: Personen nach Alter (5er-Jahresgruppen), Höchster beruflicher Abschluss (ausführlich) und weitere Merkmale für Niedersachsen (Bundesland)

			Niedersachsen (Bundesland)
		85 - 89	/
		90 und älter	/
	Männlich	Insgesamt	/
		Unter 5	/
		5 - 9	/
		10 - 14	/
		15 - 19	/
		20 - 24	/
		25 - 29	/
		30 - 34	/
		35 - 39	/
		40 - 44	/
		45 - 49	/
		50 - 54	/
		55 - 59	/
		60 - 64	/
		65 - 69	/
		70 - 74	/
		75 - 79	/
		80 - 84	/
		85 - 89	/
		90 und älter	/
	Weiblich	Insgesamt	/
		Unter 5	/
		5 - 9	/
		10 - 14	/
		15 - 19	/
		20 - 24	/
		25 - 29	/
		30 - 34	/
		35 - 39	/
		40 - 44	/
		45 - 49	/
		50 - 54	/
		55 - 59	/
		60 - 64	/
		65 - 69	/
		70 - 74	/
		75 - 79	/
		80 - 84	/
		85 - 89	/
		90 und älter	/
Fachhochschulabschluss	Insgesamt	Insgesamt	/
		Unter 5	/
		5 - 9	/
		10 - 14	/
		15 - 19	/
		20 - 24	/
		25 - 29	/
		30 - 34	/
		35 - 39	/
		40 - 44	/
		45 - 49	/

Noch: Personen nach Alter (5er-Jahresgruppen), Höchster beruflicher Abschluss (ausführlich) und weitere Merkmale für Niedersachsen (Bundesland)

			Niedersachsen (Bundesland)
		50 - 54	/
		55 - 59	/
		60 - 64	/
		65 - 69	/
		70 - 74	/
		75 - 79	/
		80 - 84	/
		85 - 89	/
		90 und älter	/
	Männlich	Insgesamt	/
		Unter 5	/
		5 - 9	/
		10 - 14	/
		15 - 19	/
		20 - 24	/
		25 - 29	/
		30 - 34	/
		35 - 39	/
		40 - 44	/
		45 - 49	/
		50 - 54	/
		55 - 59	/
		60 - 64	/
		65 - 69	/
		70 - 74	/
		75 - 79	/
		80 - 84	/
		85 - 89	/
		90 und älter	/
	Weiblich	Insgesamt	/
		Unter 5	/
		5 - 9	/
		10 - 14	/
		15 - 19	/
		20 - 24	/
		25 - 29	/
		30 - 34	/
		35 - 39	/
		40 - 44	/
		45 - 49	/
		50 - 54	/
		55 - 59	/
		60 - 64	/
		65 - 69	/
		70 - 74	/
		75 - 79	/
		80 - 84	/
		85 - 89	/
		90 und älter	/
Hochschulabschluss	Insgesamt	Insgesamt	/
		Unter 5	/
		5 - 9	/
		10 - 14	/

Noch: Personen nach Alter (5er-Jahresgruppen), Höchster beruflicher Abschluss (ausführlich) und weitere Merkmale für Niedersachsen (Bundesland)

		Niedersachsen (Bundesland)
	15 - 19	/
	20 - 24	/
	25 - 29	/
	30 - 34	/
	35 - 39	/
	40 - 44	/
	45 - 49	/
	50 - 54	/
	55 - 59	/
	60 - 64	/
	65 - 69	/
	70 - 74	/
	75 - 79	/
	80 - 84	/
	85 - 89	/
	90 und älter	/
Männlich	Insgesamt	/
	Unter 5	/
	5 - 9	/
	10 - 14	/
	15 - 19	/
	20 - 24	/
	25 - 29	/
	30 - 34	/
	35 - 39	/
	40 - 44	/
	45 - 49	/
	50 - 54	/
	55 - 59	/
	60 - 64	/
	65 - 69	/
	70 - 74	/
	75 - 79	/
	80 - 84	/
	85 - 89	/
	90 und älter	/
Weiblich	Insgesamt	/
	Unter 5	/
	5 - 9	/
	10 - 14	/
	15 - 19	/
	20 - 24	/
	25 - 29	/
	30 - 34	/
	35 - 39	/
	40 - 44	/
	45 - 49	/
	50 - 54	/
	55 - 59	/
	60 - 64	/
	65 - 69	/
	70 - 74	/
	75 - 79	/

Noch: Personen nach Alter (5er-Jahresgruppen), Höchster beruflicher Abschluss (ausführlich) und weitere Merkmale für Niedersachsen (Bundesland)

			Niedersachsen (Bundesland)
		80 - 84	/
		85 - 89	/
		90 und älter	/
Promotion	Insgesamt	Insgesamt	/
		Unter 5	/
		5 - 9	/
		10 - 14	/
		15 - 19	/
		20 - 24	/
		25 - 29	/
		30 - 34	/
		35 - 39	/
		40 - 44	/
		45 - 49	/
		50 - 54	/
		55 - 59	/
		60 - 64	/
		65 - 69	/
		70 - 74	/
		75 - 79	/
		80 - 84	/
		85 - 89	/
		90 und älter	/
	Männlich	Insgesamt	/
		Unter 5	/
		5 - 9	/
		10 - 14	/
		15 - 19	/
		20 - 24	/
		25 - 29	/
		30 - 34	/
		35 - 39	/
		40 - 44	/
		45 - 49	/
		50 - 54	/
		55 - 59	/
		60 - 64	/
		65 - 69	/
		70 - 74	/
		75 - 79	/
		80 - 84	/
		85 - 89	/
		90 und älter	/
	Weiblich	Insgesamt	/
		Unter 5	/
		5 - 9	/
		10 - 14	/
		15 - 19	/
		20 - 24	/
		25 - 29	/
		30 - 34	/
		35 - 39	/
		40 - 44	/

Noch: Personen nach Alter (5er-Jahresgruppen), Höchster beruflicher Abschluss (ausführlich) und weitere Merkmale für Niedersachsen (Bundesland)

				Niedersachsen (Bundesland)
			45 - 49	/
			50 - 54	/
			55 - 59	/
			60 - 64	/
			65 - 69	/
			70 - 74	/
			75 - 79	/
			80 - 84	/
			85 - 89	/
			90 und älter	/
1.2 Noch in schulischer Ausbildung	Insgesamt	Insgesamt	Insgesamt	192 820
			Unter 5	/
			5 - 9	/
			10 - 14	/
			15 - 19	189 080
			20 - 24	2 660
			25 - 29	500
			30 - 34	/
			35 - 39	/
			40 - 44	/
			45 - 49	/
			50 - 54	/
			55 - 59	/
			60 - 64	/
			65 - 69	/
			70 - 74	/
			75 - 79	/
			80 - 84	/
			85 - 89	/
			90 und älter	/
		Männlich	Insgesamt	102 490
			Unter 5	/
			5 - 9	/
			10 - 14	/
			15 - 19	100 250
			20 - 24	1 610
			25 - 29	/
			30 - 34	/
			35 - 39	/
			40 - 44	/
			45 - 49	/
			50 - 54	/
			55 - 59	/
			60 - 64	/
			65 - 69	/
			70 - 74	/
			75 - 79	/
			80 - 84	/
			85 - 89	/
			90 und älter	/
		Weiblich	Insgesamt	90 330
			Unter 5	/
			5 - 9	/

Noch: Personen nach Alter (5er-Jahresgruppen), Höchster beruflicher Abschluss (ausführlich) und weitere Merkmale für Niedersachsen (Bundesland)

			Niedersachsen (Bundesland)
		10 - 14	/
		15 - 19	88 840
		20 - 24	1 060
		25 - 29	/
		30 - 34	/
		35 - 39	/
		40 - 44	/
		45 - 49	/
		50 - 54	/
		55 - 59	/
		60 - 64	/
		65 - 69	/
		70 - 74	/
		75 - 79	/
		80 - 84	/
		85 - 89	/
		90 und älter	/
		Insgesamt	192 010
		Unter 5	/
		5 - 9	/
		10 - 14	/
		15 - 19	188 780
		20 - 24	2 340
		25 - 29	400
		30 - 34	/
		35 - 39	/
		40 - 44	/
	Insgesamt	45 - 49	/
		50 - 54	/
		55 - 59	/
		60 - 64	/
		65 - 69	/
		70 - 74	/
		75 - 79	/
Ohne beruflichen Abschluss		80 - 84	/
		85 - 89	/
		90 und älter	/
		Insgesamt	101 980
		Unter 5	/
		5 - 9	/
		10 - 14	/
		15 - 19	100 110
		20 - 24	1 410
		25 - 29	/
	Männlich	30 - 34	/
		35 - 39	/
		40 - 44	/
		45 - 49	/
		50 - 54	/
		55 - 59	/
		60 - 64	/
		65 - 69	/
		70 - 74	/

Noch: Personen nach Alter (5er-Jahresgruppen), Höchster beruflicher Abschluss (ausführlich) und weitere Merkmale für Niedersachsen (Bundesland)

			Niedersachsen (Bundesland)
		75 - 79	/
		80 - 84	/
		85 - 89	/
		90 und älter	/
		Insgesamt	90 030
		Unter 5	/
		5 - 9	/
		10 - 14	/
		15 - 19	88 670
		20 - 24	930
		25 - 29	/
		30 - 34	/
		35 - 39	/
		40 - 44	/
	Weiblich	45 - 49	/
		50 - 54	/
		55 - 59	/
		60 - 64	/
		65 - 69	/
		70 - 74	/
		75 - 79	/
		80 - 84	/
		85 - 89	/
		90 und älter	/
		Insgesamt	470
		Unter 5	/
		5 - 9	/
		10 - 14	/
		15 - 19	/
		20 - 24	/
		25 - 29	/
		30 - 34	/
		35 - 39	/
		40 - 44	/
	Insgesamt	45 - 49	/
		50 - 54	/
		55 - 59	/
		60 - 64	/
Lehre, Berufsausbildung im dualen System		65 - 69	/
		70 - 74	/
		75 - 79	/
		80 - 84	/
		85 - 89	/
		90 und älter	/
		Insgesamt	/
		Unter 5	/
		5 - 9	/
		10 - 14	/
	Männlich	15 - 19	/
		20 - 24	/
		25 - 29	/
		30 - 34	/
		35 - 39	/

Noch: Personen nach Alter (5er-Jahresgruppen), Höchster beruflicher Abschluss (ausführlich) und weitere Merkmale für Niedersachsen (Bundesland)

			Niedersachsen (Bundesland)
		40 - 44	/
		45 - 49	/
		50 - 54	/
		55 - 59	/
		60 - 64	/
		65 - 69	/
		70 - 74	/
		75 - 79	/
		80 - 84	/
		85 - 89	/
		90 und älter	/
		Insgesamt	/
		Unter 5	/
		5 - 9	/
		10 - 14	/
		15 - 19	/
		20 - 24	/
		25 - 29	/
		30 - 34	/
		35 - 39	/
	Weiblich	40 - 44	/
		45 - 49	/
		50 - 54	/
		55 - 59	/
		60 - 64	/
		65 - 69	/
		70 - 74	/
		75 - 79	/
		80 - 84	/
		85 - 89	/
		90 und älter	/
		Insgesamt	/
		Unter 5	/
		5 - 9	/
		10 - 14	/
		15 - 19	/
		20 - 24	/
		25 - 29	/
		30 - 34	/
		35 - 39	/
	Insgesamt	40 - 44	/
Fachschulabschluss		45 - 49	/
		50 - 54	/
		55 - 59	/
		60 - 64	/
		65 - 69	/
		70 - 74	/
		75 - 79	/
		80 - 84	/
		85 - 89	/
		90 und älter	/
		Insgesamt	/
	Männlich	Unter 5	/

Noch: Personen nach Alter (5er-Jahresgruppen), Höchster beruflicher Abschluss (ausführlich) und weitere Merkmale für Niedersachsen (Bundesland)

			Niedersachsen (Bundesland)
		5 - 9	/
		10 - 14	/
		15 - 19	/
		20 - 24	/
		25 - 29	/
		30 - 34	/
		35 - 39	/
		40 - 44	/
		45 - 49	/
		50 - 54	/
		55 - 59	/
		60 - 64	/
		65 - 69	/
		70 - 74	/
		75 - 79	/
		80 - 84	/
		85 - 89	/
		90 und älter	/
		Insgesamt	/
		Unter 5	/
		5 - 9	/
		10 - 14	/
		15 - 19	/
		20 - 24	/
		25 - 29	/
		30 - 34	/
		35 - 39	/
	Weiblich	40 - 44	/
		45 - 49	/
		50 - 54	/
		55 - 59	/
		60 - 64	/
		65 - 69	/
		70 - 74	/
		75 - 79	/
		80 - 84	/
		85 - 89	/
		90 und älter	/
		Insgesamt	/
		Unter 5	/
		5 - 9	/
		10 - 14	/
		15 - 19	/
		20 - 24	/
		25 - 29	/
Abschluss einer Fachakademie oder Berufsakademie	Insgesamt	30 - 34	/
		35 - 39	/
		40 - 44	/
		45 - 49	/
		50 - 54	/
		55 - 59	/
		60 - 64	/
		65 - 69	/

Noch: Personen nach Alter (5er-Jahresgruppen), Höchster beruflicher Abschluss (ausführlich) und weitere Merkmale für Niedersachsen (Bundesland)

			Niedersachsen (Bundesland)
		70 - 74	/
		75 - 79	/
		80 - 84	/
		85 - 89	/
		90 und älter	/
		Insgesamt	/
		Unter 5	/
		5 - 9	/
		10 - 14	/
		15 - 19	/
		20 - 24	/
		25 - 29	/
		30 - 34	/
		35 - 39	/
	Männlich	40 - 44	/
		45 - 49	/
		50 - 54	/
		55 - 59	/
		60 - 64	/
		65 - 69	/
		70 - 74	/
		75 - 79	/
		80 - 84	/
		85 - 89	/
		90 und älter	/
		Insgesamt	/
		Unter 5	/
		5 - 9	/
		10 - 14	/
		15 - 19	/
		20 - 24	/
		25 - 29	/
		30 - 34	/
		35 - 39	/
	Weiblich	40 - 44	/
		45 - 49	/
		50 - 54	/
		55 - 59	/
		60 - 64	/
		65 - 69	/
		70 - 74	/
		75 - 79	/
		80 - 84	/
		85 - 89	/
		90 und älter	/
		Insgesamt	/
		Unter 5	/
		5 - 9	/
Fachhochschulabschluss	Insgesamt	10 - 14	/
		15 - 19	/
		20 - 24	/
		25 - 29	/
		30 - 34	/

Noch: Personen nach Alter (5er-Jahresgruppen), Höchster beruflicher Abschluss (ausführlich) und weitere Merkmale für Niedersachsen (Bundesland)

			Niedersachsen (Bundesland)
		35 - 39	/
		40 - 44	/
		45 - 49	/
		50 - 54	/
		55 - 59	/
		60 - 64	/
		65 - 69	/
		70 - 74	/
		75 - 79	/
		80 - 84	/
		85 - 89	/
		90 und älter	/
		Insgesamt	/
		Unter 5	/
		5 - 9	/
		10 - 14	/
		15 - 19	/
		20 - 24	/
		25 - 29	/
		30 - 34	/
		35 - 39	/
	Männlich	40 - 44	/
		45 - 49	/
		50 - 54	/
		55 - 59	/
		60 - 64	/
		65 - 69	/
		70 - 74	/
		75 - 79	/
		80 - 84	/
		85 - 89	/
		90 und älter	/
		Insgesamt	/
		Unter 5	/
		5 - 9	/
		10 - 14	/
		15 - 19	/
		20 - 24	/
		25 - 29	/
		30 - 34	/
		35 - 39	/
	Weiblich	40 - 44	/
		45 - 49	/
		50 - 54	/
		55 - 59	/
		60 - 64	/
		65 - 69	/
		70 - 74	/
		75 - 79	/
		80 - 84	/
		85 - 89	/
		90 und älter	/
Hochschulabschluss	Insgesamt	Insgesamt	/

Noch: Personen nach Alter (5er-Jahresgruppen), Höchster beruflicher Abschluss (ausführlich) und weitere Merkmale für Niedersachsen (Bundesland)

			Niedersachsen (Bundesland)
		Unter 5	/
		5 - 9	/
		10 - 14	/
		15 - 19	/
		20 - 24	/
		25 - 29	/
		30 - 34	/
		35 - 39	/
		40 - 44	/
		45 - 49	/
		50 - 54	/
		55 - 59	/
		60 - 64	/
		65 - 69	/
		70 - 74	/
		75 - 79	/
		80 - 84	/
		85 - 89	/
		90 und älter	/
		Insgesamt	/
	Männlich	Unter 5	/
		5 - 9	/
		10 - 14	/
		15 - 19	/
		20 - 24	/
		25 - 29	/
		30 - 34	/
		35 - 39	/
		40 - 44	/
		45 - 49	/
		50 - 54	/
		55 - 59	/
		60 - 64	/
		65 - 69	/
		70 - 74	/
		75 - 79	/
		80 - 84	/
		85 - 89	/
		90 und älter	/
		Insgesamt	/
	Weiblich	Unter 5	/
		5 - 9	/
		10 - 14	/
		15 - 19	/
		20 - 24	/
		25 - 29	/
		30 - 34	/
		35 - 39	/
		40 - 44	/
		45 - 49	/
		50 - 54	/
		55 - 59	/
		60 - 64	/

Noch: Personen nach Alter (5er-Jahresgruppen), Höchster beruflicher Abschluss (ausführlich) und weitere Merkmale für Niedersachsen (Bundesland)

			Niedersachsen (Bundesland)
		65 - 69	/
		70 - 74	/
		75 - 79	/
		80 - 84	/
		85 - 89	/
		90 und älter	/
		Insgesamt	/
Promotion	Insgesamt	Unter 5	/
		5 - 9	/
		10 - 14	/
		15 - 19	/
		20 - 24	/
		25 - 29	/
		30 - 34	/
		35 - 39	/
		40 - 44	/
		45 - 49	/
		50 - 54	/
		55 - 59	/
		60 - 64	/
		65 - 69	/
		70 - 74	/
		75 - 79	/
		80 - 84	/
		85 - 89	/
		90 und älter	/
		Insgesamt	/
	Männlich	Unter 5	/
		5 - 9	/
		10 - 14	/
		15 - 19	/
		20 - 24	/
		25 - 29	/
		30 - 34	/
		35 - 39	/
		40 - 44	/
		45 - 49	/
		50 - 54	/
		55 - 59	/
		60 - 64	/
		65 - 69	/
		70 - 74	/
		75 - 79	/
		80 - 84	/
		85 - 89	/
		90 und älter	/
		Insgesamt	/
	Weiblich	Unter 5	/
		5 - 9	/
		10 - 14	/
		15 - 19	/
		20 - 24	/
		25 - 29	/

Noch: Personen nach Alter (5er-Jahresgruppen), Höchster beruflicher Abschluss (ausführlich) und weitere Merkmale für Niedersachsen (Bundesland)

				Niedersachsen (Bundesland)
			30 - 34	/
			35 - 39	/
			40 - 44	/
			45 - 49	/
			50 - 54	/
			55 - 59	/
			60 - 64	/
			65 - 69	/
			70 - 74	/
			75 - 79	/
			80 - 84	/
			85 - 89	/
			90 und älter	/
2 Haupt-/ Volksschulabschluss	Insgesamt	Insgesamt	Insgesamt	2 438 040
			Unter 5	/
			5 - 9	/
			10 - 14	/
			15 - 19	30 150
			20 - 24	64 610
			25 - 29	62 400
			30 - 34	69 870
			35 - 39	96 630
			40 - 44	157 170
			45 - 49	195 210
			50 - 54	218 780
			55 - 59	235 170
			60 - 64	250 250
			65 - 69	240 010
			70 - 74	318 760
			75 - 79	213 290
			80 - 84	157 320
			85 - 89	92 510
			90 und älter	35 910
		Männlich	Insgesamt	1 190 810
			Unter 5	/
			5 - 9	/
			10 - 14	/
			15 - 19	19 030
			20 - 24	41 090
			25 - 29	38 130
			30 - 34	41 900
			35 - 39	57 190
			40 - 44	92 840
			45 - 49	116 710
			50 - 54	118 270
			55 - 59	116 220
			60 - 64	117 970
			65 - 69	110 540
			70 - 74	143 440
			75 - 79	90 170
			80 - 84	55 840
			85 - 89	25 080
			90 und älter	6 390

Noch: Personen nach Alter (5er-Jahresgruppen), Höchster beruflicher Abschluss (ausführlich) und weitere Merkmale für Niedersachsen (Bundesland)

				Niedersachsen (Bundesland)
		Weiblich	Insgesamt	1 247 230
			Unter 5	/
			5 - 9	/
			10 - 14	/
			15 - 19	11 130
			20 - 24	23 520
			25 - 29	24 280
			30 - 34	27 960
			35 - 39	39 440
			40 - 44	64 330
			45 - 49	78 500
			50 - 54	100 510
			55 - 59	118 950
			60 - 64	132 280
			65 - 69	129 460
			70 - 74	175 320
			75 - 79	123 120
			80 - 84	101 480
			85 - 89	67 430
			90 und älter	29 530
	Ohne beruflichen Abschluss	Insgesamt	Insgesamt	778 850
			Unter 5	/
			5 - 9	/
			10 - 14	/
			15 - 19	27 320
			20 - 24	40 190
			25 - 29	27 960
			30 - 34	26 370
			35 - 39	26 550
			40 - 44	39 800
			45 - 49	49 460
			50 - 54	52 630
			55 - 59	49 060
			60 - 64	51 580
			65 - 69	56 120
			70 - 74	103 050
			75 - 79	92 290
			80 - 84	73 590
			85 - 89	43 370
			90 und älter	19 520
		Männlich	Insgesamt	252 650
			Unter 5	/
			5 - 9	/
			10 - 14	/
			15 - 19	17 000
			20 - 24	23 480
			25 - 29	14 940
			30 - 34	12 690
			35 - 39	13 400
			40 - 44	17 770
			45 - 49	23 210
			50 - 54	20 680
			55 - 59	17 160

Noch: Personen nach Alter (5er-Jahresgruppen), Höchster beruflicher Abschluss (ausführlich) und weitere Merkmale für Niedersachsen (Bundesland)

			Niedersachsen (Bundesland)
		60 - 64	14 790
		65 - 69	14 310
		70 - 74	24 050
		75 - 79	18 680
		80 - 84	12 980
		85 - 89	5 890
		90 und älter	1 620
		Insgesamt	526 190
		Unter 5	/
		5 - 9	/
		10 - 14	/
		15 - 19	10 330
		20 - 24	16 710
		25 - 29	13 020
		30 - 34	13 680
		35 - 39	13 140
	Weiblich	40 - 44	22 030
		45 - 49	26 250
		50 - 54	31 960
		55 - 59	31 900
		60 - 64	36 780
		65 - 69	41 810
		70 - 74	79 000
		75 - 79	73 600
		80 - 84	60 610
		85 - 89	37 480
		90 und älter	17 900
		Insgesamt	1 496 130
		Unter 5	/
		5 - 9	/
		10 - 14	/
		15 - 19	2 600
		20 - 24	23 120
		25 - 29	32 550
		30 - 34	40 600
		35 - 39	65 050
	Insgesamt	40 - 44	107 380
		45 - 49	133 250
		50 - 54	150 080
Lehre, Berufsausbildung im dualen System		55 - 59	167 470
		60 - 64	177 940
		65 - 69	162 690
		70 - 74	190 300
		75 - 79	107 320
		80 - 84	75 890
		85 - 89	44 760
		90 und älter	15 140
		Insgesamt	831 590
		Unter 5	/
	Männlich	5 - 9	/
		10 - 14	/
		15 - 19	1 950
		20 - 24	17 050

Noch: Personen nach Alter (5er-Jahresgruppen), Höchster beruflicher Abschluss (ausführlich) und weitere Merkmale für Niedersachsen (Bundesland)

			Niedersachsen (Bundesland)
		25 - 29	22 240
		30 - 34	27 510
		35 - 39	40 590
		40 - 44	68 040
		45 - 49	84 410
		50 - 54	87 230
		55 - 59	87 470
		60 - 64	89 670
		65 - 69	82 200
		70 - 74	101 830
		75 - 79	62 120
		80 - 84	37 900
		85 - 89	17 140
		90 und älter	4 260
		Insgesamt	664 540
		Unter 5	/
		5 - 9	/
		10 - 14	/
		15 - 19	660
		20 - 24	6 060
		25 - 29	10 310
		30 - 34	13 090
		35 - 39	24 460
	Weiblich	40 - 44	39 340
		45 - 49	48 840
		50 - 54	62 850
		55 - 59	80 000
		60 - 64	88 270
		65 - 69	80 490
		70 - 74	88 470
		75 - 79	45 200
		80 - 84	37 980
		85 - 89	27 620
		90 und älter	10 880
		Insgesamt	163 060
		Unter 5	/
		5 - 9	/
		10 - 14	/
		15 - 19	/
		20 - 24	1 310
		25 - 29	1 900
		30 - 34	2 890
		35 - 39	5 030
Fachschulabschluss	Insgesamt	40 - 44	9 980
		45 - 49	12 500
		50 - 54	16 070
		55 - 59	18 640
		60 - 64	20 740
		65 - 69	21 190
		70 - 74	25 410
		75 - 79	13 680
		80 - 84	7 850
		85 - 89	4 390

Noch: Personen nach Alter (5er-Jahresgruppen), Höchster beruflicher Abschluss (ausführlich) und weitere Merkmale für Niedersachsen (Bundesland)

			Niedersachsen (Bundesland)
		90 und älter	1 250
	Männlich	Insgesamt	106 570
		Unter 5	/
		5 - 9	/
		10 - 14	/
		15 - 19	/
		20 - 24	560
		25 - 29	950
		30 - 34	1 710
		35 - 39	3 200
		40 - 44	7 030
		45 - 49	9 090
		50 - 54	10 360
		55 - 59	11 590
		60 - 64	13 510
		65 - 69	14 040
		70 - 74	17 560
		75 - 79	9 370
		80 - 84	4 960
		85 - 89	2 050
		90 und älter	500
	Weiblich	Insgesamt	56 490
		Unter 5	/
		5 - 9	/
		10 - 14	/
		15 - 19	/
		20 - 24	750
		25 - 29	950
		30 - 34	1 190
		35 - 39	1 830
		40 - 44	2 950
		45 - 49	3 400
		50 - 54	5 710
		55 - 59	7 050
		60 - 64	7 230
		65 - 69	7 160
		70 - 74	7 850
		75 - 79	4 320
		80 - 84	2 890
		85 - 89	2 330
		90 und älter	740
Abschluss einer Fachakademie oder Berufsakademie	Insgesamt	Insgesamt	/
		Unter 5	/
		5 - 9	/
		10 - 14	/
		15 - 19	/
		20 - 24	/
		25 - 29	/
		30 - 34	/
		35 - 39	/
		40 - 44	/
		45 - 49	/
		50 - 54	/

Noch: Personen nach Alter (5er-Jahresgruppen), Höchster beruflicher Abschluss (ausführlich) und weitere Merkmale für Niedersachsen (Bundesland)

			Niedersachsen (Bundesland)
		55 - 59	/
		60 - 64	/
		65 - 69	/
		70 - 74	/
		75 - 79	/
		80 - 84	/
		85 - 89	/
		90 und älter	/
	Männlich	Insgesamt	/
		Unter 5	/
		5 - 9	/
		10 - 14	/
		15 - 19	/
		20 - 24	/
		25 - 29	/
		30 - 34	/
		35 - 39	/
		40 - 44	/
		45 - 49	/
		50 - 54	/
		55 - 59	/
		60 - 64	/
		65 - 69	/
		70 - 74	/
		75 - 79	/
		80 - 84	/
		85 - 89	/
		90 und älter	/
	Weiblich	Insgesamt	/
		Unter 5	/
		5 - 9	/
		10 - 14	/
		15 - 19	/
		20 - 24	/
		25 - 29	/
		30 - 34	/
		35 - 39	/
		40 - 44	/
		45 - 49	/
		50 - 54	/
		55 - 59	/
		60 - 64	/
		65 - 69	/
		70 - 74	/
		75 - 79	/
		80 - 84	/
		85 - 89	/
		90 und älter	/
Fachhochschulabschluss	Insgesamt	Insgesamt	/
		Unter 5	/
		5 - 9	/
		10 - 14	/
		15 - 19	/

Noch: Personen nach Alter (5er-Jahresgruppen), Höchster beruflicher Abschluss (ausführlich) und weitere Merkmale für Niedersachsen (Bundesland)

		Niedersachsen (Bundesland)
	20 - 24	/
	25 - 29	/
	30 - 34	/
	35 - 39	/
	40 - 44	/
	45 - 49	/
	50 - 54	/
	55 - 59	/
	60 - 64	/
	65 - 69	/
	70 - 74	/
	75 - 79	/
	80 - 84	/
	85 - 89	/
	90 und älter	/
	Insgesamt	/
	Unter 5	/
	5 - 9	/
	10 - 14	/
	15 - 19	/
	20 - 24	/
	25 - 29	/
	30 - 34	/
	35 - 39	/
	40 - 44	/
Männlich	45 - 49	/
	50 - 54	/
	55 - 59	/
	60 - 64	/
	65 - 69	/
	70 - 74	/
	75 - 79	/
	80 - 84	/
	85 - 89	/
	90 und älter	/
	Insgesamt	/
	Unter 5	/
	5 - 9	/
	10 - 14	/
	15 - 19	/
	20 - 24	/
	25 - 29	/
	30 - 34	/
	35 - 39	/
Weiblich	40 - 44	/
	45 - 49	/
	50 - 54	/
	55 - 59	/
	60 - 64	/
	65 - 69	/
	70 - 74	/
	75 - 79	/
	80 - 84	/

Noch: Personen nach Alter (5er-Jahresgruppen), Höchster beruflicher Abschluss (ausführlich) und weitere Merkmale für Niedersachsen (Bundesland)

			Niedersachsen (Bundesland)
		85 - 89	/
		90 und älter	/
		Insgesamt	/
		Unter 5	/
		5 - 9	/
		10 - 14	/
		15 - 19	/
		20 - 24	/
		25 - 29	/
		30 - 34	/
		35 - 39	/
	Insgesamt	40 - 44	/
		45 - 49	/
		50 - 54	/
		55 - 59	/
		60 - 64	/
		65 - 69	/
		70 - 74	/
		75 - 79	/
		80 - 84	/
		85 - 89	/
		90 und älter	/
		Insgesamt	/
		Unter 5	/
		5 - 9	/
		10 - 14	/
		15 - 19	/
Hochschulabschluss		20 - 24	/
		25 - 29	/
		30 - 34	/
		35 - 39	/
		40 - 44	/
	Männlich	45 - 49	/
		50 - 54	/
		55 - 59	/
		60 - 64	/
		65 - 69	/
		70 - 74	/
		75 - 79	/
		80 - 84	/
		85 - 89	/
		90 und älter	/
		Insgesamt	/
		Unter 5	/
		5 - 9	/
		10 - 14	/
		15 - 19	/
	Weiblich	20 - 24	/
		25 - 29	/
		30 - 34	/
		35 - 39	/
		40 - 44	/
		45 - 49	/

Noch: Personen nach Alter (5er-Jahresgruppen), Höchster beruflicher Abschluss (ausführlich) und weitere Merkmale für Niedersachsen (Bundesland)

			Niedersachsen (Bundesland)
		50 - 54	/
		55 - 59	/
		60 - 64	/
		65 - 69	/
		70 - 74	/
		75 - 79	/
		80 - 84	/
		85 - 89	/
		90 und älter	/
Promotion	Insgesamt	Insgesamt	/
		Unter 5	/
		5 - 9	/
		10 - 14	/
		15 - 19	/
		20 - 24	/
		25 - 29	/
		30 - 34	/
		35 - 39	/
		40 - 44	/
		45 - 49	/
		50 - 54	/
		55 - 59	/
		60 - 64	/
		65 - 69	/
		70 - 74	/
		75 - 79	/
		80 - 84	/
		85 - 89	/
		90 und älter	/
	Männlich	Insgesamt	/
		Unter 5	/
		5 - 9	/
		10 - 14	/
		15 - 19	/
		20 - 24	/
		25 - 29	/
		30 - 34	/
		35 - 39	/
		40 - 44	/
		45 - 49	/
		50 - 54	/
		55 - 59	/
		60 - 64	/
		65 - 69	/
		70 - 74	/
		75 - 79	/
		80 - 84	/
		85 - 89	/
		90 und älter	/
	Weiblich	Insgesamt	/
		Unter 5	/
		5 - 9	/
		10 - 14	/

Noch: Personen nach Alter (5er-Jahresgruppen), Höchster beruflicher Abschluss (ausführlich) und weitere Merkmale für Niedersachsen (Bundesland)

				Niedersachsen (Bundesland)
			15 - 19	/
			20 - 24	/
			25 - 29	/
			30 - 34	/
			35 - 39	/
			40 - 44	/
			45 - 49	/
			50 - 54	/
			55 - 59	/
			60 - 64	/
			65 - 69	/
			70 - 74	/
			75 - 79	/
			80 - 84	/
			85 - 89	/
			90 und älter	/
3 Mittlerer Schulabschluss und gymnasiale Oberstufe	Insgesamt	Insgesamt	Insgesamt	2 057 220
			Unter 5	/
			5 - 9	/
			10 - 14	/
			15 - 19	192 750
			20 - 24	190 580
			25 - 29	167 230
			30 - 34	166 060
			35 - 39	178 590
			40 - 44	248 420
			45 - 49	252 520
			50 - 54	185 480
			55 - 59	128 450
			60 - 64	95 490
			65 - 69	80 290
			70 - 74	73 540
			75 - 79	44 600
			80 - 84	28 360
			85 - 89	16 750
			90 und älter	8 110
		Männlich	Insgesamt	905 180
			Unter 5	/
			5 - 9	/
			10 - 14	/
			15 - 19	91 930
			20 - 24	99 290
			25 - 29	83 280
			30 - 34	78 930
			35 - 39	77 350
			40 - 44	104 210
			45 - 49	103 540
			50 - 54	77 950
			55 - 59	53 420
			60 - 64	41 130
			65 - 69	32 470
			70 - 74	28 240
			75 - 79	17 390

Noch: Personen nach Alter (5er-Jahresgruppen), Höchster beruflicher Abschluss (ausführlich) und weitere Merkmale für Niedersachsen (Bundesland)

			Niedersachsen (Bundesland)
		80 - 84	10 500
		85 - 89	4 160
		90 und älter	1 390
		Insgesamt	1 152 040
		Unter 5	/
		5 - 9	/
		10 - 14	/
		15 - 19	100 820
		20 - 24	91 290
		25 - 29	83 950
		30 - 34	87 130
		35 - 39	101 240
	Weiblich	40 - 44	144 210
		45 - 49	148 980
		50 - 54	107 530
		55 - 59	75 020
		60 - 64	54 360
		65 - 69	47 810
		70 - 74	45 300
		75 - 79	27 220
		80 - 84	17 860
		85 - 89	12 600
		90 und älter	6 730
		Insgesamt	452 650
		Unter 5	/
		5 - 9	/
		10 - 14	/
		15 - 19	182 160
		20 - 24	71 030
		25 - 29	22 310
		30 - 34	19 820
		35 - 39	19 710
	Insgesamt	40 - 44	24 080
		45 - 49	27 150
		50 - 54	22 700
		55 - 59	15 140
		60 - 64	10 520
Ohne beruflichen Abschluss		65 - 69	8 470
		70 - 74	8 860
		75 - 79	7 260
		80 - 84	6 800
		85 - 89	4 460
		90 und älter	2 160
		Insgesamt	191 470
		Unter 5	/
		5 - 9	/
		10 - 14	/
	Männlich	15 - 19	86 570
		20 - 24	35 760
		25 - 29	10 600
		30 - 34	8 340
		35 - 39	7 660
		40 - 44	8 570

Noch: Personen nach Alter (5er-Jahresgruppen), Höchster beruflicher Abschluss (ausführlich) und weitere Merkmale für Niedersachsen (Bundesland)

			Niedersachsen (Bundesland)
		45 - 49	10 330
		50 - 54	8 760
		55 - 59	4 750
		60 - 64	3 080
		65 - 69	1 870
		70 - 74	2 000
		75 - 79	1 200
		80 - 84	1 210
		85 - 89	650
		90 und älter	/
		Insgesamt	261 170
		Unter 5	/
		5 - 9	/
		10 - 14	/
		15 - 19	95 590
		20 - 24	35 270
		25 - 29	11 710
		30 - 34	11 480
		35 - 39	12 050
	Weiblich	40 - 44	15 510
		45 - 49	16 830
		50 - 54	13 940
		55 - 59	10 390
		60 - 64	7 440
		65 - 69	6 600
		70 - 74	6 860
		75 - 79	6 060
		80 - 84	5 590
		85 - 89	3 810
		90 und älter	2 030
		Insgesamt	1 283 550
		Unter 5	/
		5 - 9	/
		10 - 14	/
		15 - 19	8 700
		20 - 24	104 990
		25 - 29	124 790
		30 - 34	122 990
		35 - 39	130 130
	Insgesamt	40 - 44	181 770
Lehre, Berufsausbildung im dualen System		45 - 49	175 310
		50 - 54	121 740
		55 - 59	86 530
		60 - 64	63 050
		65 - 69	54 640
		70 - 74	48 950
		75 - 79	28 780
		80 - 84	16 410
		85 - 89	9 790
		90 und älter	4 970
		Insgesamt	574 180
	Männlich	Unter 5	/
		5 - 9	/

Noch: Personen nach Alter (5er-Jahresgruppen), Höchster beruflicher Abschluss (ausführlich) und weitere Merkmale für Niedersachsen (Bundesland)

			Niedersachsen (Bundesland)
		10 - 14	/
		15 - 19	4 920
		20 - 24	58 670
		25 - 29	65 500
		30 - 34	60 860
		35 - 39	58 020
		40 - 44	75 590
		45 - 49	70 570
		50 - 54	51 490
		55 - 59	37 370
		60 - 64	27 250
		65 - 69	21 940
		70 - 74	18 960
		75 - 79	12 290
		80 - 84	6 840
		85 - 89	2 810
		90 und älter	1 110
	Weiblich	Insgesamt	709 370
		Unter 5	/
		5 - 9	/
		10 - 14	/
		15 - 19	3 780
		20 - 24	46 330
		25 - 29	59 290
		30 - 34	62 130
		35 - 39	72 120
		40 - 44	106 180
		45 - 49	104 740
		50 - 54	70 260
		55 - 59	49 160
		60 - 64	35 800
		65 - 69	32 700
		70 - 74	29 980
		75 - 79	16 490
		80 - 84	9 570
		85 - 89	6 980
		90 und älter	3 860
Fachschulabschluss	Insgesamt	Insgesamt	321 030
		Unter 5	/
		5 - 9	/
		10 - 14	/
		15 - 19	1 890
		20 - 24	14 550
		25 - 29	20 130
		30 - 34	23 250
		35 - 39	28 750
		40 - 44	42 580
		45 - 49	50 060
		50 - 54	41 040
		55 - 59	26 770
		60 - 64	21 910
		65 - 69	17 170
		70 - 74	15 730

Noch: Personen nach Alter (5er-Jahresgruppen), Höchster beruflicher Abschluss (ausführlich) und weitere Merkmale für Niedersachsen (Bundesland)

			Niedersachsen (Bundesland)
		75 - 79	8 570
		80 - 84	5 150
		85 - 89	2 500
		90 und älter	980
	Männlich	Insgesamt	139 540
		Unter 5	/
		5 - 9	/
		10 - 14	/
		15 - 19	450
		20 - 24	4 860
		25 - 29	7 180
		30 - 34	9 730
		35 - 39	11 680
		40 - 44	20 060
		45 - 49	22 640
		50 - 54	17 710
		55 - 59	11 290
		60 - 64	10 790
		65 - 69	8 660
		70 - 74	7 280
		75 - 79	3 910
		80 - 84	2 460
		85 - 89	690
		90 und älter	/
	Weiblich	Insgesamt	181 490
		Unter 5	/
		5 - 9	/
		10 - 14	/
		15 - 19	1 450
		20 - 24	9 690
		25 - 29	12 950
		30 - 34	13 510
		35 - 39	17 070
		40 - 44	22 520
		45 - 49	27 420
		50 - 54	23 330
		55 - 59	15 480
		60 - 64	11 120
		65 - 69	8 510
		70 - 74	8 460
		75 - 79	4 660
		80 - 84	2 690
		85 - 89	1 810
		90 und älter	830
Abschluss einer Fachakademie oder Berufsakademie	Insgesamt	Insgesamt	/
		Unter 5	/
		5 - 9	/
		10 - 14	/
		15 - 19	/
		20 - 24	/
		25 - 29	/
		30 - 34	/
		35 - 39	/

Noch: Personen nach Alter (5er-Jahresgruppen), Höchster beruflicher Abschluss (ausführlich) und weitere Merkmale für Niedersachsen (Bundesland)

			Niedersachsen (Bundesland)
		40 - 44	/
		45 - 49	/
		50 - 54	/
		55 - 59	/
		60 - 64	/
		65 - 69	/
		70 - 74	/
		75 - 79	/
		80 - 84	/
		85 - 89	/
		90 und älter	/
	Männlich	Insgesamt	/
		Unter 5	/
		5 - 9	/
		10 - 14	/
		15 - 19	/
		20 - 24	/
		25 - 29	/
		30 - 34	/
		35 - 39	/
		40 - 44	/
		45 - 49	/
		50 - 54	/
		55 - 59	/
		60 - 64	/
		65 - 69	/
		70 - 74	/
		75 - 79	/
		80 - 84	/
		85 - 89	/
		90 und älter	/
	Weiblich	Insgesamt	/
		Unter 5	/
		5 - 9	/
		10 - 14	/
		15 - 19	/
		20 - 24	/
		25 - 29	/
		30 - 34	/
		35 - 39	/
		40 - 44	/
		45 - 49	/
		50 - 54	/
		55 - 59	/
		60 - 64	/
		65 - 69	/
		70 - 74	/
		75 - 79	/
		80 - 84	/
		85 - 89	/
		90 und älter	/
Fachhochschulabschluss	Insgesamt	Insgesamt	/
		Unter 5	/

Noch: Personen nach Alter (5er-Jahresgruppen), Höchster beruflicher Abschluss (ausführlich) und weitere Merkmale für Niedersachsen (Bundesland)

		Niedersachsen (Bundesland)
	5 - 9	/
	10 - 14	/
	15 - 19	/
	20 - 24	/
	25 - 29	/
	30 - 34	/
	35 - 39	/
	40 - 44	/
	45 - 49	/
	50 - 54	/
	55 - 59	/
	60 - 64	/
	65 - 69	/
	70 - 74	/
	75 - 79	/
	80 - 84	/
	85 - 89	/
	90 und älter	/
Männlich	Insgesamt	/
	Unter 5	/
	5 - 9	/
	10 - 14	/
	15 - 19	/
	20 - 24	/
	25 - 29	/
	30 - 34	/
	35 - 39	/
	40 - 44	/
	45 - 49	/
	50 - 54	/
	55 - 59	/
	60 - 64	/
	65 - 69	/
	70 - 74	/
	75 - 79	/
	80 - 84	/
	85 - 89	/
	90 und älter	/
Weiblich	Insgesamt	/
	Unter 5	/
	5 - 9	/
	10 - 14	/
	15 - 19	/
	20 - 24	/
	25 - 29	/
	30 - 34	/
	35 - 39	/
	40 - 44	/
	45 - 49	/
	50 - 54	/
	55 - 59	/
	60 - 64	/
	65 - 69	/

Noch: Personen nach Alter (5er-Jahresgruppen), Höchster beruflicher Abschluss (ausführlich) und weitere Merkmale für Niedersachsen (Bundesland)

			Niedersachsen (Bundesland)
		70 - 74	/
		75 - 79	/
		80 - 84	/
		85 - 89	/
		90 und älter	/
		Insgesamt	/
		Unter 5	/
		5 - 9	/
		10 - 14	/
		15 - 19	/
		20 - 24	/
		25 - 29	/
		30 - 34	/
		35 - 39	/
		40 - 44	/
	Insgesamt	45 - 49	/
		50 - 54	/
		55 - 59	/
		60 - 64	/
		65 - 69	/
		70 - 74	/
		75 - 79	/
		80 - 84	/
		85 - 89	/
		90 und älter	/
		Insgesamt	/
		Unter 5	/
		5 - 9	/
		10 - 14	/
Hochschulabschluss		15 - 19	/
		20 - 24	/
		25 - 29	/
		30 - 34	/
		35 - 39	/
		40 - 44	/
	Männlich	45 - 49	/
		50 - 54	/
		55 - 59	/
		60 - 64	/
		65 - 69	/
		70 - 74	/
		75 - 79	/
		80 - 84	/
		85 - 89	/
		90 und älter	/
		Insgesamt	/
		Unter 5	/
		5 - 9	/
		10 - 14	/
	Weiblich	15 - 19	/
		20 - 24	/
		25 - 29	/
		30 - 34	/

Noch: Personen nach Alter (5er-Jahresgruppen), Höchster beruflicher Abschluss (ausführlich) und weitere Merkmale für Niedersachsen (Bundesland)

			Niedersachsen (Bundesland)
		35 - 39	/
		40 - 44	/
		45 - 49	/
		50 - 54	/
		55 - 59	/
		60 - 64	/
		65 - 69	/
		70 - 74	/
		75 - 79	/
		80 - 84	/
		85 - 89	/
		90 und älter	/
		Insgesamt	/
		Unter 5	/
		5 - 9	/
		10 - 14	/
		15 - 19	/
		20 - 24	/
		25 - 29	/
		30 - 34	/
		35 - 39	/
		40 - 44	/
	Insgesamt	45 - 49	/
		50 - 54	/
		55 - 59	/
		60 - 64	/
		65 - 69	/
		70 - 74	/
		75 - 79	/
		80 - 84	/
		85 - 89	/
		90 und älter	/
Promotion		Insgesamt	/
		Unter 5	/
		5 - 9	/
		10 - 14	/
		15 - 19	/
		20 - 24	/
		25 - 29	/
		30 - 34	/
		35 - 39	/
		40 - 44	/
	Männlich	45 - 49	/
		50 - 54	/
		55 - 59	/
		60 - 64	/
		65 - 69	/
		70 - 74	/
		75 - 79	/
		80 - 84	/
		85 - 89	/
		90 und älter	/
	Weiblich	Insgesamt	/

Noch: Personen nach Alter (5er-Jahresgruppen), Höchster beruflicher Abschluss (ausführlich) und weitere Merkmale für Niedersachsen (Bundesland)

				Niedersachsen (Bundesland)
			Unter 5	/
			5 - 9	/
			10 - 14	/
			15 - 19	/
			20 - 24	/
			25 - 29	/
			30 - 34	/
			35 - 39	/
			40 - 44	/
			45 - 49	/
			50 - 54	/
			55 - 59	/
			60 - 64	/
			65 - 69	/
			70 - 74	/
			75 - 79	/
			80 - 84	/
			85 - 89	/
			90 und älter	/
			Insgesamt	1 913 540
3.1 Realschul- oder gleichwertiger Abschluss	Insgesamt	Insgesamt	Unter 5	/
			5 - 9	/
			10 - 14	/
			15 - 19	69 510
			20 - 24	172 720
			25 - 29	165 570
			30 - 34	165 400
			35 - 39	178 450
			40 - 44	248 290
			45 - 49	252 520
			50 - 54	185 480
			55 - 59	128 450
			60 - 64	95 490
			65 - 69	80 290
			70 - 74	73 540
			75 - 79	44 600
			80 - 84	28 360
			85 - 89	16 750
			90 und älter	8 110
			Insgesamt	838 550
		Männlich	Unter 5	/
			5 - 9	/
			10 - 14	/
			15 - 19	36 170
			20 - 24	89 950
			25 - 29	82 230
			30 - 34	78 570
			35 - 39	77 270
			40 - 44	104 170
			45 - 49	103 540
			50 - 54	77 950
			55 - 59	53 420
			60 - 64	41 130

Noch: Personen nach Alter (5er-Jahresgruppen), Höchster beruflicher Abschluss (ausführlich) und weitere Merkmale für Niedersachsen (Bundesland)

				Niedersachsen (Bundesland)
			65 - 69	32 470
			70 - 74	28 240
			75 - 79	17 390
			80 - 84	10 500
			85 - 89	4 160
			90 und älter	1 390
			Insgesamt	1 074 990
		Weiblich	Unter 5	/
			5 - 9	/
			10 - 14	/
			15 - 19	33 340
			20 - 24	82 770
			25 - 29	83 340
			30 - 34	86 840
			35 - 39	101 180
			40 - 44	144 120
			45 - 49	148 980
			50 - 54	107 530
			55 - 59	75 020
			60 - 64	54 360
			65 - 69	47 810
			70 - 74	45 300
			75 - 79	27 220
			80 - 84	17 860
			85 - 89	12 600
			90 und älter	6 730
			Insgesamt	316 280
	Ohne beruflichen Abschluss	Insgesamt	Unter 5	/
			5 - 9	/
			10 - 14	/
			15 - 19	60 430
			20 - 24	57 290
			25 - 29	21 630
			30 - 34	19 650
			35 - 39	19 680
			40 - 44	24 050
			45 - 49	27 150
			50 - 54	22 700
			55 - 59	15 140
			60 - 64	10 520
			65 - 69	8 470
			70 - 74	8 860
			75 - 79	7 260
			80 - 84	6 800
			85 - 89	4 460
			90 und älter	2 160
			Insgesamt	128 760
		Männlich	Unter 5	/
			5 - 9	/
			10 - 14	/
			15 - 19	31 360
			20 - 24	28 720
			25 - 29	10 260

Noch: Personen nach Alter (5er-Jahresgruppen), Höchster beruflicher Abschluss (ausführlich) und weitere Merkmale für Niedersachsen (Bundesland)

			Niedersachsen (Bundesland)
		30 - 34	8 230
		35 - 39	7 660
		40 - 44	8 560
		45 - 49	10 330
		50 - 54	8 760
		55 - 59	4 750
		60 - 64	3 080
		65 - 69	1 870
		70 - 74	2 000
		75 - 79	1 200
		80 - 84	1 210
		85 - 89	650
		90 und älter	/
	Weiblich	Insgesamt	187 510
		Unter 5	/
		5 - 9	/
		10 - 14	/
		15 - 19	29 070
		20 - 24	28 570
		25 - 29	11 370
		30 - 34	11 420
		35 - 39	12 030
		40 - 44	15 490
		45 - 49	16 830
		50 - 54	13 940
		55 - 59	10 390
		60 - 64	7 440
		65 - 69	6 600
		70 - 74	6 860
		75 - 79	6 060
		80 - 84	5 590
		85 - 89	3 810
		90 und älter	2 030
Lehre, Berufsausbildung im dualen System	Insgesamt	Insgesamt	1 277 970
		Unter 5	/
		5 - 9	/
		10 - 14	/
		15 - 19	7 930
		20 - 24	101 510
		25 - 29	123 950
		30 - 34	122 700
		35 - 39	130 040
		40 - 44	181 670
		45 - 49	175 300
		50 - 54	121 740
		55 - 59	86 530
		60 - 64	63 050
		65 - 69	54 640
		70 - 74	48 950
		75 - 79	28 780
		80 - 84	16 410
		85 - 89	9 790
		90 und älter	4 970

Noch: Personen nach Alter (5er-Jahresgruppen), Höchster beruflicher Abschluss (ausführlich) und weitere Merkmale für Niedersachsen (Bundesland)

			Niedersachsen (Bundesland)
	Männlich	Insgesamt	570 880
		Unter 5	/
		5 - 9	/
		10 - 14	/
		15 - 19	4 560
		20 - 24	56 670
		25 - 29	64 840
		30 - 34	60 660
		35 - 39	57 960
		40 - 44	75 560
		45 - 49	70 570
		50 - 54	51 490
		55 - 59	37 370
		60 - 64	27 250
		65 - 69	21 940
		70 - 74	18 960
		75 - 79	12 290
		80 - 84	6 840
		85 - 89	2 810
		90 und älter	1 110
	Weiblich	Insgesamt	707 090
		Unter 5	/
		5 - 9	/
		10 - 14	/
		15 - 19	3 360
		20 - 24	44 840
		25 - 29	59 110
		30 - 34	62 040
		35 - 39	72 090
		40 - 44	106 110
		45 - 49	104 740
		50 - 54	70 260
		55 - 59	49 160
		60 - 64	35 800
		65 - 69	32 700
		70 - 74	29 980
		75 - 79	16 490
		80 - 84	9 570
		85 - 89	6 980
		90 und älter	3 860
Fachschulabschluss	Insgesamt	Insgesamt	319 290
		Unter 5	/
		5 - 9	/
		10 - 14	/
		15 - 19	1 150
		20 - 24	13 930
		25 - 29	19 990
		30 - 34	23 050
		35 - 39	28 730
		40 - 44	42 560
		45 - 49	50 060
		50 - 54	41 040
		55 - 59	26 770

Noch: Personen nach Alter (5er-Jahresgruppen), Höchster beruflicher Abschluss (ausführlich) und weitere Merkmale für Niedersachsen (Bundesland)

			Niedersachsen (Bundesland)
		60 - 64	21 910
		65 - 69	17 170
		70 - 74	15 730
		75 - 79	8 570
		80 - 84	5 150
		85 - 89	2 500
		90 und älter	980
	Männlich	Insgesamt	138 910
		Unter 5	/
		5 - 9	/
		10 - 14	/
		15 - 19	/
		20 - 24	4 570
		25 - 29	7 140
		30 - 34	9 680
		35 - 39	11 660
		40 - 44	20 040
		45 - 49	22 640
		50 - 54	17 710
		55 - 59	11 290
		60 - 64	10 790
		65 - 69	8 660
		70 - 74	7 280
		75 - 79	3 910
		80 - 84	2 460
		85 - 89	690
		90 und älter	/
	Weiblich	Insgesamt	180 380
		Unter 5	/
		5 - 9	/
		10 - 14	/
		15 - 19	910
		20 - 24	9 360
		25 - 29	12 850
		30 - 34	13 370
		35 - 39	17 070
		40 - 44	22 520
		45 - 49	27 420
		50 - 54	23 330
		55 - 59	15 480
		60 - 64	11 120
		65 - 69	8 510
		70 - 74	8 460
		75 - 79	4 660
		80 - 84	2 690
		85 - 89	1 810
		90 und älter	830
Abschluss einer Fachakademie oder Berufsakademie	Insgesamt	Insgesamt	/
		Unter 5	/
		5 - 9	/
		10 - 14	/
		15 - 19	/
		20 - 24	/

Noch: Personen nach Alter (5er-Jahresgruppen), Höchster beruflicher Abschluss (ausführlich) und weitere Merkmale für Niedersachsen (Bundesland)

			Niedersachsen (Bundesland)
		25 - 29	/
		30 - 34	/
		35 - 39	/
		40 - 44	/
		45 - 49	/
		50 - 54	/
		55 - 59	/
		60 - 64	/
		65 - 69	/
		70 - 74	/
		75 - 79	/
		80 - 84	/
		85 - 89	/
		90 und älter	/
	Männlich	Insgesamt	/
		Unter 5	/
		5 - 9	/
		10 - 14	/
		15 - 19	/
		20 - 24	/
		25 - 29	/
		30 - 34	/
		35 - 39	/
		40 - 44	/
		45 - 49	/
		50 - 54	/
		55 - 59	/
		60 - 64	/
		65 - 69	/
		70 - 74	/
		75 - 79	/
		80 - 84	/
		85 - 89	/
		90 und älter	/
	Weiblich	Insgesamt	/
		Unter 5	/
		5 - 9	/
		10 - 14	/
		15 - 19	/
		20 - 24	/
		25 - 29	/
		30 - 34	/
		35 - 39	/
		40 - 44	/
		45 - 49	/
		50 - 54	/
		55 - 59	/
		60 - 64	/
		65 - 69	/
		70 - 74	/
		75 - 79	/
		80 - 84	/
		85 - 89	/

Noch: Personen nach Alter (5er-Jahresgruppen), Höchster beruflicher Abschluss (ausführlich) und weitere Merkmale für Niedersachsen (Bundesland)

			Niedersachsen (Bundesland)
		90 und älter	/
Fachhochschulabschluss	Insgesamt	Insgesamt	/
		Unter 5	/
		5 - 9	/
		10 - 14	/
		15 - 19	/
		20 - 24	/
		25 - 29	/
		30 - 34	/
		35 - 39	/
		40 - 44	/
		45 - 49	/
		50 - 54	/
		55 - 59	/
		60 - 64	/
		65 - 69	/
		70 - 74	/
		75 - 79	/
		80 - 84	/
		85 - 89	/
		90 und älter	/
	Männlich	Insgesamt	/
		Unter 5	/
		5 - 9	/
		10 - 14	/
		15 - 19	/
		20 - 24	/
		25 - 29	/
		30 - 34	/
		35 - 39	/
		40 - 44	/
		45 - 49	/
		50 - 54	/
		55 - 59	/
		60 - 64	/
		65 - 69	/
		70 - 74	/
		75 - 79	/
		80 - 84	/
		85 - 89	/
		90 und älter	/
	Weiblich	Insgesamt	/
		Unter 5	/
		5 - 9	/
		10 - 14	/
		15 - 19	/
		20 - 24	/
		25 - 29	/
		30 - 34	/
		35 - 39	/
		40 - 44	/
		45 - 49	/
		50 - 54	/

Noch: Personen nach Alter (5er-Jahresgruppen), Höchster beruflicher Abschluss (ausführlich) und weitere Merkmale für Niedersachsen (Bundesland)

			Niedersachsen (Bundesland)
		55 - 59	/
		60 - 64	/
		65 - 69	/
		70 - 74	/
		75 - 79	/
		80 - 84	/
		85 - 89	/
		90 und älter	/
Hochschulabschluss	Insgesamt	Insgesamt	/
		Unter 5	/
		5 - 9	/
		10 - 14	/
		15 - 19	/
		20 - 24	/
		25 - 29	/
		30 - 34	/
		35 - 39	/
		40 - 44	/
		45 - 49	/
		50 - 54	/
		55 - 59	/
		60 - 64	/
		65 - 69	/
		70 - 74	/
		75 - 79	/
		80 - 84	/
		85 - 89	/
		90 und älter	/
	Männlich	Insgesamt	/
		Unter 5	/
		5 - 9	/
		10 - 14	/
		15 - 19	/
		20 - 24	/
		25 - 29	/
		30 - 34	/
		35 - 39	/
		40 - 44	/
		45 - 49	/
		50 - 54	/
		55 - 59	/
		60 - 64	/
		65 - 69	/
		70 - 74	/
		75 - 79	/
		80 - 84	/
		85 - 89	/
		90 und älter	/
	Weiblich	Insgesamt	/
		Unter 5	/
		5 - 9	/
		10 - 14	/
		15 - 19	/

Noch: Personen nach Alter (5er-Jahresgruppen), Höchster beruflicher Abschluss (ausführlich) und weitere Merkmale für Niedersachsen (Bundesland)

			Niedersachsen (Bundesland)
		20 - 24	/
		25 - 29	/
		30 - 34	/
		35 - 39	/
		40 - 44	/
		45 - 49	/
		50 - 54	/
		55 - 59	/
		60 - 64	/
		65 - 69	/
		70 - 74	/
		75 - 79	/
		80 - 84	/
		85 - 89	/
		90 und älter	/
		Insgesamt	/
		Unter 5	/
		5 - 9	/
		10 - 14	/
		15 - 19	/
		20 - 24	/
		25 - 29	/
		30 - 34	/
		35 - 39	/
		40 - 44	/
	Insgesamt	45 - 49	/
		50 - 54	/
		55 - 59	/
		60 - 64	/
		65 - 69	/
		70 - 74	/
		75 - 79	/
		80 - 84	/
		85 - 89	/
Promotion		90 und älter	/
		Insgesamt	/
		Unter 5	/
		5 - 9	/
		10 - 14	/
		15 - 19	/
		20 - 24	/
		25 - 29	/
		30 - 34	/
	Männlich	35 - 39	/
		40 - 44	/
		45 - 49	/
		50 - 54	/
		55 - 59	/
		60 - 64	/
		65 - 69	/
		70 - 74	/
		75 - 79	/
		80 - 84	/

Noch: Personen nach Alter (5er-Jahresgruppen), Höchster beruflicher Abschluss (ausführlich) und weitere Merkmale für Niedersachsen (Bundesland)

				Niedersachsen (Bundesland)
			85 - 89	/
			90 und älter	/
			Insgesamt	/
			Unter 5	/
			5 - 9	/
			10 - 14	/
			15 - 19	/
			20 - 24	/
			25 - 29	/
			30 - 34	/
			35 - 39	/
			40 - 44	/
		Weiblich	45 - 49	/
			50 - 54	/
			55 - 59	/
			60 - 64	/
			65 - 69	/
			70 - 74	/
			75 - 79	/
			80 - 84	/
			85 - 89	/
			90 und älter	/
			Insgesamt	143 690
			Unter 5	/
			5 - 9	/
			10 - 14	/
			15 - 19	123 240
			20 - 24	17 850
			25 - 29	1 660
			30 - 34	650
			35 - 39	/
		Insgesamt	40 - 44	/
			45 - 49	/
			50 - 54	/
			55 - 59	/
			60 - 64	/
			65 - 69	/
3.2 Schüler/-innen der gymnasialen Oberstufe	Insgesamt		70 - 74	/
			75 - 79	/
			80 - 84	/
			85 - 89	/
			90 und älter	/
			Insgesamt	66 640
			Unter 5	/
			5 - 9	/
			10 - 14	/
			15 - 19	55 760
		Männlich	20 - 24	9 340
			25 - 29	1 050
			30 - 34	/
			35 - 39	/
			40 - 44	/
			45 - 49	/

Noch: Personen nach Alter (5er-Jahresgruppen), Höchster beruflicher Abschluss (ausführlich) und weitere Merkmale für Niedersachsen (Bundesland)

			Niedersachsen (Bundesland)
		50 - 54	/
		55 - 59	/
		60 - 64	/
		65 - 69	/
		70 - 74	/
		75 - 79	/
		80 - 84	/
		85 - 89	/
		90 und älter	/
		Insgesamt	77 050
		Unter 5	/
		5 - 9	/
		10 - 14	/
		15 - 19	67 480
		20 - 24	8 520
		25 - 29	610
		30 - 34	/
		35 - 39	/
		40 - 44	/
	Weiblich	45 - 49	/
		50 - 54	/
		55 - 59	/
		60 - 64	/
		65 - 69	/
		70 - 74	/
		75 - 79	/
		80 - 84	/
		85 - 89	/
		90 und älter	/
		Insgesamt	136 370
		Unter 5	/
		5 - 9	/
		10 - 14	/
		15 - 19	121 730
		20 - 24	13 740
		25 - 29	680
		30 - 34	/
		35 - 39	/
		40 - 44	/
	Insgesamt	45 - 49	/
Ohne beruflichen Abschluss		50 - 54	/
		55 - 59	/
		60 - 64	/
		65 - 69	/
		70 - 74	/
		75 - 79	/
		80 - 84	/
		85 - 89	/
		90 und älter	/
		Insgesamt	62 710
	Männlich	Unter 5	/
		5 - 9	/
		10 - 14	/

Noch: Personen nach Alter (5er-Jahresgruppen), Höchster beruflicher Abschluss (ausführlich) und weitere Merkmale für Niedersachsen (Bundesland)

			Niedersachsen (Bundesland)
		15 - 19	55 210
		20 - 24	7 040
		25 - 29	/
		30 - 34	/
		35 - 39	/
		40 - 44	/
		45 - 49	/
		50 - 54	/
		55 - 59	/
		60 - 64	/
		65 - 69	/
		70 - 74	/
		75 - 79	/
		80 - 84	/
		85 - 89	/
		90 und älter	/
		Insgesamt	73 660
		Unter 5	/
		5 - 9	/
		10 - 14	/
		15 - 19	66 520
		20 - 24	6 700
		25 - 29	/
		30 - 34	/
		35 - 39	/
		40 - 44	/
	Weiblich	45 - 49	/
		50 - 54	/
		55 - 59	/
		60 - 64	/
		65 - 69	/
		70 - 74	/
		75 - 79	/
		80 - 84	/
		85 - 89	/
		90 und älter	/
		Insgesamt	5 580
		Unter 5	/
		5 - 9	/
		10 - 14	/
		15 - 19	770
		20 - 24	3 480
		25 - 29	840
		30 - 34	/
Lehre, Berufsausbildung im dualen System	Insgesamt	35 - 39	/
		40 - 44	/
		45 - 49	/
		50 - 54	/
		55 - 59	/
		60 - 64	/
		65 - 69	/
		70 - 74	/
		75 - 79	/

Noch: Personen nach Alter (5er-Jahresgruppen), Höchster beruflicher Abschluss (ausführlich) und weitere Merkmale für Niedersachsen (Bundesland)

			Niedersachsen (Bundesland)
		80 - 84	/
		85 - 89	/
		90 und älter	/
	Männlich	Insgesamt	3 300
		Unter 5	/
		5 - 9	/
		10 - 14	/
		15 - 19	/
		20 - 24	2 000
		25 - 29	660
		30 - 34	/
		35 - 39	/
		40 - 44	/
		45 - 49	/
		50 - 54	/
		55 - 59	/
		60 - 64	/
		65 - 69	/
		70 - 74	/
		75 - 79	/
		80 - 84	/
		85 - 89	/
		90 und älter	/
	Weiblich	Insgesamt	2 280
		Unter 5	/
		5 - 9	/
		10 - 14	/
		15 - 19	/
		20 - 24	1 480
		25 - 29	/
		30 - 34	/
		35 - 39	/
		40 - 44	/
		45 - 49	/
		50 - 54	/
		55 - 59	/
		60 - 64	/
		65 - 69	/
		70 - 74	/
		75 - 79	/
		80 - 84	/
		85 - 89	/
		90 und älter	/
Fachschulabschluss	Insgesamt	Insgesamt	1 740
		Unter 5	/
		5 - 9	/
		10 - 14	/
		15 - 19	740
		20 - 24	630
		25 - 29	/
		30 - 34	/
		35 - 39	/
		40 - 44	/

Noch: Personen nach Alter (5er-Jahresgruppen), Höchster beruflicher Abschluss (ausführlich) und weitere Merkmale für Niedersachsen (Bundesland)

			Niedersachsen (Bundesland)
		45 - 49	/
		50 - 54	/
		55 - 59	/
		60 - 64	/
		65 - 69	/
		70 - 74	/
		75 - 79	/
		80 - 84	/
		85 - 89	/
		90 und älter	/
	Männlich	Insgesamt	630
		Unter 5	/
		5 - 9	/
		10 - 14	/
		15 - 19	/
		20 - 24	/
		25 - 29	/
		30 - 34	/
		35 - 39	/
		40 - 44	/
		45 - 49	/
		50 - 54	/
		55 - 59	/
		60 - 64	/
		65 - 69	/
		70 - 74	/
		75 - 79	/
		80 - 84	/
		85 - 89	/
		90 und älter	/
	Weiblich	Insgesamt	1 110
		Unter 5	/
		5 - 9	/
		10 - 14	/
		15 - 19	540
		20 - 24	/
		25 - 29	/
		30 - 34	/
		35 - 39	/
		40 - 44	/
		45 - 49	/
		50 - 54	/
		55 - 59	/
		60 - 64	/
		65 - 69	/
		70 - 74	/
		75 - 79	/
		80 - 84	/
		85 - 89	/
		90 und älter	/
Abschluss einer Fachakademie oder Berufsakademie	Insgesamt	Insgesamt	/
		Unter 5	/
		5 - 9	/

Noch: Personen nach Alter (5er-Jahresgruppen), Höchster beruflicher Abschluss (ausführlich) und weitere Merkmale für Niedersachsen (Bundesland)

		Niedersachsen (Bundesland)
	10 - 14	/
	15 - 19	/
	20 - 24	/
	25 - 29	/
	30 - 34	/
	35 - 39	/
	40 - 44	/
	45 - 49	/
	50 - 54	/
	55 - 59	/
	60 - 64	/
	65 - 69	/
	70 - 74	/
	75 - 79	/
	80 - 84	/
	85 - 89	/
	90 und älter	/
Männlich	Insgesamt	/
	Unter 5	/
	5 - 9	/
	10 - 14	/
	15 - 19	/
	20 - 24	/
	25 - 29	/
	30 - 34	/
	35 - 39	/
	40 - 44	/
	45 - 49	/
	50 - 54	/
	55 - 59	/
	60 - 64	/
	65 - 69	/
	70 - 74	/
	75 - 79	/
	80 - 84	/
	85 - 89	/
	90 und älter	/
Weiblich	Insgesamt	/
	Unter 5	/
	5 - 9	/
	10 - 14	/
	15 - 19	/
	20 - 24	/
	25 - 29	/
	30 - 34	/
	35 - 39	/
	40 - 44	/
	45 - 49	/
	50 - 54	/
	55 - 59	/
	60 - 64	/
	65 - 69	/
	70 - 74	/

Noch: Personen nach Alter (5er-Jahresgruppen), Höchster beruflicher Abschluss (ausführlich) und weitere Merkmale für Niedersachsen (Bundesland)

			Niedersachsen (Bundesland)
		75 - 79	/
		80 - 84	/
		85 - 89	/
		90 und älter	/
Fachhochschulabschluss	Insgesamt	Insgesamt	/
		Unter 5	/
		5 - 9	/
		10 - 14	/
		15 - 19	/
		20 - 24	/
		25 - 29	/
		30 - 34	/
		35 - 39	/
		40 - 44	/
		45 - 49	/
		50 - 54	/
		55 - 59	/
		60 - 64	/
		65 - 69	/
		70 - 74	/
		75 - 79	/
		80 - 84	/
		85 - 89	/
		90 und älter	/
	Männlich	Insgesamt	/
		Unter 5	/
		5 - 9	/
		10 - 14	/
		15 - 19	/
		20 - 24	/
		25 - 29	/
		30 - 34	/
		35 - 39	/
		40 - 44	/
		45 - 49	/
		50 - 54	/
		55 - 59	/
		60 - 64	/
		65 - 69	/
		70 - 74	/
		75 - 79	/
		80 - 84	/
		85 - 89	/
		90 und älter	/
	Weiblich	Insgesamt	/
		Unter 5	/
		5 - 9	/
		10 - 14	/
		15 - 19	/
		20 - 24	/
		25 - 29	/
		30 - 34	/
		35 - 39	/

Noch: Personen nach Alter (5er-Jahresgruppen), Höchster beruflicher Abschluss (ausführlich) und weitere Merkmale für Niedersachsen (Bundesland)

			Niedersachsen (Bundesland)
		40 - 44	/
		45 - 49	/
		50 - 54	/
		55 - 59	/
		60 - 64	/
		65 - 69	/
		70 - 74	/
		75 - 79	/
		80 - 84	/
		85 - 89	/
		90 und älter	/
		Insgesamt	/
		Unter 5	/
		5 - 9	/
		10 - 14	/
		15 - 19	/
		20 - 24	/
		25 - 29	/
		30 - 34	/
		35 - 39	/
	Insgesamt	40 - 44	/
		45 - 49	/
		50 - 54	/
		55 - 59	/
		60 - 64	/
		65 - 69	/
		70 - 74	/
		75 - 79	/
		80 - 84	/
		85 - 89	/
		90 und älter	/
Hochschulabschluss		Insgesamt	/
		Unter 5	/
		5 - 9	/
		10 - 14	/
		15 - 19	/
		20 - 24	/
		25 - 29	/
		30 - 34	/
		35 - 39	/
	Männlich	40 - 44	/
		45 - 49	/
		50 - 54	/
		55 - 59	/
		60 - 64	/
		65 - 69	/
		70 - 74	/
		75 - 79	/
		80 - 84	/
		85 - 89	/
		90 und älter	/
	Weiblich	Insgesamt	/
		Unter 5	/

Noch: Personen nach Alter (5er-Jahresgruppen), Höchster beruflicher Abschluss (ausführlich) und weitere Merkmale für Niedersachsen (Bundesland)

			Niedersachsen (Bundesland)
		5 - 9	/
		10 - 14	/
		15 - 19	/
		20 - 24	/
		25 - 29	/
		30 - 34	/
		35 - 39	/
		40 - 44	/
		45 - 49	/
		50 - 54	/
		55 - 59	/
		60 - 64	/
		65 - 69	/
		70 - 74	/
		75 - 79	/
		80 - 84	/
		85 - 89	/
		90 und älter	/
		Insgesamt	/
		Unter 5	/
		5 - 9	/
		10 - 14	/
		15 - 19	/
		20 - 24	/
		25 - 29	/
		30 - 34	/
		35 - 39	/
	Insgesamt	40 - 44	/
		45 - 49	/
		50 - 54	/
		55 - 59	/
		60 - 64	/
		65 - 69	/
		70 - 74	/
		75 - 79	/
Promotion		80 - 84	/
		85 - 89	/
		90 und älter	/
		Insgesamt	/
		Unter 5	/
		5 - 9	/
		10 - 14	/
		15 - 19	/
		20 - 24	/
		25 - 29	/
	Männlich	30 - 34	/
		35 - 39	/
		40 - 44	/
		45 - 49	/
		50 - 54	/
		55 - 59	/
		60 - 64	/
		65 - 69	/

Noch: Personen nach Alter (5er-Jahresgruppen), Höchster beruflicher Abschluss (ausführlich) und weitere Merkmale für Niedersachsen (Bundesland)

				Niedersachsen (Bundesland)
			70 - 74	/
			75 - 79	/
			80 - 84	/
			85 - 89	/
			90 und älter	/
		Weiblich	Insgesamt	/
			Unter 5	/
			5 - 9	/
			10 - 14	/
			15 - 19	/
			20 - 24	/
			25 - 29	/
			30 - 34	/
			35 - 39	/
			40 - 44	/
			45 - 49	/
			50 - 54	/
			55 - 59	/
			60 - 64	/
			65 - 69	/
			70 - 74	/
			75 - 79	/
			80 - 84	/
			85 - 89	/
			90 und älter	/
4 Fachhochschulreife	Insgesamt	Insgesamt	Insgesamt	523 840
			Unter 5	/
			5 - 9	/
			10 - 14	/
			15 - 19	4 430
			20 - 24	41 270
			25 - 29	48 880
			30 - 34	49 580
			35 - 39	47 550
			40 - 44	63 300
			45 - 49	64 410
			50 - 54	52 740
			55 - 59	41 470
			60 - 64	35 100
			65 - 69	26 870
			70 - 74	23 790
			75 - 79	12 440
			80 - 84	6 530
			85 - 89	4 120
			90 und älter	1 360
		Männlich	Insgesamt	315 480
			Unter 5	/
			5 - 9	/
			10 - 14	/
			15 - 19	1 870
			20 - 24	19 400
			25 - 29	24 410
			30 - 34	25 670

Noch: Personen nach Alter (5er-Jahresgruppen), Höchster beruflicher Abschluss (ausführlich) und weitere Merkmale für Niedersachsen (Bundesland)

				Niedersachsen (Bundesland)
			35 - 39	27 740
			40 - 44	37 750
			45 - 49	39 440
			50 - 54	32 640
			55 - 59	26 440
			60 - 64	24 730
			65 - 69	20 710
			70 - 74	17 690
			75 - 79	9 270
			80 - 84	4 520
			85 - 89	2 550
			90 und älter	660
		Weiblich	Insgesamt	208 360
			Unter 5	/
			5 - 9	/
			10 - 14	/
			15 - 19	2 560
			20 - 24	21 870
			25 - 29	24 470
			30 - 34	23 920
			35 - 39	19 800
			40 - 44	25 550
			45 - 49	24 970
			50 - 54	20 100
			55 - 59	15 030
			60 - 64	10 380
			65 - 69	6 170
			70 - 74	6 100
			75 - 79	3 170
			80 - 84	2 010
			85 - 89	1 560
			90 und älter	700
	Ohne beruflichen Abschluss	Insgesamt	Insgesamt	41 480
			Unter 5	/
			5 - 9	/
			10 - 14	/
			15 - 19	3 900
			20 - 24	16 060
			25 - 29	4 490
			30 - 34	2 700
			35 - 39	2 810
			40 - 44	2 880
			45 - 49	2 440
			50 - 54	2 120
			55 - 59	1 330
			60 - 64	750
			65 - 69	610
			70 - 74	650
			75 - 79	/
			80 - 84	/
			85 - 89	/
			90 und älter	/
		Männlich	Insgesamt	21 000

Noch: Personen nach Alter (5er-Jahresgruppen), Höchster beruflicher Abschluss (ausführlich) und weitere Merkmale für Niedersachsen (Bundesland)

			Niedersachsen (Bundesland)
		Unter 5	/
		5 - 9	/
		10 - 14	/
		15 - 19	1 630
		20 - 24	8 180
		25 - 29	2 560
		30 - 34	1 240
		35 - 39	1 480
		40 - 44	1 690
		45 - 49	1 520
		50 - 54	1 140
		55 - 59	660
		60 - 64	/
		65 - 69	/
		70 - 74	/
		75 - 79	/
		80 - 84	/
		85 - 89	/
		90 und älter	/
		Insgesamt	20 480
	Weiblich	Unter 5	/
		5 - 9	/
		10 - 14	/
		15 - 19	2 270
		20 - 24	7 880
		25 - 29	1 940
		30 - 34	1 460
		35 - 39	1 330
		40 - 44	1 190
		45 - 49	920
		50 - 54	980
		55 - 59	670
		60 - 64	/
		65 - 69	380
		70 - 74	400
		75 - 79	/
		80 - 84	/
		85 - 89	/
		90 und älter	/
		Insgesamt	129 470
Lehre, Berufsausbildung im dualen System	Insgesamt	Unter 5	/
		5 - 9	/
		10 - 14	/
		15 - 19	/
		20 - 24	14 580
		25 - 29	21 550
		30 - 34	16 750
		35 - 39	14 810
		40 - 44	16 850
		45 - 49	16 340
		50 - 54	10 960
		55 - 59	6 480
		60 - 64	3 460

Noch: Personen nach Alter (5er-Jahresgruppen), Höchster beruflicher Abschluss (ausführlich) und weitere Merkmale für Niedersachsen (Bundesland)

			Niedersachsen (Bundesland)
		65 - 69	2 430
		70 - 74	2 280
		75 - 79	1 160
		80 - 84	640
		85 - 89	630
		90 und älter	/
		Insgesamt	67 680
	Männlich	Unter 5	/
		5 - 9	/
		10 - 14	/
		15 - 19	/
		20 - 24	7 320
		25 - 29	10 640
		30 - 34	8 320
		35 - 39	7 810
		40 - 44	8 270
		45 - 49	8 440
		50 - 54	6 370
		55 - 59	4 140
		60 - 64	2 230
		65 - 69	1 470
		70 - 74	1 160
		75 - 79	590
		80 - 84	380
		85 - 89	/
		90 und älter	/
		Insgesamt	61 790
	Weiblich	Unter 5	/
		5 - 9	/
		10 - 14	/
		15 - 19	/
		20 - 24	7 260
		25 - 29	10 910
		30 - 34	8 430
		35 - 39	7 000
		40 - 44	8 580
		45 - 49	7 900
		50 - 54	4 580
		55 - 59	2 330
		60 - 64	1 240
		65 - 69	950
		70 - 74	1 120
		75 - 79	570
		80 - 84	/
		85 - 89	/
		90 und älter	/
		Insgesamt	108 380
Fachschulabschluss	Insgesamt	Unter 5	/
		5 - 9	/
		10 - 14	/
		15 - 19	/
		20 - 24	5 600
		25 - 29	10 350

Noch: Personen nach Alter (5er-Jahresgruppen), Höchster beruflicher Abschluss (ausführlich) und weitere Merkmale für Niedersachsen (Bundesland)

		Niedersachsen (Bundesland)
	30 - 34	12 060
	35 - 39	11 090
	40 - 44	16 690
	45 - 49	16 060
	50 - 54	12 340
	55 - 59	7 970
	60 - 64	6 470
	65 - 69	3 440
	70 - 74	3 240
	75 - 79	1 510
	80 - 84	800
	85 - 89	440
	90 und älter	/
Männlich	Insgesamt	63 470
	Unter 5	/
	5 - 9	/
	10 - 14	/
	15 - 19	/
	20 - 24	1 920
	25 - 29	4 680
	30 - 34	6 290
	35 - 39	6 910
	40 - 44	10 590
	45 - 49	10 010
	50 - 54	7 390
	55 - 59	4 770
	60 - 64	4 390
	65 - 69	2 590
	70 - 74	2 060
	75 - 79	1 040
	80 - 84	390
	85 - 89	/
	90 und älter	/
Weiblich	Insgesamt	44 910
	Unter 5	/
	5 - 9	/
	10 - 14	/
	15 - 19	/
	20 - 24	3 670
	25 - 29	5 670
	30 - 34	5 770
	35 - 39	4 170
	40 - 44	6 100
	45 - 49	6 040
	50 - 54	4 950
	55 - 59	3 210
	60 - 64	2 070
	65 - 69	850
	70 - 74	1 180
	75 - 79	470
	80 - 84	400
	85 - 89	/
	90 und älter	/

Noch: Personen nach Alter (5er-Jahresgruppen), Höchster beruflicher Abschluss (ausführlich) und weitere Merkmale für Niedersachsen (Bundesland)

			Niedersachsen (Bundesland)
Abschluss einer Fachakademie oder Berufsakademie	Insgesamt	Insgesamt	51 140
		Unter 5	/
		5 - 9	/
		10 - 14	/
		15 - 19	/
		20 - 24	1 340
		25 - 29	2 480
		30 - 34	3 750
		35 - 39	4 510
		40 - 44	6 360
		45 - 49	6 900
		50 - 54	5 610
		55 - 59	5 060
		60 - 64	5 310
		65 - 69	3 680
		70 - 74	2 970
		75 - 79	1 600
		80 - 84	920
		85 - 89	450
		90 und älter	/
	Männlich	Insgesamt	28 990
		Unter 5	/
		5 - 9	/
		10 - 14	/
		15 - 19	/
		20 - 24	440
		25 - 29	1 150
		30 - 34	2 020
		35 - 39	2 290
		40 - 44	3 190
		45 - 49	4 050
		50 - 54	3 220
		55 - 59	3 070
		60 - 64	3 160
		65 - 69	2 640
		70 - 74	1 970
		75 - 79	1 090
		80 - 84	510
		85 - 89	/
		90 und älter	/
	Weiblich	Insgesamt	22 160
		Unter 5	/
		5 - 9	/
		10 - 14	/
		15 - 19	/
		20 - 24	900
		25 - 29	1 340
		30 - 34	1 730
		35 - 39	2 220
		40 - 44	3 170
		45 - 49	2 850
		50 - 54	2 390
		55 - 59	1 990

Noch: Personen nach Alter (5er-Jahresgruppen), Höchster beruflicher Abschluss (ausführlich) und weitere Merkmale für Niedersachsen (Bundesland)

			Niedersachsen (Bundesland)
		60 - 64	2 150
		65 - 69	1 040
		70 - 74	1 000
		75 - 79	500
		80 - 84	410
		85 - 89	/
		90 und älter	/
Fachhochschulabschluss	Insgesamt	Insgesamt	173 940
		Unter 5	/
		5 - 9	/
		10 - 14	/
		15 - 19	/
		20 - 24	3 320
		25 - 29	8 380
		30 - 34	11 880
		35 - 39	12 640
		40 - 44	18 890
		45 - 49	20 500
		50 - 54	19 900
		55 - 59	18 550
		60 - 64	17 120
		65 - 69	15 350
		70 - 74	13 730
		75 - 79	7 200
		80 - 84	3 680
		85 - 89	2 220
		90 und älter	560
	Männlich	Insgesamt	124 010
		Unter 5	/
		5 - 9	/
		10 - 14	/
		15 - 19	/
		20 - 24	1 450
		25 - 29	4 730
		30 - 34	6 710
		35 - 39	8 580
		40 - 44	13 280
		45 - 49	14 180
		50 - 54	13 600
		55 - 59	12 370
		60 - 64	13 380
		65 - 69	12 800
		70 - 74	11 760
		75 - 79	6 030
		80 - 84	2 980
		85 - 89	1 700
		90 und älter	460
	Weiblich	Insgesamt	49 930
		Unter 5	/
		5 - 9	/
		10 - 14	/
		15 - 19	/
		20 - 24	1 870

Noch: Personen nach Alter (5er-Jahresgruppen), Höchster beruflicher Abschluss (ausführlich) und weitere Merkmale für Niedersachsen (Bundesland)

			Niedersachsen (Bundesland)
		25 - 29	3 650
		30 - 34	5 170
		35 - 39	4 060
		40 - 44	5 610
		45 - 49	6 330
		50 - 54	6 310
		55 - 59	6 180
		60 - 64	3 740
		65 - 69	2 550
		70 - 74	1 970
		75 - 79	1 170
		80 - 84	700
		85 - 89	520
		90 und älter	/
Hochschulabschluss	Insgesamt	Insgesamt	18 060
		Unter 5	/
		5 - 9	/
		10 - 14	/
		15 - 19	/
		20 - 24	/
		25 - 29	1 610
		30 - 34	2 260
		35 - 39	1 500
		40 - 44	1 530
		45 - 49	2 070
		50 - 54	1 740
		55 - 59	1 970
		60 - 64	1 850
		65 - 69	1 260
		70 - 74	820
		75 - 79	630
		80 - 84	/
		85 - 89	/
		90 und älter	/
	Männlich	Insgesamt	9 460
		Unter 5	/
		5 - 9	/
		10 - 14	/
		15 - 19	/
		20 - 24	/
		25 - 29	650
		30 - 34	1 010
		35 - 39	550
		40 - 44	680
		45 - 49	1 180
		50 - 54	860
		55 - 59	1 370
		60 - 64	1 190
		65 - 69	880
		70 - 74	420
		75 - 79	/
		80 - 84	/
		85 - 89	/

Noch: Personen nach Alter (5er-Jahresgruppen), Höchster beruflicher Abschluss (ausführlich) und weitere Merkmale für Niedersachsen (Bundesland)

			Niedersachsen (Bundesland)
		90 und älter	/
	Weiblich	Insgesamt	8 610
		Unter 5	/
		5 - 9	/
		10 - 14	/
		15 - 19	/
		20 - 24	/
		25 - 29	960
		30 - 34	1 250
		35 - 39	960
		40 - 44	850
		45 - 49	890
		50 - 54	870
		55 - 59	600
		60 - 64	670
		65 - 69	/
		70 - 74	400
		75 - 79	/
		80 - 84	/
		85 - 89	/
		90 und älter	/
Promotion	Insgesamt	Insgesamt	1 360
		Unter 5	/
		5 - 9	/
		10 - 14	/
		15 - 19	/
		20 - 24	/
		25 - 29	/
		30 - 34	/
		35 - 39	/
		40 - 44	/
		45 - 49	/
		50 - 54	/
		55 - 59	/
		60 - 64	/
		65 - 69	/
		70 - 74	/
		75 - 79	/
		80 - 84	/
		85 - 89	/
		90 und älter	/
	Männlich	Insgesamt	870
		Unter 5	/
		5 - 9	/
		10 - 14	/
		15 - 19	/
		20 - 24	/
		25 - 29	/
		30 - 34	/
		35 - 39	/
		40 - 44	/
		45 - 49	/
		50 - 54	/

Noch: Personen nach Alter (5er-Jahresgruppen), Höchster beruflicher Abschluss (ausführlich) und weitere Merkmale für Niedersachsen (Bundesland)

				Niedersachsen (Bundesland)
			55 - 59	/
			60 - 64	/
			65 - 69	/
			70 - 74	/
			75 - 79	/
			80 - 84	/
			85 - 89	/
			90 und älter	/
		Weiblich	Insgesamt	/
			Unter 5	/
			5 - 9	/
			10 - 14	/
			15 - 19	/
			20 - 24	/
			25 - 29	/
			30 - 34	/
			35 - 39	/
			40 - 44	/
			45 - 49	/
			50 - 54	/
			55 - 59	/
			60 - 64	/
			65 - 69	/
			70 - 74	/
			75 - 79	/
			80 - 84	/
			85 - 89	/
			90 und älter	/
5 Allg./fachgebundene Hochschulreife (Abitur)	Insgesamt	Insgesamt	Insgesamt	1 123 670
			Unter 5	/
			5 - 9	/
			10 - 14	/
			15 - 19	9 790
			20 - 24	128 740
			25 - 29	118 000
			30 - 34	110 910
			35 - 39	108 570
			40 - 44	132 440
			45 - 49	137 050
			50 - 54	107 980
			55 - 59	80 490
			60 - 64	59 550
			65 - 69	42 830
			70 - 74	36 410
			75 - 79	20 500
			80 - 84	17 130
			85 - 89	9 860
			90 und älter	3 420
		Männlich	Insgesamt	558 520
			Unter 5	/
			5 - 9	/
			10 - 14	/
			15 - 19	3 540

Noch: Personen nach Alter (5er-Jahresgruppen), Höchster beruflicher Abschluss (ausführlich) und weitere Merkmale für Niedersachsen (Bundesland)

			Niedersachsen (Bundesland)
		20 - 24	56 620
		25 - 29	52 260
		30 - 34	50 080
		35 - 39	52 590
		40 - 44	63 890
		45 - 49	69 070
		50 - 54	55 070
		55 - 59	43 960
		60 - 64	34 660
		65 - 69	25 900
		70 - 74	22 340
		75 - 79	12 900
		80 - 84	9 070
		85 - 89	5 080
		90 und älter	1 490
	Weiblich	Insgesamt	565 160
		Unter 5	/
		5 - 9	/
		10 - 14	/
		15 - 19	6 250
		20 - 24	72 110
		25 - 29	65 740
		30 - 34	60 830
		35 - 39	55 990
		40 - 44	68 550
		45 - 49	67 980
		50 - 54	52 920
		55 - 59	36 530
		60 - 64	24 890
		65 - 69	16 930
		70 - 74	14 070
		75 - 79	7 600
		80 - 84	8 070
		85 - 89	4 780
		90 und älter	1 930
Ohne beruflichen Abschluss	Insgesamt	Insgesamt	187 890
		Unter 5	/
		5 - 9	/
		10 - 14	/
		15 - 19	9 600
		20 - 24	95 150
		25 - 29	24 600
		30 - 34	9 790
		35 - 39	7 500
		40 - 44	8 080
		45 - 49	9 360
		50 - 54	7 710
		55 - 59	4 780
		60 - 64	2 570
		65 - 69	1 870
		70 - 74	1 770
		75 - 79	1 060
		80 - 84	1 950

Noch: Personen nach Alter (5er-Jahresgruppen), Höchster beruflicher Abschluss (ausführlich) und weitere Merkmale für Niedersachsen (Bundesland)

			Niedersachsen (Bundesland)
		85 - 89	1 700
		90 und älter	420
	Männlich	Insgesamt	91 500
		Unter 5	/
		5 - 9	/
		10 - 14	/
		15 - 19	3 470
		20 - 24	43 910
		25 - 29	14 550
		30 - 34	5 640
		35 - 39	4 190
		40 - 44	4 350
		45 - 49	5 020
		50 - 54	4 060
		55 - 59	2 420
		60 - 64	1 120
		65 - 69	900
		70 - 74	560
		75 - 79	/
		80 - 84	410
		85 - 89	590
		90 und älter	/
	Weiblich	Insgesamt	96 400
		Unter 5	/
		5 - 9	/
		10 - 14	/
		15 - 19	6 130
		20 - 24	51 240
		25 - 29	10 050
		30 - 34	4 140
		35 - 39	3 300
		40 - 44	3 720
		45 - 49	4 340
		50 - 54	3 650
		55 - 59	2 360
		60 - 64	1 450
		65 - 69	970
		70 - 74	1 210
		75 - 79	810
		80 - 84	1 540
		85 - 89	1 110
		90 und älter	370
Lehre, Berufsausbildung im dualen System	Insgesamt	Insgesamt	182 970
		Unter 5	/
		5 - 9	/
		10 - 14	/
		15 - 19	/
		20 - 24	16 610
		25 - 29	25 050
		30 - 34	21 030
		35 - 39	22 270
		40 - 44	27 450
		45 - 49	27 030

Noch: Personen nach Alter (5er-Jahresgruppen), Höchster beruflicher Abschluss (ausführlich) und weitere Merkmale für Niedersachsen (Bundesland)

			Niedersachsen (Bundesland)
		50 - 54	16 890
		55 - 59	8 320
		60 - 64	4 270
		65 - 69	2 980
		70 - 74	3 210
		75 - 79	2 890
		80 - 84	2 630
		85 - 89	1 430
		90 und älter	740
	Männlich	Insgesamt	76 760
		Unter 5	/
		5 - 9	/
		10 - 14	/
		15 - 19	/
		20 - 24	6 460
		25 - 29	10 340
		30 - 34	8 200
		35 - 39	9 280
		40 - 44	10 380
		45 - 49	10 520
		50 - 54	7 380
		55 - 59	4 710
		60 - 64	2 330
		65 - 69	1 620
		70 - 74	1 790
		75 - 79	1 370
		80 - 84	1 230
		85 - 89	780
		90 und älter	/
	Weiblich	Insgesamt	106 200
		Unter 5	/
		5 - 9	/
		10 - 14	/
		15 - 19	/
		20 - 24	10 150
		25 - 29	14 710
		30 - 34	12 820
		35 - 39	12 990
		40 - 44	17 070
		45 - 49	16 510
		50 - 54	9 510
		55 - 59	3 620
		60 - 64	1 940
		65 - 69	1 360
		70 - 74	1 430
		75 - 79	1 520
		80 - 84	1 410
		85 - 89	650
		90 und älter	410
Fachschulabschluss	Insgesamt	Insgesamt	90 540
		Unter 5	/
		5 - 9	/
		10 - 14	/

Noch: Personen nach Alter (5er-Jahresgruppen), Höchster beruflicher Abschluss (ausführlich) und weitere Merkmale für Niedersachsen (Bundesland)

		Niedersachsen (Bundesland)
	15 - 19	/
	20 - 24	3 770
	25 - 29	8 830
	30 - 34	9 650
	35 - 39	10 260
	40 - 44	13 990
	45 - 49	14 700
	50 - 54	10 620
	55 - 59	6 290
	60 - 64	3 650
	65 - 69	2 190
	70 - 74	1 940
	75 - 79	1 470
	80 - 84	1 790
	85 - 89	1 030
	90 und älter	320
Männlich	Insgesamt	34 250
	Unter 5	/
	5 - 9	/
	10 - 14	/
	15 - 19	/
	20 - 24	960
	25 - 29	2 640
	30 - 34	3 540
	35 - 39	3 830
	40 - 44	5 530
	45 - 49	5 840
	50 - 54	4 350
	55 - 59	2 860
	60 - 64	1 680
	65 - 69	800
	70 - 74	750
	75 - 79	500
	80 - 84	550
	85 - 89	/
	90 und älter	/
Weiblich	Insgesamt	56 290
	Unter 5	/
	5 - 9	/
	10 - 14	/
	15 - 19	/
	20 - 24	2 800
	25 - 29	6 190
	30 - 34	6 110
	35 - 39	6 430
	40 - 44	8 460
	45 - 49	8 860
	50 - 54	6 270
	55 - 59	3 440
	60 - 64	1 970
	65 - 69	1 400
	70 - 74	1 190
	75 - 79	970

Noch: Personen nach Alter (5er-Jahresgruppen), Höchster beruflicher Abschluss (ausführlich) und weitere Merkmale für Niedersachsen (Bundesland)

			Niedersachsen (Bundesland)
		80 - 84	1 230
		85 - 89	700
		90 und älter	/
Abschluss einer Fachakademie oder Berufsakademie	Insgesamt	Insgesamt	30 990
		Unter 5	/
		5 - 9	/
		10 - 14	/
		15 - 19	/
		20 - 24	1 590
		25 - 29	3 480
		30 - 34	4 470
		35 - 39	4 230
		40 - 44	5 040
		45 - 49	3 950
		50 - 54	2 570
		55 - 59	1 390
		60 - 64	1 230
		65 - 69	1 130
		70 - 74	770
		75 - 79	340
		80 - 84	300
		85 - 89	430
		90 und älter	/
	Männlich	Insgesamt	14 990
		Unter 5	/
		5 - 9	/
		10 - 14	/
		15 - 19	/
		20 - 24	850
		25 - 29	1 250
		30 - 34	1 700
		35 - 39	2 260
		40 - 44	2 620
		45 - 49	2 050
		50 - 54	1 360
		55 - 59	740
		60 - 64	490
		65 - 69	670
		70 - 74	400
		75 - 79	/
		80 - 84	/
		85 - 89	/
		90 und älter	/
	Weiblich	Insgesamt	16 000
		Unter 5	/
		5 - 9	/
		10 - 14	/
		15 - 19	/
		20 - 24	730
		25 - 29	2 230
		30 - 34	2 770
		35 - 39	1 970
		40 - 44	2 420

Noch: Personen nach Alter (5er-Jahresgruppen), Höchster beruflicher Abschluss (ausführlich) und weitere Merkmale für Niedersachsen (Bundesland)

			Niedersachsen (Bundesland)
		45 - 49	1 890
		50 - 54	1 210
		55 - 59	650
		60 - 64	740
		65 - 69	460
		70 - 74	/
		75 - 79	/
		80 - 84	/
		85 - 89	/
		90 und älter	/
Fachhochschulabschluss	Insgesamt	Insgesamt	152 080
		Unter 5	/
		5 - 9	/
		10 - 14	/
		15 - 19	/
		20 - 24	2 310
		25 - 29	13 270
		30 - 34	18 170
		35 - 39	17 670
		40 - 44	21 530
		45 - 49	23 440
		50 - 54	19 120
		55 - 59	12 080
		60 - 64	7 850
		65 - 69	5 370
		70 - 74	5 220
		75 - 79	2 690
		80 - 84	1 890
		85 - 89	1 120
		90 und älter	370
	Männlich	Insgesamt	89 790
		Unter 5	/
		5 - 9	/
		10 - 14	/
		15 - 19	/
		20 - 24	1 080
		25 - 29	6 620
		30 - 34	10 130
		35 - 39	10 160
		40 - 44	12 100
		45 - 49	14 550
		50 - 54	11 260
		55 - 59	7 180
		60 - 64	5 410
		65 - 69	3 420
		70 - 74	3 600
		75 - 79	2 080
		80 - 84	1 260
		85 - 89	730
		90 und älter	/
	Weiblich	Insgesamt	62 290
		Unter 5	/
		5 - 9	/

Noch: Personen nach Alter (5er-Jahresgruppen), Höchster beruflicher Abschluss (ausführlich) und weitere Merkmale für Niedersachsen (Bundesland)

			Niedersachsen (Bundesland)
		10 - 14	/
		15 - 19	/
		20 - 24	1 230
		25 - 29	6 640
		30 - 34	8 040
		35 - 39	7 510
		40 - 44	9 430
		45 - 49	8 900
		50 - 54	7 860
		55 - 59	4 900
		60 - 64	2 440
		65 - 69	1 950
		70 - 74	1 620
		75 - 79	600
		80 - 84	620
		85 - 89	380
		90 und älter	/
		Insgesamt	413 810
Hochschulabschluss	Insgesamt	Unter 5	/
		5 - 9	/
		10 - 14	/
		15 - 19	/
		20 - 24	9 160
		25 - 29	41 400
		30 - 34	42 840
		35 - 39	39 480
		40 - 44	48 350
		45 - 49	49 270
		50 - 54	42 960
		55 - 59	41 200
		60 - 64	34 850
		65 - 69	24 750
		70 - 74	19 080
		75 - 79	9 300
		80 - 84	6 610
		85 - 89	3 440
		90 und älter	1 120
		Insgesamt	206 050
	Männlich	Unter 5	/
		5 - 9	/
		10 - 14	/
		15 - 19	/
		20 - 24	3 210
		25 - 29	16 370
		30 - 34	18 460
		35 - 39	18 710
		40 - 44	23 520
		45 - 49	24 950
		50 - 54	21 120
		55 - 59	21 200
		60 - 64	19 470
		65 - 69	14 800
		70 - 74	11 610

Noch: Personen nach Alter (5er-Jahresgruppen), Höchster beruflicher Abschluss (ausführlich) und weitere Merkmale für Niedersachsen (Bundesland)

			Niedersachsen (Bundesland)
		75 - 79	6 270
		80 - 84	3 890
		85 - 89	1 940
		90 und älter	530
		Insgesamt	207 760
	Weiblich	Unter 5	/
		5 - 9	/
		10 - 14	/
		15 - 19	/
		20 - 24	5 950
		25 - 29	25 030
		30 - 34	24 380
		35 - 39	20 770
		40 - 44	24 820
		45 - 49	24 320
		50 - 54	21 840
		55 - 59	19 990
		60 - 64	15 380
		65 - 69	9 950
		70 - 74	7 460
		75 - 79	3 030
		80 - 84	2 720
		85 - 89	1 500
		90 und älter	590
		Insgesamt	65 390
Promotion	Insgesamt	Unter 5	/
		5 - 9	/
		10 - 14	/
		15 - 19	/
		20 - 24	/
		25 - 29	1 370
		30 - 34	4 960
		35 - 39	7 170
		40 - 44	8 010
		45 - 49	9 290
		50 - 54	8 110
		55 - 59	6 430
		60 - 64	5 140
		65 - 69	4 540
		70 - 74	4 420
		75 - 79	2 750
		80 - 84	1 970
		85 - 89	710
		90 und älter	380
		Insgesamt	45 170
	Männlich	Unter 5	/
		5 - 9	/
		10 - 14	/
		15 - 19	/
		20 - 24	/
		25 - 29	480
		30 - 34	2 390
		35 - 39	4 160

Noch: Personen nach Alter (5er-Jahresgruppen), Höchster beruflicher Abschluss (ausführlich) und weitere Merkmale für Niedersachsen (Bundesland)

		Niedersachsen (Bundesland)
	40 - 44	5 380
	45 - 49	6 130
	50 - 54	5 540
	55 - 59	4 850
	60 - 64	4 170
	65 - 69	3 700
	70 - 74	3 640
	75 - 79	2 270
	80 - 84	1 590
	85 - 89	480
	90 und älter	/
	Insgesamt	20 210
Weiblich	Unter 5	/
	5 - 9	/
	10 - 14	/
	15 - 19	/
	20 - 24	/
	25 - 29	890
	30 - 34	2 570
	35 - 39	3 010
	40 - 44	2 630
	45 - 49	3 160
	50 - 54	2 580
	55 - 59	1 580
	60 - 64	970
	65 - 69	840
	70 - 74	780
	75 - 79	480
	80 - 84	380
	85 - 89	/
	90 und älter	/

Für die Bevölkerung in bestimmten Gemeinschafts- und Anstaltsunterkünften (sog. „sensible Sonderbereiche") liegen keine Informationen zu sozioökonomischen Strukturmerkmalen (beispielsweise Schul- und Berufsbildung) oder zum Migrationshintergrund vor. Aus diesem Grund können die Summen der Merkmalsausprägungen von der Gesamteinwohnerzahl der ausgewählten regionalen Einheit abweichen.

Die Berechnung der Ergebnisse zum „Erwerbsstatus“, zur „Stellung im Beruf“ und zum „Wirtschaftszweig“ erfolgte durch kombinierte Erwerbsregisterauszählung und Hochrechnung der Daten aus der Haushaltsstichprobe. Daher weichen diese von den ausschließlich aus der Haushaltsstichprobe hochgerechneten Ergebnissen ab.

Im Ausland tätige Angehörige der Bundeswehr, der Polizeibehörden und des Auswärtigen Dienstes sowie ihre dort ansässigen Familien werden für dieses Ergebnis nicht berücksichtigt.

Die dargestellten Summenwerte werden stets auf Basis der ungerundeten Ausgangswerte ermittelt, weshalb diese von der Summe der ausgewiesenen Einzelwerte abweichen können.

Die relativen Ergebnisse beziehen sich auf die jeweilige Teilgesamtheit des betrachteten Merkmals der jeweiligen regionalen Einheit. Die relativen Werte wurden durch Division von Ergebnissen nach Durchführung der Geheimhaltung errechnet.

Beim Nachweis der Bevölkerung nach "Höchstem beruflichen Abschluss (ausführlich)" werden ausschließlich Personen im Alter von 15 Jahren und älter berücksichtigt.

Beim Nachweis der Bevölkerung nach "Höchstem Schulabschluss" werden ausschließlich Personen im Alter von 15 Jahren und älter berücksichtigt.

© Statistische Ämter des Bundes und der Länder, 2014
Vervielfältigung und Verbreitung mit Quellenangabe gestattet.

Zensus 9. Mai 2011

Einführung

Der Zensus 2011 ist eine Bevölkerungs-, Gebäude- und Wohnungszählung. Dabei werden – soweit möglich – bereits vorhandene Daten aus Verwaltungsregistern für statistische Zwecke genutzt. Eine Haushaltebefragung auf Stichprobenbasis, eine Vollerhebung aller an Anschriften mit Sonderbereichen lebenden Personen (Wohnheime und Gemeinschaftsunterkünfte) sowie eine postalische Befragung zu Gebäude- und Wohnungsdaten bei den Eigentümerinnen und Eigentümern oder Verwalterinnen und Verwaltern ergänzen und korrigieren die Informationen aus den Registern. Eine traditionelle Volkszählung inklusive einer Gebäude- und Wohnungszählung wurde in den alten Bundesländern letztmalig 1987, in der DDR letztmalig 1981 durchgeführt. Eine Gebäude- und Wohnungszählung fand nach der Wiedervereinigung nur in den neuen Bundesländern im Jahr 1995 statt.

Ziel des Zensus 2011 war zum einen die Ermittlung der amtlichen Einwohnerzahlen zum Stichtag 9. Mai 2011. Zum anderen wurden wichtige Strukturinformationen erhoben, welche eine umfassende Datengrundlage bieten, wie die Menschen in Deutschland leben, wohnen und arbeiten.

Die Zensusdatenbank präsentiert Ergebnisse des Zensus 2011 nach Abschluss der Datenaufbereitung.

Rechtliche Grundlagen

Die rechtlichen Grundlagen des Zensus 2011 bilden

- die Verordnung Europäische Gemeinschaft (EG) Nummer 763/2008 des Europäischen Parlaments und des Rates vom 9. Juli 2008 über Volks- und Wohnungszählungen,
- das Zensusvorbereitungsgesetz (ZensVorbG 2011),
- das Zensusgesetz 2011 (ZensG 2011),
- die Stichprobenverordnung Zensusgesetz 2011 (StichprobenV),
- sowie die jeweiligen landesrechtlichen Regelungen zur Ausführung des ZensG 2011.

Nach § 1 Abs. 1 ZensG 2011 führten die Statistischen Ämter des Bundes und der Länder eine Bevölkerungs-, Gebäude- und Wohnungszählung (Zensus) mit Stand zum 9. Mai 2011 (Berichtszeitpunkt) als Bundesstatistik durch.

Gemäß § 1 Abs. 3 ZensG 2011 dient der Zensus unter anderem der Gewinnung von Grunddaten für das Gesamtsystem der amtlichen Statistik sowie von Strukturdaten über die Bevölkerung als Datengrundlage für vielfältige politische Entscheidungen.

Die nachstehend veröffentlichten Ergebnisse wurden auf Basis folgender Register, Erhebungen und statistischer Verfahren ermittelt:

- Daten gemäß Übermittlung durch die Meldebehörden und durch oberste Bundesbehörden (§ 3 ZensG 2011),
- Daten gemäß Übermittlung durch die Bundesagentur für Arbeit (§ 4 ZensG 2011),
- Daten gemäß Übermittlung durch die nach dem Finanz- und Personalstatistikgesetz auskunftspflichtigen Stellen (§ 5 ZensG 2011),
- Daten aus der Gebäude- und Wohnungszählung (§ 6 ZensG 2011),
- Daten aus der Haushaltebefragung auf Stichprobenbasis (§ 7 ZensG 2011),
- Daten aus der Erhebung an Anschriften mit Sonderbereichen (§ 8 ZensG 2011),
- Daten aus der Zusammenführung der Datensätze und Haushaltegenerierung (§ 9 Abs. 3 ZensG 2011),
- Daten aus der Mehrfachfalluntersuchung (§ 15 ZensG 2011),
- Daten aus der Befragung zur Klärung von Unstimmigkeiten (§ 16 ZensG 2011).

Methode

Beim Zensus 2011 kam in Deutschland erstmals ein neues registergestütztes Verfahren zum Einsatz. Dabei wurden bereits vorhandene Registerdaten verwendet und mit den Ergebnissen unterschiedlicher Befragungen ergänzt und korrigiert. Zur Gewinnung von Angaben, die nicht aus Registern gewonnen werden konnten sowie zur Sicherung der Qualität der Ergebnisse, wurden bundesweit knapp zehn Prozent aller Personen im Rahmen der Haushaltebefragung auf Stichprobenbasis befragt. Dafür wurden nach einem mathematischen Zufallsverfahren Anschriften ausgewählt, an denen alle dort lebenden Personen befragt wurden.

Da es in Deutschland zu Gebäude- und Wohnungsdaten keine flächendeckenden Register gibt, wurden die Angaben zu Gebäuden und Wohnungen durch eine postalische Befragung der Eigentümerinnen und Eigentümer oder Verwalterinnen und Verwalter von Gebäuden und Wohnungen sowie bewohnten Unterkünften gewonnen. Die zum Stichtag 9. Mai 2011 als Vollerhebung durchgeführte Gebäude- und Wohnungszählung liefert Informationen zu Gebäuden mit Wohnraum sowie zu den darin befindlichen Wohnungen. Zu rein gewerblich genutzten Objekten werden keine Informationen angeboten.

Durch das statistische Verfahren der Haushaltegenerierung stehen nicht nur die gesammelten Informationen zu Bevölkerung, Gebäuden und Wohnungen zur Verfügung, sondern zusätzlich auch Angaben zu Haushalts- und Familienstrukturen. Dabei wurden anschriftenweise Personen zu Haushalten zusammengefasst und den in der Gebäude- und Wohnungszählung erfassten Wohnungen zugeordnet. Die Haushaltegenerierung bildet daher die Wohnhaushalte an einer Anschrift ab. Die Zahl der Haushalte kann von der Zahl der bewohnten Wohnungen abweichen, da ein Haushalt beispielsweise bei Umzügen vorübergehend mehrere Wohnungen nutzen kann.

Für Gemeinden, die weniger als 10 000 Einwohnerinnen und Einwohner aufwiesen, gibt es aufgrund der rechtlichen Vorgaben und aus methodischen Gründen keine Auswertungen zu Migration, Bildung und Erwerbstätigkeit. Für Gemeinden mit mindestens 10 000 Einwohnern sowie flächendeckend ab Kreis- bis zur Bundesebene können hingegen auch die Ergebnisse der Haushaltebefragung auf Stichprobenbasis ausgewiesen werden. Die Zuordnung der Gemeinden zu einer Gemeindegrößenklasse nach § 2 Abs. 6 ZensG 2011 erfolgte anhand der amtlich fortgeschriebenen Bevölkerungszahl zum 31. Dezember 2009 bzw. 1. Januar 2010.

Die in der Zensusdatenbank veröffentlichten Ergebnisse zur Bevölkerung, Haushalten und Familien wurden wie folgt ermittelt:

- Für Gemeinden mit weniger als 10 000 Einwohnerinnen und Einwohner basieren die demografischen Ergebnisse und Ergebnisse zur Religion auf den Angaben aus den Melderegistern, korrigiert um die Ergebnisse aus der Befragung zur Klärung von Unstimmigkeiten und der Mehrfachfallprüfung. Für Gemeinden mit mindestens 10 000 Einwohnerinnen und Einwohner basieren die demografischen Ergebnisse auf den Angaben aus den Melderegistern, korrigiert um die Ergebnisse aus der Haushaltebefragung auf Stichprobenbasis und der Mehrfachfallprüfung.
- In Wohnheimen und Gemeinschaftsunterkünften (Sonderbereiche) wurde eine Vollerhebung durchgeführt, die ebenfalls zu einer Korrektur der ggf. abweichenden Angaben aus den Melderegistern führte.
- Die Ergebnisse zu Haushalts- und Familienmerkmalen wurden im Rahmen der Haushaltegenerierung ermittelt.
- Bei erwerbsstatistischen Auswertungen werden Auszählungen von Erwerbsregisterangaben mit Hochrechnungen der Ergebnisse aus der Haushaltebefragung auf Stichprobenbasis kombiniert.
- Die zu den Themenbereichen Religion (ausführlich), Migration und Bildung veröffentlichten Ergebnisse basieren auf der Hochrechnung der Stichprobenbefunde aus der Haushaltebefragung.

Die hochgerechneten Ergebnisse werden auf volle zehn Personen gerundet ausgewiesen. Die in den Ergebnistabellen dargestellten Summenwerte werden stets auf Basis der nicht gerundeten Ausgangswerte ermittelt, weshalb diese von der Summe der ausgewiesenen Einzelwerte abweichen können.

Bei den hochgerechneten Zensusergebnissen aus der Haushaltsstichprobe werden die Ergebnisse mit zu geringen Besetzungszahlen nicht ausgewiesen, sondern durch einen Schrägstrich („/“) ersetzt.

Bei allen Veröffentlichungen der amtlichen Statistik in Deutschland sind die Einzelangaben der Befragten grundsätzlich geheim zu halten (§ 16 Bundesstatistikgesetz). Bei allen Ergebnisbestandteilen aus Registerauszählungen oder Vollerhebungen des Zensus 2011 erfolgt die Geheimhaltung durch das maschinelle, datenverändernde Verfahren SAFE (Verfahren zur **s**icheren **A**nonymisierung **f**ür **E**inzeldaten). Dieses Verfahren stellt sicher, dass bereits vor Erstellung einer Ergebnistabelle kein Datensatz vorhanden ist, welcher Rückschlüsse auf Einzelfälle zulässt.

Die Einwohnerzahl (Bevölkerung insgesamt) wird durch die statistische Geheimhaltung nicht verändert. Aus diesem Grund kann die Summe der Einzelergebnisse einer Tabelle von der Einwohnerzahl abweichen.

Weiterführende methodische Informationen zum Zensusmodell und zur Geheimhaltung stehen unter www.zensus2011.de zur Verfügung.

Optimierung des Berechnungsverfahrens für die Ausgangsdaten der Bevölkerungsfortschreibung aus dem Zensus 2011

Die Statistischen Ämter des Bundes und der Länder haben die Berechnung der aus dem Zensus 2011 resultierenden Ausgangsdaten für die Bevölkerungsfortschreibung optimiert. Die durch den Zensus 2011 ermittelten und bereits im Jahr 2013 veröffentlichten Einwohnerzahlen sind von der nunmehr vorgenommenen Nachjustierung nicht betroffen und bleiben unverändert.

Das Verfahren zur Hochrechnung von Ergebnissen aus der Haushaltebefragung im Zensus 2011 war auf eine präzise Ermittlung der Gesamteinwohnerzahl der Gemeinden ausgerichtet.

Für Gemeinden mit 10 000 und mehr Einwohnern sind aus diesem Grund bei demografisch fein differenzierten Zensusergebnissen – insbesondere in bestimmten Kombinationen von Altersstufe und Geschlecht – teilweise auffällige Ergebnisse zustande gekommen.

Um nachteilige Konsequenzen für die laufende Bevölkerungsfortschreibung und die Berechnung demografischer Maße wie Geburten- und Sterbeziffern, die auf diesen Angaben aufbauen, auszuschließen, wurde die Kohortenberechnung (Aggregate der fortschreibungsrelevanten demografischen Untergliederungen) optimiert, welche die Auffälligkeiten in den Bevölkerungsstrukturen bereinigt, die Einwohnerzahlen aber unverändert lässt.

Verwendete Symbolik

Symbol	Erläuterung
-	Nichts vorhanden[1)]
/	Keine Angabe, da Zahlenwert nicht sicher genug
0	Weniger als die Hälfte von 1 in der letzten besetzten Stelle, jedoch mehr als nichts
( )	Aussagewert eingeschränkt, da der Zahlenwert durch das Geheimhaltungsverfahren relativ stark verändert wurde
.	Keine Angabe, da der Zahlenwert geheim zu halten ist oder durch das Geheimhaltungsverfahren zu stark verändert wurde.
Abweichungen in den Summen durch Runden der Zahlen	

1) Dies kann ggf. durch das angewendete Geheimhaltungsverfahren bedingt sein.
